# Parents’ Instrumental Family Support, Diet Quality, and Satisfaction with Food-Related Life: A Dyadic Approach in Dual-Earner Parents and Their Adolescent Children

**DOI:** 10.3390/nu18162646

**Published:** 2026-08-13

**Authors:** Berta Schnettler, Andrés Concha-Salgado, Ligia Orellana-Calderón, Mahia Saracostti, Katherine Beroíza, Héctor Poblete, Leonor Riquelme-Segura, Germán Lobos, Cristian Adasme-Berríos, María Lapo

**Affiliations:** 1 Facultad de Ciencias Agropecuarias y Medioambiente, Universidad de La Frontera, Temuco 4811230, Chile; 2 Centro de Excelencia en Psicología Económica y del Consumo, Universidad de La Frontera, Temuco 4811230, Chile; ligia.orellana@ufrontera.cl (L.O.-C.); k.beroiza01@ufromail.cl (K.B.); hector.poblete@ufrontera.cl (H.P.); 3 Scientific and Technological Bioresource Nucleus (BIOREN-UFRO), Universidad de La Frontera, Temuco 4811230, Chile; 4 Facultad de Economía y Empresa, Universidad Católica de Santiago de Guayaquil, Guayaquil 090150, Ecuador; maria.lapo@cu.ucsg.edu.ec; 5 Departamento de Psicología, Universidad de La Frontera, Temuco 4811230, Chile; andres.concha@ufrontera.cl; 6 Departamento de Trabajo Social, Universidad de Chile, Santiago 8330014, Chile; mahia.saracostti@uchile.cl; 7 Departamento de Trabajo Social, Universidad de La Frontera, Temuco 4811230, Chile; leonor.riquelme@ufrontera.cl; 8 Facultad de Economía y Negocios, Universidad de Talca, Talca 3460000, Chile; globos@utalca.cl; 9 Departamento de Economía y Administración, Universidad Católica del Maule, Talca 3466132, Chile; cadasme@ucm.cl

**Keywords:** instrumental family support, diet quality, satisfaction with food-related life, dual-earner couples, adolescents

## Abstract

**Background:** Research on family support has primarily focused on emotional aspects, paying less attention to instrumental support and its concurrent association with food-related well-being. **Aim:** To explore direct and indirect actor and partner effects among parents’ instrumental family support, diet quality, and satisfaction with food-related life (SWFoL) in dual-earner families with adolescents. **Methods:** Online surveys were conducted with 860 dual-earner parent dyads and one adolescent child (mean age 12.7 ± 1.8 years; 49.8% female) in Chile. Family members completed the Adapted Healthy Eating Index and the SWFoL Scale; parents also answered an Instrumental Support subscale. Analyses were conducted using a mediation Actor–Partner Interdependence Model via structural equation modeling. **Results:** Mothers’ and fathers’ instrumental support was positively associated with their own SWFoL, both directly and indirectly via their diet quality. Parents’ support was also positively related to adolescents’ SWFoL directly and indirectly via adolescents’ diet quality. Symmetrical crossover effects between instrumental support and SWFoL emerged for both parents. Mothers’ support was additionally associated with fathers’ SWFoL indirectly through fathers’ diet quality. **Conclusions:** Instrumental support operates as a vital systemic resource within dual-earner households. By enhancing diet quality, tangible support protects family food-related well-being and embeds adolescents’ nutritional health within the parental resource ecosystem.

## 1. Introduction

In dual-earner families, both parents face the dual challenge of fulfilling competing demands from the workplace and the household. When occupational pressures intensify, valuable personal and family resources are depleted, making reliance on family support paramount for maintaining domestic functioning [1]. To successfully meet these family obligations, both mothers and fathers in dual-earner couples require a support system; however, while emotional support remains necessary, instrumental assistance—such as the practical sharing of household chores and caregiving responsibilities—is particularly important for alleviating the burden [2]. According to the Conservation of Resources (COR) theory, instrumental family support serves as an environmental resource that aligns with how individuals preserve their own vital resources, such as time and energy, thereby relating to subjective well-being [3,4].

However, much of the current literature has either combined emotional and instrumental support into a single measure (e.g., [5]) or focused primarily on emotional support in relation to general life, family, or job satisfaction (e.g., [6,7,8]). Consequently, the specific associations of instrumental family support in isolation remain largely overlooked. This omission is especially relevant regarding food-related well-being and adolescent development. Satisfaction with food-related life (SWFoL) refers to a person’s overall cognitive assessment of their food and eating habits, covering meal planning, shopping, meal preparation, consumption, and disposal [9]. While a previous study reported that family emotional support is positively associated with diet quality and SWFoL [10], no research to date has examined the role of instrumental support in this domain, nor how these parental resource dynamics cross over to relate to adolescents’ nutritional outcomes.

This gap is central to understanding nutritional health during adolescence—a critical window for growth and the establishment of lifelong dietary habits [11]. For dual-earner parents, heavy work demands often coexist with lower household diet quality because personal resources such as time and energy are channeled into professional duties rather than food-related tasks (e.g., [12,13]). In this context, instrumental family support corresponds to a greater facilitation of healthier home-cooked meals. Therefore, parental instrumental support may serve as a primary environmental resource that aligns with maintaining the family’s nutritional environment, positioning diet quality as the logical underlying mechanism linking tangible domestic assistance to higher SWFoL, not only for the parents but also for their adolescent children.

Furthermore, the dynamics of family and nutritional support are highly sensitive to cultural contexts. Within collectivistic frameworks such as Chile, family support plays a critical role in correlating with well-being [14,15]. In these environments, adolescents are not merely passive recipients of dietary care; they can offer substantial practical and instrumental support to their parents, including active collaboration in food-related activities [16]. This participation is a widespread structural reality in contemporary Chile. According to the 2023 National Survey of Activities of Children and Adolescents (EANNA), Chilean adolescents regularly perform unpaid domestic chores and care work at home [17]. This collaborative dynamic suggests that instrumental support within the household functions as an interdependent system in which resources are shared across generations, showing concurrent statistical links with the adolescent’s nutritional development and food satisfaction.

To capture these complex, interdependent dynamics, this study adopts a family-systems approach. Drawing on the COR theory, the aims of this research were to explore the direct and indirect actor and partner (crossover) effects between parents’ instrumental family support and the diet quality and SWFoL of mothers, fathers, and adolescents in different-sex dual-earner families in Chile. To achieve this, we utilized a mediation Actor–Partner Interdependence Model (APIMeM) [18], treating the family as an interconnected triad to specifically highlight how parental resource management corresponds to adolescent nutritional well-being.

The original and valuable contribution of this research lies in three key aspects. First, it isolates instrumental family support as an independent variable, separating its specific links to food-related well-being from broader emotional support. Second, it extends dyadic research to a triadic framework by incorporating the adolescent child as an active agent in resource crossover within dual-earner households. Finally, it evaluates diet quality as a continuous mediating mechanism linking parental instrumental resources to individual satisfaction with food-related life for all three family members simultaneously.

### 1.1. Hypotheses Development

#### 1.1.1. Parents’ Instrumental Family Support and Family Members’ Diet Quality

**Figure 1:**
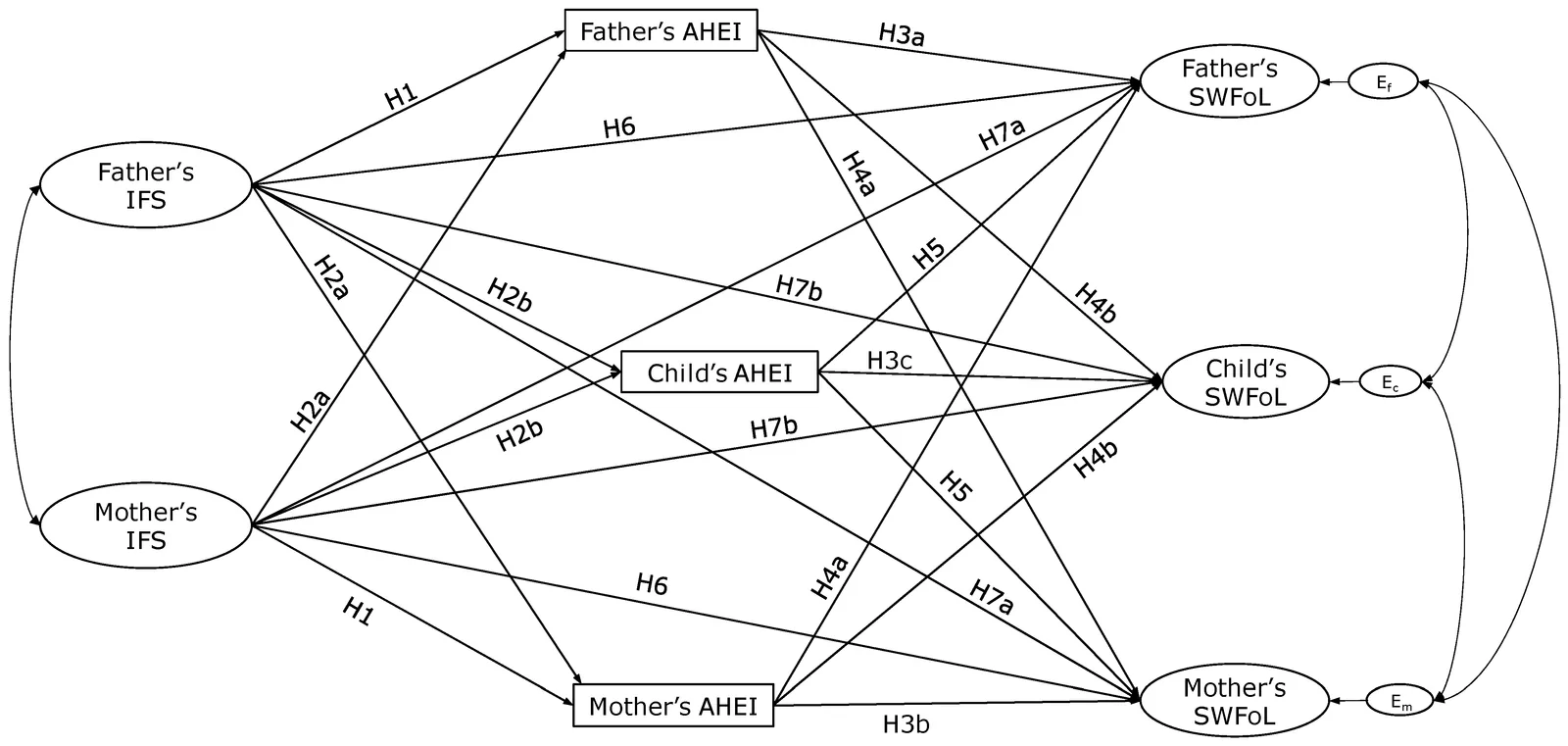
Conceptual model of the proposed actor and partner effects of both parents’ instrumental family support (IFS) on the three family members’ diet quality (measured by the Adapted Healthy Eating Index, AHEI) and Satisfaction with Food-related Life (SWFoL) in dual-earner parents and their adolescents. Notes: Subscripts *f*, *m*, and *c* designate values for fathers, mothers, and adolescent children, respectively. Single-headed solid arrows represent hypothesized structural paths. Double-headed curved arrows represent correlations/covariances between variables. E_f_, E_m_, and E_c_: residual errors on SWFoL for the fathers, mothers, and their adolescent children, respectively. The influence of family socioeconomic status, the number of children, the ages of both parents and adolescents, the working hours and employment types of both parents, and how many times per week family members shared supper was omitted from the path diagram to maintain clarity and avoid clutter. Furthermore, for simplicity, the error covariance for the mediating variable (AHEI) has also been omitted.

Evidence for the association between instrumental family support and diet quality is scant and primarily limited to older adults. Empirical evidence has shown that among older adults, instrumental family support—such as food shopping and meal preparation—is associated with higher diet quality. In that population, aging often limits the ability to shop and cook healthily, yet higher diet quality is frequently observed alongside instrumental support from family (e.g., [19,20,21]). We expect a similar mechanism to occur among dual-earner parents with adolescent children, who are, on average, around 40 years old and in their early midlife. While they may not face age-related physical limitations, these parents suffer from a significant lack of time and energy, as these resources are deeply consumed by their work demands [1]. As a result, they often lack the necessary resources to cook healthy meals for themselves and their families. Consistent with the COR theory [3], instrumental family support is framed as an external resource that aligns with lower depletion of parents’ time and energy. By assisting them with specific, time-consuming tasks like grocery shopping or meal planning, this support relates to the preservation of their vital resources, which is concurrently associated with healthier eating behaviors. Therefore, receiving this tangible support is expected to be associated with better nutritional outcomes for them. Based on this, we propose the first hypothesis (Figure 1):

**H1.**  *Parents’ instrumental family support is positively associated with their own diet quality (actor effects).*

COR theory also accounts for the crossover process, defined as the interpersonal transmission of psychological states or resources, whereby the experiences of one person are linked to the outcomes of another within a close social system [22]. Although previous research has shown that mothers’ emotional support crosses over to relate to fathers’ diet quality [10], to our knowledge, there is no evidence yet of crossover effects between instrumental family support and diet quality among family members. However, it is reasonable to anticipate this relationship. When one parent receives instrumental family support, the resources gained align with healthier meals and better eating behaviors. Because food preparation and consumption in these dual-earner households are largely shared, the benefits of this resource gain are likely to cross over to relate to the rest of the family. In other words, a healthier cooking environment aligns with greater dietary quality of the foods available to both the partner and the adolescent. Therefore, our second hypothesis is as follows (Figure 1):

**H2.**  *Instrumental family support of one parent is positively associated with the diet quality of (a) the other parent and (b) the adolescent (partner effects).*

#### 1.1.2. Family Members’ Diet Quality and Satisfaction with Food-Related Life

According to the Work-Home Resources (W-H R) model [23], which extends COR theory to the family domain, a nutritious diet can be viewed as a contextual resource that corresponds to personal resources, such as physical health and energy. In turn, the accumulation of these personal resources is positively associated with achieving greater satisfaction with food-related life. Empirically, a healthy diet has been consistently linked to higher levels of SWFoL among adults across various contexts [10,24,25,26,27]. However, the findings in adolescents are mixed; while some studies have found no significant relationship [27], others have identified a significant association [10,26]. Given that a high-quality diet is a fundamental resource linked to well-being across the lifespan [9], it is expected to be positively associated with SWFoL for all family members. Thus, the following hypothesis is suggested (Figure 1):

**H3.**  *The quality of one’s diet is positively linked to satisfaction with food-related life for (a) fathers, (b) mothers, and (c) adolescents (actor effects).*

Consistent with the COR theory’s crossover process, previous evidence has shown that mothers’ diet quality is linked to fathers’ SWFoL [10,27] and adolescents’ diet quality is linked to their mothers’ SWFoL [10,26,27]. Consequently, it is expected that one family member’s high-quality diet crosses over to relate to other family members, showing a positive association with SWFoL. Based on these premises, the following hypotheses are posited (Figure 1):

**H4.**  *The quality of one parent’s diet is positively linked to the satisfaction with food-related life of (a) the other parent and (b) the adolescent (partner effects).*

**H5.**  *The quality of adolescents’ diets is positively linked to their parents’ satisfaction with their food-related lives (partner effects).*

It is worth noting that while these actor and partner effects were previously examined by Schnettler et al. [10,27] in relation to a healthy food parenting practice and emotional family support, the present study expands this line of research by focusing on instrumental family support. Given that the APIM framework evaluates how actor and partner effects may be associated with specific characteristics of dyad members and their unique connections [28], these hypotheses were re-evaluated under the expectation that the nature or strength of these relationships might shift when instrumental support is introduced.

#### 1.1.3. Parents’ Instrumental Family Support and Family Members’ Satisfaction with Food-Related Life

Research on dual-earner couples has shown that emotional family support is positively associated with parents’ SWFoL [10]. However, the role of instrumental family support in this specific context remains largely unexplored. While a few studies have linked instrumental family support to general well-being [29], no research to date has examined its association with food-related well-being. This omission is significant because instrumental support has proven relevant in other contexts. For instance, it is strongly associated with older adults’ well-being in Thailand [30] and is the most frequent form of assistance among United States dual-earner couples, where it is associated with lower stress related to the domestic ‘second shift’ [31,32]. Similarly, Jiang and Fung [29] found that instrumental support from mothers was positively associated with well-being in Chinese adult children during the COVID-19 pandemic. According to COR theory, social support acts as a critical environmental resource that helps individuals safeguard their own personal resources and meet daily demands [33]. In dual-earner couples with adolescent children, receiving instrumental family support—such as family members sharing household responsibilities or actively collaborating on food-related tasks—can be associated with lower time pressure and cognitive load for parents. By conserving these personal resources (time and energy), parents are better positioned to efficiently manage food-related activities, such as grocery shopping and cooking healthy meals, which is ultimately associated with higher satisfaction with their food-related life. Based on this evidence, we propose the following hypothesis (Figure 1):

**H6.**  *Instrumental family support is positively linked to satisfaction with food-related life for each parent (actor effects).*

To the authors’ knowledge, there is no evidence of crossover effects between instrumental family support and general well-being among family members, and even less regarding SWFoL. However, consistent with the crossover process proposed by the COR theory—which posits that resources can be transferred within the family system [33]—a previous study found that mothers’ and fathers’ emotional family support crossed over to their adolescent children, showing positive links to their SWFoL [10]. It is reasonable to anticipate similar interpersonal associations for instrumental support. When one parent reports higher instrumental support, this is associated with lower daily strain and more efficient management of household and food-related activities. Empirically, practical help and equitable task-sharing between dual-earner partners have been shown to correspond with lower individual cognitive load and daily stress, which are, in turn, linked to greater domain-specific satisfaction [2]. Furthermore, visible parental instrumental behaviors align with a structured home environment that is positively associated with adolescent domain well-being [26]. According to the resource enrichment and crossover literature [22,23], this individual resource availability is associated with a more positive family environment and facilitates shared activities, such as healthier family meals, which are positively linked to the SWFoL of other family members. On this basis, we posited the following hypotheses (Figure 1):

**H7.**  *Instrumental family support of one parent is positively linked to the satisfaction with food-related life of (a) the other parent, and (b) the adolescent (partner effects).*

#### 1.1.4. Indirect Pathways Through Diet Quality

This research suggests that diet quality serves a mediating role in the relationship between instrumental family support and SWFoL. The interaction among instrumental family support, diet quality, and SWFoL aligns with the principles of COR theory. According to this theory, maintaining a healthier diet can be viewed as a process where resources within the home environment (such as instrumental family support) correspond to a “gain spiral” that aligns with the accumulation of resources. This accumulation is concurrently associated with higher well-being outcomes [3]. Evidence at both individual and interindividual levels supports the mediating role of diet quality. In a study by Schnettler et al. [10], mothers’ diet quality mediated the relationship between their emotional family support and SWFoL. Similarly, the authors noted that mothers’ emotional family support had a positive indirect association with fathers’ SWFoL via their diet quality. This mediating mechanism is even more likely to occur when examining instrumental support rather than emotional support. Because instrumental family support involves tangible assistance—such as sharing household chores, grocery shopping, and collaborating in the kitchen—it is directly linked to the practical execution of food-related tasks. In other words, this hands-on support aligns with the time and energy necessary to prepare healthier meals, making diet quality the most direct and logical pathway through which instrumental assistance is associated with higher SWFoL for the family. As a result, the following hypothesis was proposed, incorporating both actor and partner effects:

**H8.**  *Diet quality serves as a mediator between the instrumental family support from both parents and the satisfaction with food-related life experienced by all three family members.*

## 2. Materials and Methods

### 2.1. Participants and Sample Selection

#### 2.1.1. Inclusion and Exclusion Criteria

Derived from a larger project examining well-being, food resources, and work-family dynamics among two-parent dual-earner families with adolescent children [34], the present study utilized convenience sampling to select an initial pool of 860 families. The inclusion criteria for the target sample were: (1) dual-earner two-parent households (married or cohabiting) where both adult partners were employed; (2) cohabiting with at least one adolescent child aged 10–15 years; and (3) independent participation and survey completion by all three family members. Single-parent families or households where one parent was unemployed were excluded.

#### 2.1.2. Data Screening and Final Sample

While the original cohort comprised 430 families from Temuco city and 430 from Rancagua city, Chile, a subsequent data screening phase was conducted, and 13 cases from Rancagua were excluded because they exhibited consistent or invariant response patterns across the measurement scales. This resulted in a final analytical sample of 847 families (430 from Temuco and 417 from Rancagua).

### 2.2. Measures

The scale below was completed exclusively by mothers and fathers:

The short version of the Inventory of Family Support for Workers [35]: comprises 8 items grouped into two dimensions: emotional support and instrumental support. In the present study, only the 4-item family instrumental support subscale was used (i.e., “When I’m having a difficult week at my job, my family members try to take on more of the household work”, “If my job gets very demanding, someone in my family will take on extra household responsibilities”, “My family members do their fair share of household chores”, “Members of my family cooperate with me to get things done around the house”). Responses were recorded on a 5-point Likert scale, ranging from 1 (totally disagree) to 5 (totally agree). The Spanish validated version was used [36]. Individual item scores were aggregated into a total score, with higher values indicating greater levels of instrumental family support.

The three family members answered the following instruments:

Diet quality is defined in this study as the degree of alignment between an individual’s food consumption and healthy dietary guidelines, evaluated using the Adapted Healthy Eating Index (AHEI). This instrument is a Spanish adaptation of the US-HEI [37], developed by Norte and Ortiz [38], which assesses the frequency of consumption of nine distinct food groups: 1. cereals and derivatives, 2. vegetables, 3. fruits, 4. milk and dairy products, 5. meats, 6. legumes, 7. sausages and cold meats, 8. sweets, and 9. sugar-sweetened soft drinks. Following the guidelines by Norte and Ortiz [38], consumption frequencies for these nine categories are converted into individual scores ranging from 0 to 10 based on adherence to daily and weekly dietary recommendations. A tenth variable, representing dietary variety, is derived from consumption patterns across the nine primary food groups; participants receive two points for fulfilling daily recommendations and one point for meeting weekly recommendations. The total AHEI score is computed by summing all variables, yielding a maximum of 100 points. Total scores are categorized into three diet quality tiers: “healthy” (above 80 points), “requires changes” (51–80 points), and “unhealthy” (50 points or below) [37].

Satisfaction with Food-related Life (SWFoL) scale [9]: is a one-dimensional, five-item scale that measures an individual’s overall assessment of their food and eating habits (i.e., “Food and meals are very positive elements in my life”, “I am generally pleased with my food”, “My life in relation to food and meals is close to ideal”, “With regard to food, the conditions of my life are excellent”, “Food and meals give me satisfaction in daily life”). This study used the Spanish validated version of the scale [39], which has consistently demonstrated robust internal consistency across various Chilean cohorts, including adults, adolescents, and dual-earner parents (e.g., [10,26,27]). Respondents rate their agreement with each item on a 6-point Likert scale, ranging from 1 (completely disagree) to 6 (completely agree). The total SWFoL score is calculated by summing the five items, with higher cumulative scores indicating greater satisfaction with food-related life.

Demographic characteristics and family routines were collected through tailored questions for each respondent. All three family members reported their ages, while the adolescents specified their gender. Parents provided information regarding their employment status, work settings, and weekly working hours. Additionally, mothers reported family structure variables, including total household size and the number of children, as well as food dynamics, such as the weekly frequency of shared family meals (breakfast, lunch, and dinner). Mothers also detailed the weekly frequency of consuming homemade meals, purchasing ready-to-eat food, ordering food delivery, or dining at restaurants and fast-food outlets, as well as the daily hours spent cooking by themselves and their male partners. Lastly, family socioeconomic status (SES) was determined based on total household income, household size, and the education and occupation of the primary earner [40].

### 2.3. Procedure

Parents were contacted via social media and the schools where their adolescents were enrolled. Trained interviewers explained the study’s goals and detailed the structure of the online questionnaires to the parents, highlighting the confidentiality and anonymity of their answers. Typically, interested participants provided their mother’s email address, which was then used to distribute the survey links. Each family member filled out an online questionnaire individually. Furthermore, the interviewers offered phone support to answer questions and help with completing the questionnaires. Data collection took place from April to July in Rancagua and from June to November 2024 in Temuco.

The study was conducted in accordance with the Declaration of Helsinki. Participation was strictly voluntary. Before accessing the questionnaires, adult participants provided informed consent, and adolescents granted assent by ticking the appropriate checkboxes at the beginning of the online session. Data collection was managed via QuestionPro (QuestionPro Inc., Austin, TX, USA), where individual family member responses were isolated in separate databases to maintain confidentiality. To ensure personal data protection and participant anonymity, no direct personal identifiers were stored; instead, a unique family ID code was assigned to merge the responses of the three family members into a single analytical database. A $10 USD bank transfer was issued to each family as compensation once all three assessments were finalized. All procedures were approved and monitored by the Ethics Committee at the Universidad de La Frontera (Protocol No. 035-23).

### 2.4. Data Analysis

To operationalize the study aims via the triadic APIMeM framework, statistical analyses were initiated with descriptive statistics performed in SPSS v.23. Means (M) and standard deviations (SD) were calculated for continuous study variables, and bivariate relationships were examined using Pearson correlation coefficients (r). To evaluate mean differences across family members, a paired-samples *t*-test was conducted for parental variables (instrumental family support), while Repeated Measures Analysis of Variance (ANOVA) with Bonferroni post hoc adjustments was used for variables assessed across all three family members (AHEI and SWFoL).

Subsequently, scale psychometrics were evaluated using factor loadings, Average Variance Extracted (AVE), and McDonald’s omega. Then, a mediation Actor–Partner Interdependence Model (APIMeM) for distinguishable dyads was estimated via Structural Equation Modeling (SEM) [18,41] using Mplus 8.11. Within this framework, the structural model simultaneously evaluated direct actor effects, direct partner (crossover) effects across family members, and indirect (mediational) pathways. Actor effects represent the associations between variables within the same individual, whereas partner effects capture the cross-member influences from one family member to another (i.e., crossover). The dyads analyzed comprised mother-father and parent-adolescent pairs, in which each member simultaneously functioned as both an actor and a partner. Specifically, the tested actor and partner effects involved parental family instrumental support, diet quality (AHEI), and SWFoL for all three family members.

Several confounding and interdependent effects were controlled for within the APIMeM architecture. First, parental modeling influences were accounted for by correlating the respective independent variables across both parents (instrumental family support). Following Kenny et al.’s [18] guidelines, additional sources of dyadic interdependence were controlled by specifying correlations between the residual errors of the dependent variables (AHEI and SWFoL) across the three family members. Furthermore, the model adjusted for the direct effects of family SES, number of children, ages of all three members, parental working hours, employment type, and weekly family supper frequency on the main outcomes.

Due to the ordinal nature of the scale items, SEM parameters were estimated using the Weighted Least Squares Mean and Variance Adjusted (WLSMV) estimator based on a polychoric correlation matrix. Model fit was evaluated using standard criteria [42]: values greater than 0.95 for the Comparative Fit Index (CFI) and the Tucker–Lewis Index (TLI), and values less than 0.06 for the Root Mean Square Error of Approximation (RMSEA). Finally, the mediating role of diet quality was examined through a bias-corrected (BC) bootstrapping procedure with 1000 resamples [43], establishing statistical significance when the BC confidence intervals excluded zero. Direct structural pathways are reported using standardized path coefficients (β) with exact *p*-values (α = 0.05, with thresholds at * *p* < 0.05, ** *p* < 0.01, and *** *p* < 0.001), while indirect pathways are reported with standardized indirect coefficients and their 95% BC bootstrap confidence intervals.

## 3. Results

### 3.1. Sample Description and Preliminary Analyses

Descriptive statistics (M, *SD*, *n*, %) and bivariate Pearson correlation coefficients (r) were computed using SPSS v.23. Mean differences between parents were evaluated using paired-samples *t*-tests, while differences across the three family members were examined via Repeated Measures ANOVA with Bonferroni post hoc adjustments.

Table 1 presents the sociodemographic characteristics of the sample consisting of 847 families with mothers, fathers, and one adolescent. The average age of mothers was 38.5 years, fathers was 41.0 years, and adolescents was 12.7 years. Among the adolescents, 50.2% were male. Typically, families included four members and had two children, with the majority falling into the middle socioeconomic status (SES) category. Most mothers and fathers were employees and worked in person. Regarding the frequency of family meals, families gathered for breakfast, lunch, and dinner more than three times a week, with homemade meals being a common choice. Additionally, mothers spent more hours cooking each day of the week than fathers.

Table 2 presents the average scores and correlations for parental instrumental family support, diet quality (assessed using the AHEI), and SWFoL. All correlations were significant and aligned with expectations. Notably, fathers reported significantly higher levels of instrumental family support compared to mothers (t = −11.609, *p* < 0.001). When it came to AHEI scores, fathers scored significantly lower than both mothers and adolescents (F = 29.789, *p* < 0.001), with no significant difference between mothers and adolescents. However, based on the cutoff established by Kennedy et al. [37], all three family members had average AHEI scores, suggesting that their diet “requires changes.” Additionally, adolescents had significantly higher SWFoL scores than both their mothers and fathers (F = 15.352, *p* < 0.001), while mothers and fathers showed no significant difference in their scores.

### 3.2. APIM Findings: Evaluating Actor–Partner Hypotheses

Measurement properties and structural equation models were estimated using Mplus 8.11 with the WLSMV estimator based on a polychoric correlation matrix. Confirmatory factor loadings, Average Variance Extracted (AVE), and McDonald’s omega were calculated to establish scale validity and reliability before evaluating direct structural paths.

#### 3.2.1. Psychometric Properties and APIMeM Fit

All standardized factor loadings for the scales were statistically significant (*p* < 0.001) and greater than 0.50, confirming convergent validity. The average extracted variance values were also above 0.50, with mothers’ instrumental family support at 0.72, fathers’ instrumental family support at 0.58, mothers’ SWFoL at 0.62, fathers’ SWFoL at 0.60, and adolescents’ SWFoL at 0.62. The omega coefficients revealed a good level of reliability for all scales, with scores of 0.91 for mothers’ instrumental family support, 0.84 for fathers’ instrumental family support, 0.89 for mothers’ SWFoL, 0.88 for fathers’ SWFoL, and 0.89 for adolescents’ SWFoL.

#### 3.2.2. Direct Actor and Partner Effects

**Figure 2:**
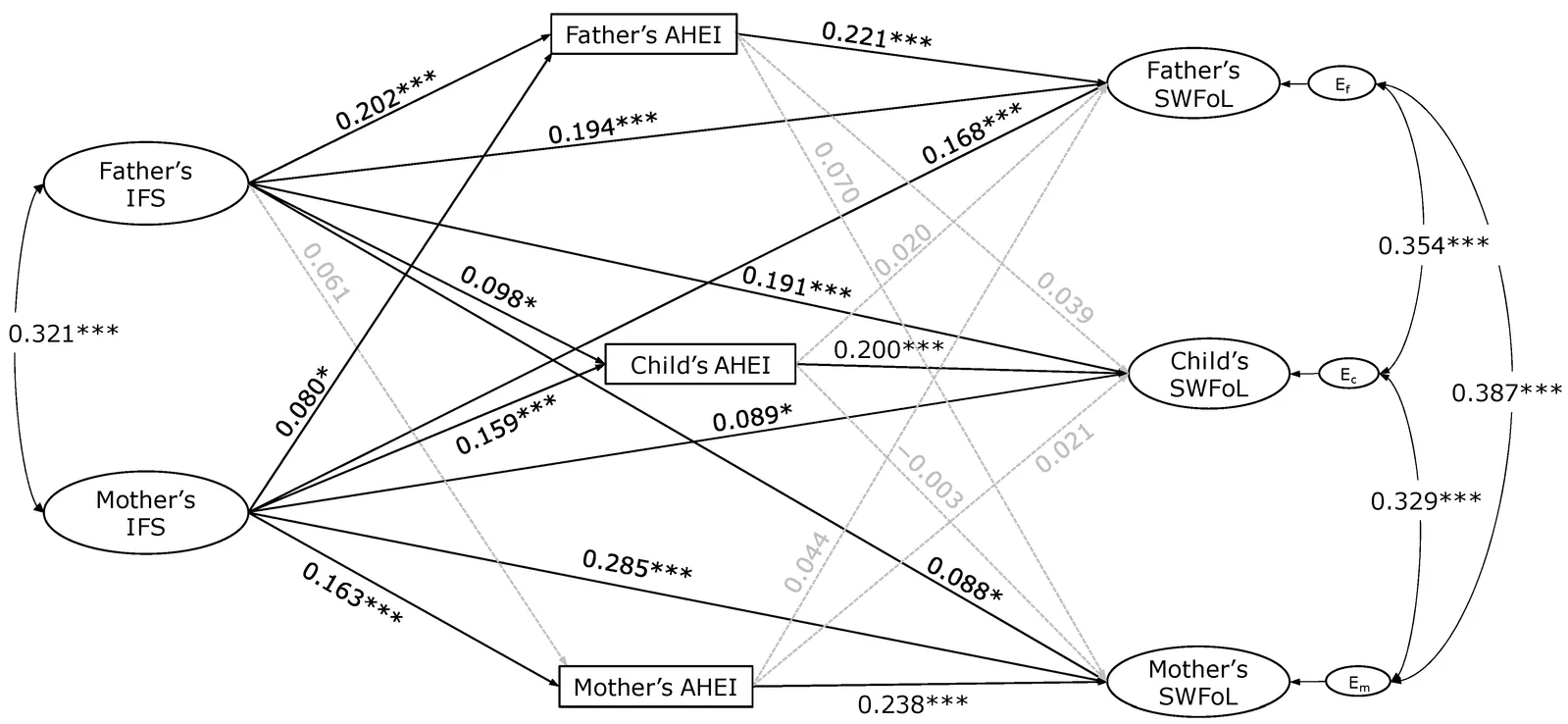
Mediation actor–partner interdependence model of the effect of both parents’ instrumental family support on the three family members’ diet quality (measured by the Adapted Healthy Eating Index, AHEI) and Satisfaction with Food-related Life (SWFoL) in dual-earner parents and their adolescents. Notes: Subscripts f, m, and c designate values for fathers, mothers, and adolescent children, respectively. Single-headed solid arrows represent hypothesized structural paths. Double-headed curved arrows represent correlations/covariances between variables. E_f_, E_m_, and E_c_: residual errors on SWFoL for the fathers, mothers, and their adolescent children, respectively. * *p* < 0.05, *** *p* < 0.001. The influence of family socioeconomic status, the number of children, the ages of both parents and adolescents, the working hours and employment types of both parents, and how many times per week family members shared supper was omitted from the path diagram to maintain clarity and avoid clutter. Furthermore, for simplicity, the error covariance for the mediating variable (AHEI) has also been omitted.

The findings from the APIMeM estimation are illustrated in Figure 2. The model evaluating the relationships between instrumental family support from mothers and fathers and the AHEI and SWFoL of the three family members demonstrated a strong fit to the data (CFI = 0.972; TLI = 0.963; RMSEA = 0.045 [90% CI: 0.042, 0.048]). A significant correlation was observed in instrumental family support from both parents (r = 0.331, *p* < 0.001). Additionally, significant correlations were observed in the residual errors of mothers’ and fathers’ AHEI (r = 0.434, *p* < 0.001), between mothers’ and adolescents’ AHEI (r = 0.508, *p* < 0.001), and between fathers’ and adolescents’ AHEI (r = 0.340, *p* < 0.001). Similarly, significant correlations were found in the residual errors of mothers’ and fathers’ SWFoL (r = 0.387, *p* < 0.001), between mothers’ and adolescents’ SWFoL (r = 0.329, *p* < 0.001), and between fathers’ and adolescents’ SWFoL (r = 0.354, *p* < 0.001).

The standardized path coefficients reveal that both fathers’ (β = 0.202, *p* < 0.001) and mothers’ (β = 0.163, *p* < 0.001) instrumental family support were positively correlated with their own AHEI. This finding confirmed H1 for both parents. Additionally, mothers’ instrumental family support was positively related to fathers’ AHEI (β = 0.080, *p* = 0.037), while fathers’ instrumental family support did not show a significant relationship with mothers’ AHEI (β = 0.061, *p* = 0.139). Furthermore, both fathers’ (β = 0.098, *p* = 0.013) and mothers’ (β = 0.159, *p* < 0.001) instrumental family support were positively linked to adolescents’ AHEI. These results supported H2a for mothers and H2b for both mothers and fathers.

The findings suggest a positive association between fathers’ (β = 0.221, *p* < 0.001), mothers’ (β = 0.238, *p* < 0.001), and adolescents’ AHEI (β = 0.200, *p* < 0.001) and their respective SWFoL. These findings supported H3 for all three family members (H3a, H3b, and H3c). There was no significant association between fathers’ AHEI and mothers’ SWFoL (β = 0.070, *p* = 0.058), nor with their adolescent children’s SWFoL (β = 0.039, *p* = 0.350). Similarly, mothers’ AHEI did not show a significant relationship with fathers’ SWFoL (β = 0.044, *p* = 0.288) or adolescents’ SWFoL (β = 0.021, *p* = 0.645). These results did not support hypotheses H4a and H4b. In a similar vein, the quality of adolescents’ diets showed no significant association with fathers’ (β = 0.020, *p* = 0.631) or mothers’ (β = −0.003, *p* = 0.944) SWFoL, leading to the conclusion that hypothesis H5 was not upheld.

The results indicate that both fathers’ (β = 0.194, *p* < 0.001) and mothers’ (β = 0.285, *p* < 0.001) instrumental family support were positively related to their own SWFoL. These findings corroborated H6 for both parents. Fathers’ instrumental family support was positively associated with mothers’ SWFoL (β = 0.088, *p* = 0.041). Likewise, mothers’ instrumental family support was significantly related to the fathers’ SWFoL (β = 0.168, *p* < 0.001). Fathers’ (β = 0.191, *p* < 0.001) and mothers’ (β = 0.089, *p* = 0.044) instrumental family support were positively associated with the adolescents’ SWFoL. These findings supported H7a and H7b.

#### 3.2.3. Control Variables

Most of the control variables did not significantly affect the model. The family SES positively affected the mothers’ (β = 0.170, *p* < 0.001), the fathers’ (β = 0.129, *p* < 0.001), and the adolescents’ (β = 0.071, *p* = 0.042) AHEI as well as the adolescents’ SWFoL (β = 0.092, *p* = 0.015). Namely, mothers, fathers, and adolescents from high-SES backgrounds had higher AHEI scores than those from lower-SES backgrounds. A similar interpretation holds for family SES and adolescents’ SWFoL. Adolescents’ age negatively affected their AHEI (β = −0.148, *p* < 0.001) and SWFoL (β = −0.092, *p* = 0.022). The mothers’ weekly working hours negatively affected their SWFoL (β = −0.092, *p* = 0.022), whereas the fathers’ weekly working hours negatively affected their AHEI (β = −0.071, *p* = 0.027). The fathers’ employment type positively affected their SWFoL (β = 0.081, *p* = 0.034), indicating that self-employed fathers had higher SWFoL scores than employed fathers. Lastly, the number of family supper times per week positively affected the mothers’ (β = 0.108, *p* = 0.007), fathers’ (β = 0.124, *p* < 0.001), and adolescents’ (β = 0.092, *p* = 0.010) SWFoL and their AHEI (β = 0.131, *p* < 0.001).

### 3.3. Examining the Mediating Role of Dietary Quality

Indirect mediational pathways were tested in Mplus 8.11 using a bias-corrected bootstrapping procedure with 1000 resamples to estimate 95% confidence intervals (95% CI).

At the individual level, the findings indicated that the quality of mothers’ diet (measured by the AHEI score) acted as a mediator in the relationship between their instrumental family support and SWFoL, as evidenced by a significant indirect association obtained through bootstrapping confidence intervals (standardized indirect effect = 0.039, 95% CI = 0.020, 0.068). Similarly, the fathers’ diet quality served as a mediator in the relationship between their instrumental family support and SWFoL (standardized indirect effect = 0.045, 95% CI = 0.025, 0.073). At the interindividual level, the quality of fathers’ diets mediated the connection between mothers’ instrumental family support and the fathers’ SWFoL (standardized indirect effect = 0.018, 95% CI = 0.002, 0.036). Additionally, adolescents’ diet quality mediated the relationship between mothers’ instrumental family support and the adolescents’ SWFoL (standardized indirect effect = 0.032, 95% CI = 0.015, 0.061). Likewise, adolescents’ diet quality mediated the connection between fathers’ instrumental family support and the adolescents’ SWFoL (standardized indirect effect = 0.020, 95% CI = 0.005, 0.039). No other indirect effect of diet quality was identified, as the confidence intervals included zero (Table 3). These results partially supported the mediating role of mothers’, fathers’, and adolescents’ diet quality in the relationship between parents’ instrumental family support and the SWFoL of all three family members.

## 4. Discussion

### 4.1. Rationale for the Present Study Design

For dual-earner parents, demanding job responsibilities often coexist with lower household diet quality as time and physical energy are diverted from food management to workplace obligations [12,13]. While previous research examined how affective family resources relate to nutritional well-being [10], evaluating practical, task-oriented assistance required an analytical framework capable of capturing systemic family dynamics. To address this gap, the present study adopted a triadic mediation Actor–Partner Interdependence Model (APIMeM) design within different-sex dual-earner families with adolescent children in Chile. This design was chosen specifically because food management in households with working parents functions as an interdependent system in which individual resources, behaviors, and well-being outcomes are concurrently linked among family members [18,23]. By analyzing mother–father–adolescent triads simultaneously, this approach allowed us to disentangle individual actor effects from interpersonal crossover partner pathways, providing a comprehensive assessment of how parents’ instrumental support relates to dietary quality and food-related life satisfaction across the entire family system.

Using a mediation APIM approach in different-sex dual-earner families, our results showed that mothers’ and fathers’ instrumental family support is positively associated with both parents’ and adolescents’ SWFoL, both directly and indirectly through diet quality. These findings underscore that tangible, task-oriented assistance within the home is a valuable resource for members beyond the parents, directly corresponding to adolescents’ nutritional environments. The sections that follow explore these relationships in depth, focusing on actor and partner effects and the mediating role of diet quality.

### 4.2. Main Findings and Theoretical Interpretation

#### 4.2.1. Parents’ Instrumental Family Support and Family Members’ Diet Quality

Consistent with the COR theory [3], mothers’ and fathers’ instrumental family support was positively associated with their own diet quality (H1 supported). Evidence for this association has been scant and primarily limited to older adults, in whom instrumental family support corresponds with maintaining healthy eating habits by overcoming age-related physical limitations [19,20,21]. Our results expand this knowledge by demonstrating that a similar mechanism occurs among dual-earner parents. Although these parents do not face age-related physical decline, they report a severe lack of time and energy due to high work demands [1]. In this scenario, instrumental family support is associated with lower depletion of parents’ vital resources. By providing assistance with specific, time-consuming tasks like grocery shopping or meal planning, this support relates to the preservation of personal resources, which is concurrently associated with healthier eating behaviors. This resource framework is highly visible in our sample, where families report eating homemade food an average of 6 days per week, with mothers spending 2.4 h per day cooking during the week and fathers spending 1.6 h per day (Table 1). Managing this significant domestic workload corresponds to substantial time and physical effort; therefore, receiving tangible, task-oriented support is positively associated with maintaining better diet quality for both parents. Nonetheless, an interesting nuance emerged in the strength of these paths, as the association was stronger for fathers than for mothers. This pattern may relate to how genders are traditionally socialized regarding resource utilization within the household. While fathers may tend to channel received instrumental support more directly toward their individual benefit—such as improving their own eating habits—mothers often operate under a family-oriented caregiving framework. Research on family dynamics suggests that women are more likely to collectivize domestic resources to optimize overall family well-being rather than focusing exclusively on their personal needs [44,45]. Consequently, when mothers receive practical help, they may allocate this resource to improve overall family feeding dynamics, thereby diluting the direct association with their own dietary outcomes. By contrast, because fathers are more likely to channel this support into individualized benefits, the statistical link between instrumental support and their own diet quality remains more pronounced.

Regarding partner effects, mothers’ instrumental family support was positively associated with fathers’ diet quality, but not vice versa (H2a partially supported). This asymmetry aligns with previous findings in which mothers’ emotional family support showed a crossover association with fathers’ diet quality, whereas fathers’ support showed no association with mothers’ dietary outcomes [10]. This gender pattern in crossover relationships can be understood by considering how traditional gender roles interact with resource allocation within the COR framework, where the value and relevance of a resource depend on the specific demands an individual faces [3]. Since mothers in Chile frequently retain the primary responsibility for the family’s nourishment [46], their family routines are deeply shaped by the second-shift phenomenon [31], in which women continue to bear the disproportionate mental and physical load of household management alongside formal employment. In this context, the instrumental support mothers receive corresponds to lower structural constraints, which relate to more efficient management of the household’s core meals. Because meal preparation serves as a shared resource within the home, the mother’s reported availability of resources is concurrently associated with better dietary quality for both her partner and her children (supporting H2b). By contrast, the lack of a crossover link from fathers’ instrumental support to mothers’ diet quality reflects gendered differences in household labor allocation: practical support reported by fathers may be channeled into discrete, non-dietary domestic tasks or individual activities rather than systemic meal management. This would explain why fathers’ instrumental support is observed alongside their own dietary benefits (H1) and is concurrently linked to the adolescents (H2b), but does not show a significant relationship with the mother. Notably, distinct patterns emerged in the strength of the crossover paths toward youth, with the association between mothers’ instrumental family support and adolescents’ diet quality stronger than the pathway from fathers to adolescents. This difference is consistent with the premise that mothers tend to utilize resources to optimize overall family nutrition [44,45], making their practical support a more sensitive predictor of their children’s dietary quality. In any case, these findings expand current knowledge regarding adolescents. While prior evidence showed that parents’ emotional support lacked crossover relationships with adolescents’ diet quality [10], the current results demonstrate that tangible, task-oriented assistance is associated with favorable indicators within the food environment. Finally, a notable nuance emerged regarding fathers, who reported higher instrumental support than mothers yet displayed lower overall diet quality than both mothers and adolescents. Guided by gender-role and second-shift literature [31,47], this pattern likely reflects the ‘marital support gap,’ in which fathers perceive high levels of practical assistance, largely provided by their partners. However, because the core management of family nutrition remains centered on mothers, receiving this domestic support does not automatically translate into healthier personal food choices for fathers, which helps explain why their overall diet quality remains lower than that of the rest of the household.

#### 4.2.2. Family Members’ Diet Quality and Satisfaction with Food-Related Life

Consistent with the W-H R model [23] and the relevance of a high-quality diet as a resource for well-being across the lifespan [9], our results show that diet quality was positively associated with SWFoL for the mothers, fathers, and adolescents (H3a, H3b, and H3c supported). This result in mothers and fathers is in line with previous studies in adult samples in different contexts [10,24,25,26,27]. For adolescents, the positive association between diet quality and SWFoL aligns with some previous Chilean studies [10,26] but contradicts another that reported a null association [27]. Qualitative evidence indicates that adolescent SWFoL is dual-driven: it relates to both healthy eating at home and to consuming tasty, less nutritious foods with peers [48]. The data for the 2022 study [27] were collected during strict pandemic lockdowns, when adolescents were isolated from their friends [49]. This lack of choice likely explains why eating healthily was not associated with higher satisfaction reports; they were limited to home-cooked food and were missing the social and hedonic aspects of eating [50]. By contrast, under the normal conditions of our study, adolescents experience more freedom. They can balance healthy eating at home with social food choices outside, allowing a higher-quality household diet to coexist with higher SWFoL.

Contrary to expectations, crossover effects between diet quality and SWFoL were absent among all family members. Neither mothers’ nor fathers’ diet quality was linked to their partner’s SWFoL (H4a not supported), and neither parent’s diet quality was associated with the adolescents’ SWFoL (H4b not supported). Similarly, the adolescents’ diet quality was not related to their parents’ SWFoL (H5 not supported). These results contrast with previous studies in which the precursors were a healthy food parenting practice or emotional family support [10,27]. In those studies, the diet quality of one family member was associated with higher reports of SWFoL in another [10,27]. This divergence can be understood in light of the distinct nature of the resources involved. Emotional support and food parenting practices are affective and relational resources; therefore, observing a family member eat healthily because of one’s emotional investment is directly tied to shared psychological well-being. By contrast, instrumental family support is strictly pragmatic and task-oriented [35]. Under the COR theory, resources are deployed to meet specific environmental demands, and their primary systemic function is fulfilled once that structural strain is alleviated [15]. In this case, once the tangible resource co-occurs with overcoming time constraints and better diet quality, its immediate role ends. The subsequent link between a high-quality diet and SWFoL becomes an individual experience (an actor effect) rather than an interpersonal one. In dual-earner households, while the logistics of healthy eating are shared, the psychological well-being derived from nutrition is experienced individually. These findings confirm that APIM outcomes depend heavily on how specific resource characteristics shape the connections within the triad [28]. In summary, while practical assistance facilitates the logistical execution of a healthy diet across the family, the subjective evaluation of food-related life satisfaction remains confined to the individual consumer, which is consistent with the absence of direct dietary crossover effects on well-being.

#### 4.2.3. Parents’ Instrumental Family Support and Family Members’ Satisfaction with Food-Related Life

Consistent with the COR theory [33], instrumental family support was positively associated with SWFoL for both parents (H6 supported). This finding matches with prior research indicating that emotional family support is also positively associated with parents’ SWFoL [10], suggesting that different types of family resources serve as concurrent pillars for food-related well-being. Under the resource conservation framework, these findings suggest that higher levels of tangible assistance within the household may be associated with the preservation of parents’ vital resources, such as time and energy. Consequently, this preservation of personal resources is concurrently linked to a greater SWFoL, as it coexists with a lower daily strain in managing household demands. This result expands current knowledge by demonstrating that instrumental family support is linked not only to general well-being [29,30] but also to well-being within the food domain. However, an interesting nuance emerged regarding gender. Although fathers in this sample reported higher levels of instrumental support, the positive association between this support and SWFoL was visibly stronger for mothers. Under the second-shift framework [31,32], instrumental support is associated with both partners’ well-being but shows a higher psychological return for women. Because mothers in Chile still carry the primary cultural and practical responsibility for family feeding [46], the presence of practical help is more strongly linked to their SWFoL, as reflected in the mitigation of stress associated with their traditional gender role.

Consistent with the resource enrichment and crossover framework [22,23], the findings revealed that mothers’ instrumental family support was positively associated with fathers’ SWFoL, and vice versa (H7a supported), while both parents’ instrumental support was concurrently linked to the adolescents’ SWFoL (H7b supported for both parents). These results suggest that when one parent has practical help, individual resource availability is associated with lower cumulative strain and a more positive family environment, which in turn is associated with SWFoL among all family members. These findings are consistent with empirical evidence demonstrating that practical help and task-sharing between dual-earner partners are associated with reduced cognitive load and daily strain, thereby showing positive links to domain-specific satisfaction [2]. Similarly, visible parental instrumental behaviors are associated with a structured domestic environment, which is positively associated with adolescent well-being [26]. To the authors’ knowledge, this is the first study to report crossover effects of instrumental family support within the family system toward any well-being domain. By demonstrating these interpersonal links, the current results expand the literature. While the crossover toward adolescents is consistent with prior evidence that both parents’ emotional family support is linked to their adolescent children’s SWFoL [10], these findings also indicate that tangible, task-oriented resources are positively associated with interpersonal well-being within the triad, particularly between partners. Nonetheless, distinct gender patterns emerged in the strength of these relationships. The association between mothers’ instrumental support and fathers’ SWFoL was stronger than the inverse pathway. In contexts where domestic responsibilities tend to fall mainly on women, as in Chile [46], practical support from fathers and children appears particularly relevant to mothers. This resource availability relates to a more balanced management of work and family obligations for women [22], which concurrently shows a tighter, more sensitive statistical link to the partner’s SWFoL. This stronger association suggests that when the primary manager of family food tasks experiences reduced strain, the positive interpersonal crossover toward the partner is more pronounced, even though overall levels of SWFoL remain statistically similar between parents. By contrast, the association between fathers’ instrumental support and adolescents’ SWFoL was stronger than that observed for mothers. This outcome suggests that the tangible support reported by fathers may be more often directed toward specific, high-visibility tasks that directly involve adolescents, as previously discussed.

#### 4.2.4. Indirect Pathways Through Diet Quality

Lastly, consistent with the principles of the COR theory [3], the findings support a “gain spiral” framework, in which instrumental family support, diet quality (i.e., AHEI score), and SWFoL are concurrently and positively linked among the three family members. At the individual level (actor effects), both parents’ diet quality served as an underlying connecting mechanism between their perceived instrumental family support and their own SWFoL. These findings partially align with prior evidence showing that mothers’ diet quality mediated the relationship between their emotional family support and food-related well-being [10]; however, the current results expand this knowledge by demonstrating a similar indirect pathway for fathers. At the interindividual level (partner effects), mothers’ instrumental family support was indirectly associated with fathers’ SWFoL via fathers’ own diet quality (β = 0.018, *p* = 0.049). In this crossover pathway, the practical support provided by mothers relates to better dietary quality for their partners, which in turn acts as the connecting actor mechanism linking fathers’ own food-related life satisfaction. Furthermore, the current results extend the literature by revealing that adolescents’ diet quality also served as a connecting mechanism between both parents’ instrumental family support and adolescents’ own SWFoL. Taken together, these results suggest that higher levels of hands-on support within the household are connected with the availability of time and energy necessary for healthier food management. Consequently, diet quality emerges as a prominent indirect pathway through which instrumental assistance is associated with higher SWFoL across the entire family triad.

### 4.3. Limitations

The limitations of this study should be considered. Firstly, the cross-sectional design restricts the capability to determine the direction of the relationships, while the use of non-probabilistic convenience sampling limits the generalizability of the findings to the broader population of Chile. To gain a deeper insight into the directional dynamics between the variables studied, future longitudinal or cross-lagged research is highly recommended. Additionally, adopting probabilistic sampling methods in future studies would improve the generalizability of the results across diverse socio-demographic contexts in Chile. This study also specifically targeted dual-earner parents with adolescents aged 10 to 15 years; therefore, future research should encompass families at various life stages to evaluate if these patterns hold across different family structures.

Moreover, although data were collected via online questionnaires, all measures were self-reported. This methodology introduces the potential for social desirability bias, in which participants might overestimate both their perceived instrumental family support and their overall diet quality to align with perceived social norms or project a favorable image of household functioning. Another limitation concerns the AHEI-derived measure: while it is helpful for assessing overall dietary patterns and frequency of habits, it does not capture portion sizes or the precise quantities consumed within each food group. This constraint limits the precise quantification of caloric and nutrient intake, which may hinder a comprehensive assessment of individual nutritional status and obscure finer variations in actual dietary exposure. Furthermore, although each parent’s perceived instrumental family support was assessed using a validated scale, a primary limitation of this instrument is that it does not distinguish the specific sources of this support—whether it originates from the partner, adolescent children, or extended family members. This distinction is particularly noteworthy given the cultural context of this research, where mothers traditionally handle most domestic duties [46]. To better untangle household dynamics, future research should refine the instrument’s administration—for instance, by adapting prompt instructions to explicitly direct participants to consider support from specific sources (e.g., partner and children)—or include items that precisely identify who provides support and estimate the proportion of the domestic workload being shared. This is a critical consideration, as our findings revealed that fathers reported significantly higher levels of instrumental support compared to mothers. This observation may reflect the “marital support gap hypothesis,” where wives generally provide more support to their husbands than they receive in return [47]. Finally, because cultural and economic factors actively shape how household responsibilities are shared, there is a clear need for cross-cultural studies in this area, particularly to compare societies with more traditional gender roles with those that exhibit greater gender equality.

### 4.4. Strengths, Novelty, and Practical Applicability

Despite its limitations, this study presents key methodological and theoretical strengths. The novelty lies in being the first investigation to examine the direct and indirect crossover effects of parents’ instrumental family support on diet quality and SWFoL within a triadic framework involving dual-earner parents and adolescents. Methodologically, analyzing matched mother–father–adolescent triads via SEM-APIMeM accounts for dyadic interdependence, providing a realistic representation of household resource allocation. Furthermore, a major strength of this study is its large, matched sample of 847 triads (2541 total participants), which enhances the statistical power of the structural model and provides robust empirical evidence on triadic family dynamics.

Practically, our results offer actionable insights for organizational policies and family-based interventions aimed at fostering healthy adolescent development. To directly support parental instrumental resources, organizational policies should extend beyond general work–life balance to implement measures that relieve practical domestic strain. Companies can provide flexible work arrangements, manageable workloads, and predictable schedules that specifically enable working parents to coordinate and execute essential household food tasks, such as grocery shopping and meal preparation. By offering mechanisms to mitigate the time strain on dual-earner parents, corporate social responsibility directly supports households’ capacity to maintain high-quality dietary habits, such as regular homemade meal preparation.

Additionally, community and workplace wellness programs should design workshops aimed at deconstructing traditional gender roles—specifically encouraging men’s involvement in food planning and enhancing their culinary skills to foster a more equitable division of instrumental family support. Finally, at the household level, interventions should focus on fostering collaborative meal routines. Enabling adolescents to participate in grocery shopping and meal preparation not only alleviates parental burden but also serves as an essential learning environment that supports food autonomy and long-term nutritional health during a critical stage of growth.

## 5. Conclusions

Our findings indicate that both mothers’ and fathers’ instrumental family support are positively associated with their own satisfaction with food-related life and that of their adolescent children. These relationships operate through direct and indirect pathways via diet quality across all triad members. Notable gender variations emerged in pathway strength: mothers tend to use instrumental support to collectivize benefits for the entire household, whereas fathers channel this support more individually to benefit themselves and their children, reflecting traditional gender roles.

From a developmental perspective, these results underscore that adolescent nutritional health is deeply embedded within the parental resource ecosystem. Tangible, task-oriented assistance within the home coexists with a positive resource framework that protects the family’s food environment during critical stages of youth growth. Interventions promoting youth nutrition should therefore extend beyond individual dietary guidelines to adopt a systemic family approach that encourages shared domestic workloads.

Theoretically, these results suggest that gain spirals within the COR framework operate via both intraindividual pathways (actor effects) and interindividual dynamics (crossover effects between partners and toward children). Deploying tangible resources in the domestic sphere simultaneously is congruent with both the nutritional and psychological ecosystems of the entire family unit.

Future research should integrate both emotional and instrumental support to compare their respective effects, specify support providers (partners vs. children), and analyze gender interactions. Finally, exploring potential moderators—such as family SES, exact adolescent age, parental work hours, fathers’ employment type, and family supper frequency—remains a valuable avenue to expand family nutrition research.

## Figures and Tables

**Table 1:** Sample characteristics (*n* = 847).

Characteristic	Total Sample
Age [Mean (*SD*)]	
Mother	38.5 (6.6)
Father	41.0 (8.2)
Adolescent	12.7 (1.8)
Adolescents’ gender (%)	
Male	425 (50.2%)
Female	422 (49.8%)
Number of family members [Mean (*SD*)]	4.2 (1.0)
Number of children [Mean (*SD*)]	2.1 (0.9)
Socioeconomic status (%)	
High	15 (1.8%)
Middle	642 (75.8%)
Low	190 (22.4%)
Number of days/week families ate together [Mean (*SD*)]	
Breakfast	3.8 (2.1)
Lunch	3.5 (2.1)
Supper	5.5 (2.2)
Dinner	4.5 (2.6)
Number of days families eat different types of foods [Mean (*SD*)]	
Homemade foods	6.3 (1.3)
Buy ready-to eat food	1.4 (1.2)
Order food at home	1.2 (0.6)
Eat at restaurants	1.1 (0.5)
Eat at fast-food outlets	1.1 (0.6)
Number of hours per day spent cooking during the week	
Mothers	2.4 (1.6)
Fathers	1.6 (1.9)
Weekly working hours	
Mothers	33.8 (14.1)
Fathers	42.3 (15.2)
Mothers’ type of employment (%)	
Woman employee	595 (70.2%)
Woman self-employed	252 (29.8%)
Fathers’ type of employment (%)	
Man employee	636 (75.1%)
Man self-employed	211 (24.9%)
Mothers’ place of work (%)	
In-person	722 (85.2%)
Remote	29 (3.4%)
Mixed	96 (11.3%)
Fathers’ place of work (%)	
In-person	773 (91.3%)
Remote	8 (0.9%)
Mixed	847 (7.8%)

**Table 2:** Summary statistics and correlations for instrumental family support (IFS) provided by parents and the dietary quality of three family members (assessed using the Adapted Healthy Eating Index, AHEI) alongside Satisfaction with Food-related Life (SWFoL) among dual-earner parents and their adolescent children.

	M (SD)	Correlations							
		1	2	3	4	5	6	7	8
1. Mothers’ IFS	14.09 (3.66)	-	0.283 ***	0.203 ***	0.154 ***	0.204 ***	0.324 ***	0.244 ***	0.183 ***
2. Fathers’ IFS	15.94 (2.97)		1	0.120 ***	0.203 ***	0.161 ***	0.189 ***	0.243 ***	0.211 ***
3. Mothers’ AHEI	62.07 (12.93)			1	0.465 ***	0.530 ***	0.327 ***	0.212 ***	0.181 ***
4. Fathers’ AHEI	57.80 (13.24)				1	0.379 ***	0.224 ***	0.302 ***	0.181 ***
5. Adolescents’ AHEI	62.06 (13.27)					1	0.218 ***	0.189 ***	0.274 ***
6. Mothers’ SWFoL	22.69 (4.40)						1	0.417 ***	0.351 ***
7. Fathers’ SWFoL	22.80 (4.47)							1	0.372 ***
8. Adolescents’ SWFoL	23.77 (4.44)								1

*** *p* < 0.001.

**Table 3:** Bias-corrected confidence intervals of specific mediation effects of the three family members’ diet quality (measured by the Adapted Healthy Eating Index, AHEI).

Specific Indirect Effects	Estimate	Lower 2.5%	Upper 2.5%	*p*-Value
Mothers’ instrumental family support → Mothers’ AHEI → Mothers’ SWFoL	0.039	0.020	0.068	0.001 **
Mothers’ instrumental family support → Fathers’ AHEI → Mothers’ SWFoL	0.006	0.000	0.016	0.182
Mothers’ instrumental family support → Adolescents’ AHEI → Mothers’ SWFoL	0.000	−0.016	0.012	0.945
Fathers’ instrumental family support → Mothers’ AHEI → Mothers’ SWFoL	0.015	−0.002	0.036	0.152
Fathers’ instrumental family support → Fathers’ AHEI → Mothers’ SWFoL	0.014	0.000	0.033	0.084
Fathers’ instrumental family support → Adolescents’ AHEI → Mothers’ SWFoL	0.000	−0.012	0.008	0.949
Mothers’ instrumental family support → Mothers’ AHEI → Fathers’ SWFoL	0.007	−0.005	0.024	0.318
Mothers’ instrumental family support → Fathers’ AHEI → Fathers’ SWFoL	0.018	0.002	0.036	0.049 *
Mothers’ instrumental family support → Adolescents’ AHEI → Fathers’ SWFoL	0.003	−0.010	0.018	0.635
Fathers’ instrumental family support → Mothers’ AHEI → Fathers’ SWFoL	0.003	−0.001	0.014	0.442
Fathers’ instrumental family support → Fathers’ AHEI → Fathers’ SWFoL	0.045	0.025	0.073	<0.001 ***
Fathers’ instrumental family support → Adolescents’ AHEI → Fathers’ SWFoL	0.002	−0.005	0.013	0.661
Mothers’ instrumental family support → Mothers’ AHEI → Adolescents’ SWFoL	0.003	−0.011	0.019	0.654
Mothers’ instrumental family support → Fathers’ AHEI → Adolescents’ SWFoL	0.003	−0.003	0.014	0.429
Mothers’ instrumental family support → Adolescents’ AHEI → Adolescents’ SWFoL	0.032	0.015	0.061	0.005 **
Fathers’ instrumental family support → Mothers’ AHEI → Adolescents’ SWFoL	0.001	−0.003	0.014	0.722
Fathers’ instrumental family support → Fathers’ AHEI → Adolescents’ SWFoL	0.008	−0.007	0.027	0.355
Fathers’ instrumental family support → Adolescents’ AHEI → Adolescents’ SWFoL	0.020	0.005	0.039	0.021 *

SWFoL: Satisfaction with food-related life. * *p* < 0.05, ** *p* < 0.01, *** *p* < 0.001.

## Data Availability

The original contributions presented in this study are included in the article/Supplementary Materials. Further inquiries can be directed to the corresponding author.

## Supplementary Materials

The following supporting information can be downloaded at: https://www.mdpi.com/article/10.3390/nu18162646/s1.

## Abbreviations

The following abbreviations are used in this manuscript:

AHEI	Adapted Healthy Eating Index
APIM	Actor–Partner Interdependence Model
CFA	Confirmatory Factor Analysis
CFI	Comparative Fit Index
COR	Conservation of Resources
RMSEA	Root Mean Square Error of Approximation
SEM	Structural Equation Modeling
SRMR	Standardized Root Mean Square Residual
SWFoL	Satisfaction with Food-related Life
TLI	Tucker–Lewis Index
