# Supplementary material for: Parents’ Instrumental Family Support, Diet Quality, and Satisfaction with Food-Related Life: A Dyadic Approach in Dual-Earner Parents and Their Adolescent Children
Source: Nutrients. 2026 Aug 13;18(16):2646. doi: 10.3390/nu18162646 (PMC13516089; doi:10.3390/nu18162646)
Supplement: Supplementary file 1 [file nutrients-18-02646-s001.zip › nutrients-4468884-SI.pdf]

ID	M_SWFoL1	M_SWFoL2	M_SWFoL3	M_SWFoL4	M_SWFoL5	M_InvApFa	M_InvApFa	M_InvApFa	M_InvApFa
2	5	5	5	5	5	4	4	4	4
3	6	5	5	5	5	4	4	3	4
11	6	5	4	5	5	5	5	5	5
12	5	4	4	4	5	4	4	3	4
14	4	4	4	4	4	3	3	3	3
16	6	5	5	5	5	5	5	4	4
17	6	4	4	4	6	5	5	5	5
19	6	4	5	4	4	5	5	4	5
20	1	3	6	6	6	4	5	5	5
21	6	6	5	6	5	3	2	4	2
22	5	3	2	3	4	3	3	4	3
23	6	5	5	5	5	5	5	5	5
25	6	5	3	4	4	3	4	4	4
29	6	6	6	6	6	5	5	5	5
30	6	5	5	5	4	4	3	4	4
31	5	4	4	5	4	2	3	3	4
32	5	5	5	4	5	4	5	5	4
33	6	4	4	1	5	2	4	3	4
37	6	1	5	5	5	4	4	5	4
40	5	5	5	4	4	2	1	3	2
41	6	4	5	4	4	3	3	3	3
45	4	4	5	5	4	3	3	3	3
47	5	5	4	4	4	3	4	4	4
48	5	4	4	4	4	3	2	2	2
49	5	4	5	5	5	3	3	3	3
50	6	6	6	6	5	4	4	4	4
51	2	4	1	1	3	3	4	5	3
52	6	4	4	4	5	4	5	5	4
53	5	5	4	4	5	3	3	3	3
55	6	6	6	6	6	4	4	5	5
56	6	5	5	5	4	5	5	5	5
57	6	4	4	4	3	4	4	3	3
59	4	5	4	4	5	3	2	2	2
63	4	4	4	4	5	3	3	3	3
65	6	5	6	5	5	5	5	5	5
68	5	3	3	3	3	2	3	2	2
71	3	4	2	2	4	2	2	3	3
73	5	4	5	4	5	3	2	4	3
74	6	5	5	5	5	3	3	4	3
77	1	2	1	1	1	2	3	4	2
80	4	5	3	3	3	2	2	3	2
84	4	4	4	3	4	3	3	3	3
86	1	1	1	1	1	4	4	4	4
89	5	4	5	4	4	4	5	5	4
90	5	4	3	2	3	2	4	1	2
92	5	5	4	4	4	3	3	4	3
93	6	5	5	4	5	5	3	5	5
94	6	6	4	4	6	4	4	5	4
96	5	4	4	4	5	3	3	4	3

97	5	5	5	5	5	3	3	4	3
100	6	6	5	5	5	4	3	4	4
101	5	3	3	3	5	4	4	4	3
106	5	5	5	5	5	4	4	4	4
107	6	6	5	5	5	4	5	3	4
108	6	5	5	5	5	4	4	5	4
110	6	4	4	4	6	5	5	5	4
111	6	4	4	4	3	4	4	4	3
112	6	6	5	5	5	5	5	5	5
113	5	4	4	4	4	3	2	4	2
114	6	5	4	4	4	3	3	3	3
116	6	4	1	3	3	2	2	3	1
117	6	4	6	5	5	4	4	4	5
119	5	4	4	4	4	4	4	4	4
121	5	5	5	5	5	3	4	4	4
124	6	6	5	6	5	4	4	5	5
127	6	5	4	4	6	3	3	3	3
129	4	5	5	5	4	4	4	5	4
130	5	5	5	5	5	4	4	4	4
132	4	4	3	4	4	3	3	3	3
136	4	4	4	4	4	4	4	4	4
141	6	4	4	3	6	4	5	5	5
143	6	3	2	3	5	3	4	4	3
144	6	4	4	3	4	4	3	4	4
145	5	4	4	3	5	3	4	3	4
148	4	4	4	5	4	4	4	4	4
149	5	5	5	3	5	3	2	3	2
151	5	5	4	4	5	4	4	3	4
152	5	5	3	3	5	4	4	4	5
153	4	3	2	3	5	4	4	4	4
155	6	5	5	5	5	5	5	5	5
158	6	1	6	5	6	5	5	5	5
159	6	6	6	6	6	5	5	5	5
161	6	5	5	5	5	4	4	5	5
163	6	3	3	2	4	4	4	4	3
164	5	5	5	5	5	4	4	4	4
165	5	5	4	4	5	5	5	5	5
168	5	5	4	5	5	2	4	5	4
169	5	2	5	5	5	3	3	3	3
170	3	4	3	4	3	2	4	4	3
172	5	3	2	2	2	3	4	3	3
181	4	4	5	2	4	2	3	3	3
182	6	4	4	5	5	4	4	4	4
184	5	5	5	5	5	3	3	3	3
185	5	5	5	4	5	3	3	4	4
186	6	4	3	2	5	3	3	3	3
189	4	3	3	3	3	2	2	2	2
190	5	3	2	2	3	4	4	4	4
191	5	3	2	4	5	5	4	4	4
195	6	5	4	4	6	4	3	4	4

196	2	2	2	2	2	5	5	5	5
199	6	6	4	4	5	3	4	4	3
200	5	4	4	4	5	4	4	4	4
201	5	5	5	5	5	3	4	2	4
202	6	4	4	4	5	5	5	5	5
204	6	6	6	6	6	4	3	3	4
205	5	5	4	3	3	3	4	3	5
208	3	3	3	3	3	3	3	3	3
210	4	4	4	4	4	3	4	3	3
211	5	5	4	5	5	4	4	4	4
214	6	6	5	5	4	3	3	3	3
218	5	4	4	4	5	4	4	3	4
223	4	4	4	4	4	2	3	3	3
231	6	5	5	5	5	4	4	3	4
233	5	5	6	4	6	4	5	5	3
234	5	5	5	5	5	4	4	4	4
241	4	4	4	4	4	4	3	4	4
242	4	4	2	2	2	2	1	3	2
243	6	3	5	5	4	5	5	5	5
245	5	5	5	4	4	3	4	3	3
246	3	5	3	4	4	3	3	3	3
247	4	3	3	3	2	2	3	3	3
248	6	5	5	6	5	4	4	4	4
249	6	6	6	5	5	4	3	5	5
261	5	5	5	5	5	4	4	4	4
266	6	4	3	3	6	5	5	5	5
268	6	3	6	5	5	3	5	5	3
269	5	5	5	5	5	3	4	5	3
282	6	6	6	6	6	5	5	5	5
283	6	5	5	5	5	5	5	5	5
285	6	3	4	3	4	4	2	4	4
290	4	4	4	4	4	4	3	3	2
296	3	3	4	4	4	3	3	3	3
300	5	4	4	4	5	4	5	5	4
301	5	5	5	5	5	4	5	4	4
302	6	6	5	5	4	2	3	3	2
303	6	6	6	6	6	2	3	3	3
304	6	3	4	4	5	2	2	5	2
306	4	4	4	4	5	2	3	3	3
308	5	6	5	5	6	5	5	5	5
309	5	5	5	4	4	2	2	3	2
320	6	6	6	6	6	2	1	1	1
323	4	3	3	3	4	3	3	4	3
324	5	3	4	2	4	3	3	3	4
325	5	4	5	5	5	3	3	4	3
332	6	3	1	1	3	5	3	5	3
333	4	3	4	4	4	3	4	4	4
334	6	4	3	4	5	3	2	4	4
335	5	4	4	4	3	3	3	4	4
338	6	3	5	4	5	2	3	4	2

339	5	2	5	4	5	3	1	4	3
340	6	6	6	6	6	5	5	5	5
347	5	4	4	3	4	2	4	4	4
349	6	6	6	6	6	1	5	1	1
350	6	1	4	6	4	5	3	5	5
351	6	4	4	4	5	5	5	5	5
352	6	4	4	4	4	5	4	3	3
353	6	4	4	4	6	4	4	3	4
354	5	5	5	5	5	5	5	5	5
356	5	4	5	5	6	3	4	5	4
357	6	5	4	5	5	4	4	4	4
358	6	3	2	2	6	4	4	3	3
359	3	4	4	4	4	5	2	4	4
363	6	4	2	2	5	2	2	5	2
365	6	5	6	5	5	4	3	4	5
366	6	5	5	4	4	3	3	4	4
367	6	6	6	6	6	5	5	5	5
368	5	5	5	5	5	4	4	4	4
369	6	5	5	4	5	4	4	4	4
370	5	5	4	4	5	4	4	5	4
375	6	6	6	5	5	2	2	4	3
380	5	5	5	4	4	4	5	4	4
400	5	5	5	4	5	4	4	4	4
401	4	3	5	5	5	2	2	3	2
402	5	3	2	3	3	2	2	2	2
403	4	4	4	4	3	3	3	3	3
405	5	4	5	3	5	3	3	4	3
407	6	5	5	5	5	3	3	3	3
408	6	4	4	4	4	5	4	3	5
409	5	4	4	3	4	2	3	3	2
410	5	4	4	4	5	3	3	3	3
412	4	5	5	6	5	3	3	3	3
413	4	4	4	4	4	3	3	3	3
414	6	5	5	5	5	4	3	3	3
415	5	5	5	5	5	3	3	3	4
416	6	6	6	6	6	4	4	4	4
425	6	5	5	5	5	3	4	3	4
431	4	3	4	3	3	3	2	2	4
432	5	1	4	4	4	4	4	4	3
434	5	6	5	5	5	3	3	3	3
435	6	6	4	5	5	4	2	5	3
437	5	4	3	3	4	2	2	2	2
439	6	5	5	5	6	4	4	4	4
440	4	3	3	3	4	3	2	3	2
441	1	2	1	2	1	3	3	3	3
442	6	3	3	3	6	4	5	5	5
443	5	4	4	4	4	3	2	3	3
446	6	4	5	4	5	3	3	3	3
451	5	5	5	5	5	3	3	3	3
452	5	3	5	5	4	5	5	5	5

453	6	4	5	5	5	4	4	4	3
454	6	5	6	5	6	3	5	3	4
455	6	5	4	4	6	4	5	5	5
463	1	2	3	3	3	4	4	4	4
468	6	6	6	6	6	4	5	3	3
471	6	3	3	4	5	3	3	3	2
473	5	2	4	4	4	4	4	4	4
481	6	6	5	5	5	5	3	5	5
482	3	3	6	3	3	3	4	4	5
483	6	5	5	5	5	5	5	5	5
484	4	4	4	4	4	3	3	3	3
492	5	5	5	4	5	4	4	5	4
494	6	5	5	4	5	5	5	5	5
495	5	5	5	5	5	4	4	5	4
496	5	5	5	5	5	4	4	4	4
497	4	5	2	2	2	2	2	2	2
498	6	5	3	4	6	3	4	4	4
499	6	5	6	5	5	5	5	4	4
504	6	5	4	4	4	4	4	4	4
505	6	6	5	4	6	4	4	5	5
506	5	5	5	5	5	3	2	3	3
509	6	6	5	5	5	4	4	5	4
511	4	4	4	4	4	4	4	4	4
512	5	4	4	4	4	5	5	5	4
520	4	4	4	4	2	3	3	3	3
521	5	4	4	5	5	3	4	5	4
522	6	6	6	6	6	4	4	5	5
526	6	6	6	6	6	3	5	5	5
531	3	6	1	1	2	3	5	1	3
532	5	5	4	2	5	4	3	4	5
534	3	5	6	4	4	2	4	3	4
536	6	2	2	2	6	4	4	4	4
537	5	3	4	3	4	3	4	4	3
539	4	5	5	5	5	3	3	3	3
541	5	3	4	3	6	5	4	4	4
542	6	5	5	5	5	3	2	3	4
543	6	6	6	5	6	4	3	5	4
544	6	4	4	4	5	3	2	3	2
545	5	4	4	4	4	5	4	4	4
546	5	5	5	5	5	5	5	5	5
548	6	1	5	5	6	3	4	4	4
554	6	6	4	5	5	5	3	5	5
555	6	5	4	4	5	3	4	4	4
556	5	3	4	5	5	3	4	4	4
558	6	6	6	6	6	5	5	5	5
560	6	2	2	2	3	1	1	1	1
563	6	6	6	5	5	3	4	4	5
565	6	4	4	4	5	5	5	5	4
573	6	6	6	6	6	4	4	4	4
575	6	4	4	4	5	3	3	4	3

576	5	5	5	5	5	3	5	4	4
580	5	5	3	3	4	3	3	3	2
602	5	5	5	5	5	3	4	4	4
603	1	4	5	6	6	2	4	3	4
605	5	4	3	5	5	3	4	4	4
608	5	5	5	5	5	3	3	3	4
617	5	4	4	4	4	2	2	4	2
619	6	4	4	4	4	4	4	4	4
620	6	6	6	6	6	5	5	5	5
632	6	6	6	5	5	4	4	4	4
634	6	5	4	4	5	4	4	5	4
635	5	3	4	4	4	2	2	3	2
636	6	6	6	6	6	3	3	4	4
638	6	6	6	6	6	4	4	4	4
639	6	6	5	6	6	5	5	5	5
641	6	4	4	4	1	5	5	5	5
642	5	3	4	4	1	3	4	4	3
649	6	5	5	5	5	5	5	5	5
705	5	4	4	4	3	5	3	3	4
707	5	4	4	4	4	3	3	3	3
709	5	5	4	4	2	1	1	1	3
710	4	3	1	2	4	2	2	4	3
711	5	6	5	5	6	3	1	1	2
712	5	5	4	4	4	2	4	4	4
713	6	5	5	4	5	3	3	3	3
714	5	3	5	2	5	3	3	3	2
717	4	3	4	4	4	3	3	3	3
718	6	6	6	6	6	3	3	3	2
719	6	4	4	5	6	3	4	4	4
750	5	5	5	5	5	4	3	2	3
751	6	4	5	4	6	4	4	3	4
752	6	5	4	3	5	4	5	5	3
753	6	3	3	4	4	3	3	4	2
754	4	3	4	3	4	4	4	4	4
755	5	6	5	5	6	4	4	4	4
757	5	5	5	5	5	5	5	4	5
758	6	6	5	5	5	4	4	5	4
759	5	5	6	6	6	3	5	5	5
762	6	5	5	5	5	4	4	4	4
764	6	3	3	2	3	3	2	2	2
765	6	2	3	1	6	4	5	5	4
767	6	6	5	5	5	3	3	4	2
800	4	4	4	3	4	2	1	4	4
810	5	5	5	5	5	4	4	4	4
813	4	3	4	4	3	3	3	3	3
814	5	3	3	3	3	4	4	5	4
817	5	4	3	3	3	3	3	4	2
818	6	4	3	3	6	2	2	4	3
822	5	5	5	4	4	4	4	4	4
826	4	6	4	4	4	3	2	2	2

827	6	4	4	4	4	2	3	3	3
828	6	5	4	5	6	3	3	3	2
829	6	6	6	6	6	4	4	4	4
830	5	4	3	4	4	3	4	4	3
831	5	4	4	4	5	4	4	4	4
832	4	4	4	4	4	3	3	3	3
836	4	3	2	2	4	2	2	4	2
837	5	5	4	4	4	3	3	4	4
839	5	4	4	6	5	5	5	5	3
842	5	5	4	4	5	4	4	4	4
860	5	5	6	5	5	4	5	5	5
861	4	4	3	4	4	4	3	3	2
862	4	5	5	4	4	3	3	2	2
863	4	4	5	5	5	3	3	2	2
864	6	4	5	4	5	3	2	5	4
865	4	4	3	4	3	2	2	2	3
866	4	4	3	4	5	3	2	2	2
867	5	4	3	4	4	3	3	2	2
868	4	4	3	4	3	4	3	3	3
869	4	3	4	5	4	3	2	3	2
870	5	5	5	5	5	3	3	3	3
871	5	4	4	4	4	4	4	4	4
872	5	5	5	5	5	3	3	3	3
873	5	5	5	5	5	3	3	3	3
874	6	6	6	6	6	4	4	3	3
875	5	4	4	3	3	3	4	4	4
876	6	6	6	6	6	5	5	5	5
877	6	6	6	6	6	4	4	4	4
878	5	4	4	4	4	4	4	4	4
879	5	4	3	3	4	3	3	5	2
880	6	4	4	4	6	4	5	5	2
881	5	3	2	3	4	5	4	5	4
882	6	5	4	4	5	3	3	3	3
883	5	4	4	4	4	2	3	3	4
886	5	4	4	4	5	4	4	4	4
888	6	6	3	2	6	3	3	3	3
889	5	4	4	4	4	2	3	3	3
890	5	4	4	4	5	3	2	3	3
891	5	3	3	4	4	4	4	4	4
892	5	1	1	1	2	3	3	2	1
893	2	4	2	4	5	4	3	3	4
894	5	5	4	4	5	4	4	4	4
895	6	6	5	6	6	5	4	5	4
896	6	4	4	4	4	3	3	4	3
897	6	6	6	6	6	3	3	3	3
898	4	3	4	3	3	2	2	3	2
899	6	6	6	6	6	3	3	3	3
884	5	6	4	4	6	4	3	3	3
900	6	5	5	5	5	4	4	4	4
901	3	4	5	2	4	2	2	3	2

902	6	5	5	5	5	4	4	4	4
903	4	4	4	4	3	3	3	2	3
904	5	5	5	5	5	5	5	5	5
905	6	2	2	2	2	4	4	4	4
906	5	3	3	2	4	3	3	2	2
907	4	5	3	4	3	4	3	3	3
908	6	6	6	6	6	5	5	5	5
909	6	6	5	5	5	5	5	5	5
910	5	4	4	5	5	3	3	2	3
911	5	4	5	6	6	3	2	3	2
912	4	3	5	4	5	3	4	5	4
913	5	4	5	4	4	3	2	3	2
914	5	4	5	5	5	3	2	2	3
915	5	4	4	5	5	3	4	3	4
916	5	4	5	4	4	3	2	3	2
917	5	4	4	3	4	3	2	2	2
918	5	4	5	5	6	3	3	4	3
919	5	5	4	4	4	3	3	3	3
920	5	5	5	5	5	2	2	2	3
921	6	6	5	4	4	4	4	4	4
922	3	4	3	2	2	2	2	3	3
923	6	4	4	4	4	4	4	3	4
927	6	4	4	5	6	2	3	5	1
928	4	4	4	4	3	5	5	5	5
930	6	6	4	4	4	5	5	5	5
931	3	4	4	4	3	3	3	3	3
932	6	2	5	6	6	3	4	4	4
933	5	5	5	5	5	4	4	4	4
934	6	6	6	6	6	1	2	3	3
935	6	5	5	5	5	3	2	4	3
936	6	6	5	5	5	3	4	4	3
937	6	6	6	5	5	3	4	3	3
938	6	6	6	6	6	4	5	5	5
939	6	6	6	6	6	4	5	4	4
940	6	3	4	3	5	3	4	3	3
941	6	5	5	5	6	4	4	4	4
942	6	6	6	6	6	5	5	5	4
943	3	3	4	3	4	2	2	3	3
994	2	1	2	3	3	3	3	3	3
944	6	5	5	5	5	4	4	4	4
946	5	5	5	5	5	4	5	4	4
947	6	4	3	4	5	2	3	3	4
949	6	4	4	5	5	4	4	4	4
951	5	5	5	5	5	3	4	4	4
953	4	4	3	4	4	3	3	3	3
954	5	3	4	4	4	3	4	2	1
955	5	5	5	5	5	4	2	4	4
958	5	5	5	5	5	3	3	2	2
959	5	5	4	4	3	4	4	3	4
961	5	3	2	3	4	3	3	4	3

962	6	6	5	5	5	5	4	5	5
963	5	5	5	4	5	4	4	4	4
964	6	1	5	5	5	5	4	5	5
965	6	6	6	4	5	5	4	4	5
967	6	6	6	6	6	5	3	5	5
968	6	6	6	6	6	5	5	5	5
969	6	6	6	6	6	1	1	1	1
971	6	4	4	4	4	4	4	5	4
972	6	6	6	5	6	4	4	4	4
973	5	4	5	4	6	3	3	4	3
974	5	4	5	5	5	3	4	4	4
975	6	5	5	5	5	5	5	5	5
976	4	4	4	4	5	4	5	4	4
977	6	4	4	5	5	4	4	4	4
978	6	6	6	6	6	5	5	5	5
979	5	4	4	4	5	4	4	5	4
980	6	6	1	2	6	3	4	4	4
981	6	4	6	4	6	5	5	5	5
982	6	5	5	5	5	4	5	5	5
984	3	3	3	3	2	2	2	3	3
986	5	1	6	5	4	5	5	5	5
987	5	2	3	2	5	4	4	4	3
997	2	2	2	2	2	1	1	1	1
988	5	5	5	4	5	5	5	5	5
989	6	3	2	2	4	2	3	5	3
990	5	5	5	5	5	2	2	2	2
991	6	6	6	6	5	5	5	5	5
992	6	5	5	3	5	5	5	5	5
993	6	5	5	4	4	3	4	4	4
995	6	6	6	6	6	3	3	4	3
996	6	3	1	3	6	1	1	3	1
1	5	4	3	4	5	3	3	3	2
2	6	5	5	4	5	3	4	4	2
3	6	4	4	3	4	4	4	4	4
5	5	5	1	3	5	4	3	4	3
6	5	5	5	4	4	1	2	3	2
7	5	5	5	4	5	4	4	4	4
8	6	4	4	4	5	4	5	5	4
9	6	5	4	5	5	4	3	4	3
10	5	5	5	5	5	3	4	4	4
11	5	4	2	3	5	3	3	3	3
12	6	4	4	4	6	3	1	3	3
13	6	3	3	3	6	4	4	5	5
14	6	5	5	4	4	3	3	4	3
15	5	5	5	5	5	4	4	4	4
16	5	3	3	4	4	3	3	3	3
17	6	4	4	4	5	4	2	4	3
18	5	5	4	5	4	3	3	3	4
19	6	4	4	4	5	4	4	5	4
20	5	5	5	4	5	2	2	4	4

21	4	4	4	4	4	2	3	4	2
22	5	5	5	3	3	4	5	5	5
23	5	4	4	4	4	4	4	3	3
24	5	5	5	5	5	4	3	4	4
25	5	3	3	3	4	3	1	3	2
27	6	5	4	4	5	4	4	4	4
28	6	5	5	5	5	4	4	4	4
29	6	3	4	2	5	3	2	3	3
30	6	5	5	5	5	2	2	4	2
31	6	4	4	3	5	3	5	3	3
32	6	3	3	3	3	3	2	3	3
34	5	3	3	3	4	3	4	5	3
36	5	4	4	4	5	4	4	4	4
37	5	4	4	4	4	4	4	4	4
38	5	5	5	5	5	3	4	4	4
39	3	3	2	1	4	2	3	5	5
41	6	6	6	5	5	4	2	4	4
42	6	6	1	2	6	3	2	4	1
43	6	1	5	5	5	5	5	5	5
44	6	1	4	4	6	5	5	5	5
45	5	5	4	4	5	2	2	1	2
46	4	5	2	2	2	5	5	5	5
47	4	4	3	2	2	4	4	4	4
48	6	3	3	3	6	3	4	5	5
49	5	5	5	4	4	3	3	4	3
50	6	1	5	5	4	5	3	3	5
51	5	4	4	4	4	2	2	4	2
52	2	2	3	3	3	3	3	2	3
53	5	5	3	4	5	1	1	5	1
54	2	2	2	1	3	3	3	4	4
55	6	4	4	4	4	4	5	4	3
56	5	4	4	4	4	4	4	3	3
57	6	6	6	6	6	5	5	5	5
58	6	5	5	5	6	4	4	3	3
59	6	6	5	5	5	4	3	4	4
60	3	3	3	4	4	3	3	3	3
61	5	4	4	4	5	3	2	4	2
62	6	6	3	3	4	5	5	3	4
63	6	2	4	4	5	3	4	5	3
64	6	6	6	6	6	3	2	1	2
65	6	5	5	5	5	5	5	5	5
66	6	6	3	4	6	5	5	5	5
67	5	4	4	3	3	2	4	5	4
68	5	5	4	3	5	3	4	4	4
69	6	6	6	6	6	3	5	5	4
70	5	5	4	4	4	5	5	5	5
71	5	5	4	4	3	4	4	4	4
72	6	6	5	4	5	4	5	5	5
73	3	4	4	4	5	3	3	4	3
74	4	5	4	5	4	4	4	5	4

75	6	5	5	5	5	4	4	4	4
77	5	4	3	3	5	4	4	4	4
78	6	6	6	6	6	5	5	5	5
79	6	6	6	5	5	4	4	5	5
80	4	2	1	1	1	3	4	4	4
81	6	5	4	4	6	5	5	4	4
82	5	4	3	2	4	4	3	3	4
83	6	4	4	4	5	3	4	4	4
84	5	5	5	5	6	4	3	5	4
85	4	4	4	2	4	2	2	3	2
86	6	6	5	6	6	5	5	5	5
87	6	5	4	4	5	4	5	5	5
88	5	5	5	5	5	4	4	4	4
89	6	6	6	5	5	4	3	3	4
90	6	6	6	6	6	3	4	4	3
91	5	4	4	2	3	3	3	4	3
92	6	5	5	5	5	2	4	5	2
93	5	4	3	3	5	2	2	3	2
94	6	4	4	4	5	5	3	5	5
95	6	5	5	4	5	3	4	4	3
96	6	6	6	6	6	4	5	5	5
97	6	6	5	5	5	2	2	4	4
98	5	5	5	5	5	3	3	3	3
99	6	5	4	4	3	4	4	4	4
100	5	3	3	4	4	4	5	4	3
101	6	5	4	4	5	3	2	2	4
102	5	5	5	5	5	3	4	3	3
103	6	6	6	6	6	5	5	5	5
105	6	5	5	5	6	4	5	5	4
106	6	5	3	4	5	2	3	4	3
107	5	5	4	4	4	5	5	5	3
108	5	4	5	4	6	3	2	4	4
109	6	1	4	3	4	3	3	2	1
110	4	4	4	4	4	3	4	3	3
111	4	2	2	2	2	3	4	4	3
112	6	5	4	4	5	4	5	5	5
113	5	5	5	5	5	4	4	4	4
114	5	6	5	5	5	4	4	5	4
115	5	3	2	2	2	5	5	5	5
116	6	5	5	5	6	5	5	5	5
117	4	3	4	3	5	2	2	2	2
118	6	6	6	6	6	2	4	5	2
119	6	4	4	4	4	4	4	4	2
120	4	3	3	3	3	2	2	4	2
121	6	5	4	4	4	3	4	3	3
122	5	5	5	5	5	4	4	4	4
123	6	5	5	5	5	3	2	4	3
124	6	4	5	3	3	3	4	4	3
125	6	6	5	6	6	4	4	5	4
126	5	5	5	5	5	3	4	4	4

127	6	6	6	6	6	5	5	5	5
128	5	4	4	3	4	4	4	4	3
129	6	4	5	5	5	4	5	4	5
131	5	3	5	4	5	3	3	4	2
132	5	2	4	4	5	4	4	4	4
133	5	5	3	4	4	3	4	4	3
134	6	6	6	6	6	4	4	4	3
135	6	6	5	5	6	5	4	5	5
136	5	2	5	3	5	4	4	4	4
137	6	4	4	4	4	5	5	5	5
138	4	5	4	4	4	4	4	5	4
139	6	2	2	5	6	3	5	5	2
140	3	3	4	4	4	2	3	3	3
141	6	5	5	5	5	4	4	4	4
142	5	3	2	2	3	3	1	2	2
143	6	4	4	4	6	4	5	5	5
144	5	5	4	4	5	2	3	4	3
145	5	3	5	5	4	3	4	4	4
146	6	4	3	3	4	4	4	4	4
147	6	5	4	5	6	5	5	5	5
148	4	4	3	2	5	4	4	4	4
149	5	5	4	4	4	4	3	2	3
150	6	5	4	4	4	5	5	5	5
151	6	6	6	6	6	5	5	5	5
152	2	3	2	4	3	2	3	2	3
153	5	5	4	4	5	4	4	2	4
154	5	3	3	3	4	2	2	4	2
155	5	3	5	4	5	1	1	2	2
156	3	5	3	4	3	2	4	2	2
157	6	1	5	5	6	2	3	3	3
158	1	1	1	2	2	4	5	4	4
159	5	5	5	5	5	2	2	3	2
160	5	3	5	5	5	4	4	4	5
161	6	5	5	5	5	4	4	4	4
162	4	5	4	5	5	3	3	4	3
163	6	6	4	3	6	3	3	3	3
164	6	5	5	5	6	3	3	4	3
165	5	5	5	5	5	4	4	4	4
166	2	5	5	4	4	2	4	2	2
167	6	5	4	5	4	3	4	3	4
168	4	4	4	4	4	3	3	3	4
170	5	5	4	4	4	3	4	4	3
172	6	5	5	5	6	4	4	5	4
173	6	6	5	4	4	3	4	3	4
174	6	5	5	5	5	4	4	4	4
175	6	6	6	6	6	5	4	5	4
176	6	1	5	5	5	4	5	5	4
177	6	6	6	6	6	4	5	4	4
179	5	5	4	4	5	4	3	3	4
180	2	5	5	4	4	5	4	4	5

181	6	4	4	4	5	4	5	5	4
182	6	6	6	6	6	5	5	5	5
183	6	5	5	4	6	4	5	5	5
184	4	2	2	2	4	2	2	3	2
185	5	5	3	5	4	3	3	5	5
186	5	5	4	3	5	3	3	3	3
187	5	5	5	4	5	4	5	5	4
188	5	4	4	4	4	5	4	4	5
189	6	6	5	4	5	2	3	4	3
190	5	3	3	3	3	3	5	3	3
191	5	4	2	3	4	2	4	4	4
192	5	4	4	4	4	4	4	4	4
193	5	4	4	4	4	4	4	4	5
194	1	3	2	2	3	1	1	1	2
195	5	4	4	3	5	4	4	3	3
196	5	5	4	3	5	4	4	4	4
197	5	5	5	5	5	2	3	4	2
198	5	5	4	5	5	2	2	3	2
199	6	5	5	5	5	3	4	2	3
200	6	2	4	4	4	3	4	4	4
201	6	5	4	4	5	4	4	4	4
206	6	5	5	4	6	3	4	4	3
208	6	5	5	5	5	4	5	5	5
209	5	3	4	2	4	4	2	3	4
211	6	6	6	6	6	3	3	3	3
212	6	5	5	4	6	3	3	3	3
213	6	6	5	5	6	4	4	4	4
214	6	6	6	6	6	3	2	3	3
215	4	4	4	4	4	3	3	3	3
216	5	5	5	5	5	4	4	4	4
217	4	4	4	4	4	3	4	2	2
218	5	4	5	5	6	4	3	4	4
219	6	4	4	5	5	3	3	2	3
220	3	1	1	1	1	1	1	1	1
221	1	3	4	4	4	4	3	3	3
223	3	1	1	1	4	1	3	3	1
224	5	4	5	5	6	4	4	4	5
225	6	5	5	5	6	4	4	5	5
226	6	6	5	5	5	3	4	4	4
227	6	2	5	3	5	3	3	3	3
228	5	2	3	2	4	2	2	2	2
229	4	5	4	4	4	3	3	3	3
230	1	5	2	2	2	3	3	3	3
231	6	5	5	4	5	4	4	3	4
232	5	3	4	4	5	4	4	4	3
233	3	4	4	3	4	3	3	3	3
234	3	2	5	4	2	4	4	4	4
235	5	3	4	5	4	4	5	5	5
236	5	4	4	4	4	4	3	3	4
237	4	4	4	4	4	4	3	3	4

238	5	4	4	3	3	3	3	5	3
239	4	4	4	3	5	4	5	4	4
240	5	2	3	4	5	2	4	2	3
241	5	2	6	5	5	3	4	5	3
242	6	6	5	5	5	5	4	3	5
243	6	5	5	5	5	4	4	4	4
244	4	3	2	3	3	2	2	2	2
245	5	4	4	4	5	3	4	4	3
246	6	6	6	6	6	3	4	5	5
247	6	6	6	6	6	4	5	4	4
248	6	5	4	4	6	4	3	5	4
249	6	6	5	5	5	4	3	4	4
250	5	4	4	4	4	4	4	4	4
251	5	2	2	2	2	4	4	5	4
252	6	5	5	5	5	3	3	5	3
253	5	6	5	5	5	4	4	4	4
254	6	2	2	2	5	2	2	4	2
255	4	4	4	4	4	3	3	3	3
256	6	4	4	4	4	4	3	5	4
257	6	6	5	5	5	4	4	5	4
258	5	4	4	4	5	2	4	4	2
259	5	2	2	3	4	4	2	2	3
260	6	5	4	4	5	4	3	5	4
261	6	4	4	4	4	3	3	3	3
262	5	5	5	5	5	4	4	4	4
263	4	6	4	4	3	1	1	4	1
264	5	5	5	5	4	2	3	3	2
265	5	5	4	3	4	4	4	4	4
266	4	5	5	4	3	2	2	2	3
267	6	4	4	4	5	2	2	4	2
268	5	5	5	5	5	5	5	5	5
269	6	3	4	3	3	3	3	2	3
270	6	6	6	6	6	5	5	5	5
271	5	2	2	2	5	4	4	4	4
272	6	5	5	4	6	4	4	4	4
273	5	5	5	4	4	2	3	4	2
274	5	4	3	4	5	2	1	3	1
275	6	6	6	6	6	5	5	5	4
276	5	4	4	4	4	4	3	4	4
277	5	5	4	4	5	3	3	4	4
278	6	5	5	5	5	4	4	4	3
279	6	6	6	6	6	3	4	3	4
280	6	3	3	4	6	3	4	4	3
282	5	4	4	4	4	4	4	3	3
283	1	1	1	1	1	5	5	3	5
284	5	4	4	4	4	1	4	4	4
285	6	6	6	6	6	4	4	3	4
286	4	2	2	1	2	2	3	3	1
287	4	3	3	3	4	3	4	4	4
288	5	5	3	4	4	3	3	3	3

289	5	5	4	4	6	4	5	5	4
290	6	4	4	4	2	4	3	4	4
291	6	6	6	6	6	4	3	4	4
293	4	4	4	5	3	3	5	5	5
294	6	4	4	4	5	4	4	4	5
295	6	1	6	6	6	2	4	4	2
296	6	1	6	5	5	4	4	5	4
297	4	4	4	4	4	3	3	3	3
298	5	2	4	4	5	4	4	4	4
299	4	5	3	5	5	3	3	4	3
300	3	3	2	2	2	4	4	5	5
301	4	5	4	4	4	5	3	5	5
302	6	4	4	3	6	3	5	5	2
303	6	4	4	4	4	5	1	4	5
304	6	3	4	4	5	2	4	5	4
305	6	6	5	5	6	4	4	4	4
306	6	5	5	5	5	5	5	5	5
307	5	4	3	3	4	4	4	5	4
308	5	4	3	3	4	4	4	5	4
309	6	5	5	4	5	3	4	3	3
311	5	5	5	5	5	3	4	4	4
312	6	5	5	5	6	3	5	5	3
313	6	5	5	5	5	3	3	4	4
314	6	6	5	6	5	4	4	4	4
315	5	5	5	6	6	4	5	2	5
316	6	5	5	5	5	3	3	3	2
317	6	6	5	5	6	5	5	5	5
318	6	3	3	3	4	4	5	5	5
320	5	3	3	3	2	3	4	4	3
321	5	4	4	5	6	5	2	5	5
322	5	4	5	5	5	5	4	4	4
323	4	3	3	4	4	4	4	3	3
324	5	4	4	3	4	4	4	4	4
325	5	5	4	4	4	3	4	4	4
327	6	6	6	6	6	4	4	4	4
329	5	5	4	4	3	3	4	4	4
330	6	3	1	2	2	2	5	4	2
331	5	4	4	4	4	5	5	5	4
332	6	6	6	1	1	5	5	5	5
333	6	5	5	5	6	5	4	4	4
334	5	5	5	5	2	4	4	5	3
335	6	6	6	6	6	5	5	5	5
336	5	4	4	4	6	4	3	4	4
337	6	4	3	4	4	3	2	4	3
338	6	4	5	4	5	5	5	5	5
339	6	2	3	3	4	4	3	5	5
340	4	5	4	4	4	3	3	4	3
341	1	6	4	6	6	3	3	3	3
342	5	4	4	4	4	2	2	4	2
343	6	4	3	4	4	2	3	1	2

344	6	6	5	5	6	3	3	4	3
345	4	4	4	4	4	5	5	5	5
346	6	5	5	5	5	5	5	5	5
347	6	6	6	5	5	2	2	3	3
349	5	3	2	3	4	2	5	5	2
350	5	6	6	5	5	3	1	4	5
352	6	6	6	6	6	3	3	4	4
353	6	1	4	4	4	3	3	5	3
354	6	4	4	4	4	3	1	5	3
355	4	4	3	3	3	3	4	3	4
356	6	5	5	4	5	4	5	5	4
357	6	5	5	5	5	4	5	4	5
358	6	5	5	5	6	3	3	4	3
359	6	5	4	5	5	3	4	4	3
360	6	5	5	5	5	4	4	4	4
361	5	5	4	4	4	2	2	4	2
362	6	3	6	6	6	3	1	4	2
363	5	6	6	5	5	3	3	4	4
364	6	3	2	3	3	2	2	4	2
365	6	5	5	5	6	5	4	3	4
366	6	6	5	5	6	4	4	4	4
367	4	5	5	5	4	3	3	4	3
368	5	5	5	5	5	5	5	4	4
370	4	5	3	4	5	3	4	4	4
372	6	6	6	6	6	5	5	5	5
373	6	5	4	4	4	4	5	5	4
375	6	6	4	3	4	4	5	5	4
377	6	4	4	4	4	2	3	5	3
378	5	5	4	4	4	3	4	4	4
379	6	5	4	4	4	4	4	4	4
380	4	2	3	5	4	5	5	5	4
381	6	6	6	6	6	3	4	4	3
382	3	4	4	4	4	3	4	4	2
383	5	3	4	4	4	3	3	4	4
384	5	5	5	5	5	3	4	4	4
385	6	5	5	5	5	3	4	3	4
386	6	4	4	4	3	3	3	3	3
387	5	5	4	5	3	4	3	4	4
388	4	3	4	3	4	4	3	4	3
389	6	4	4	5	4	3	4	4	4
390	5	6	6	5	6	4	5	4	4
392	1	2	2	2	2	2	2	1	1
394	5	4	5	4	5	3	3	3	3
395	6	6	6	6	6	5	5	5	5
396	6	5	5	5	6	3	4	5	4
397	6	4	4	5	5	4	3	4	5
398	5	3	3	3	3	2	2	3	2
399	6	5	5	5	5	3	5	4	3
400	5	5	5	5	5	2	2	4	2
401	6	6	6	4	3	4	4	5	5

402	6	6	6	6	5	5	4	4	4
403	6	1	4	5	5	4	5	5	5
404	6	5	5	5	4	3	4	4	3
405	6	6	3	4	4	1	2	2	1
406	5	4	4	4	4	3	5	5	4
407	5	3	3	4	2	2	2	2	2
408	6	5	4	4	5	4	4	4	4
409	4	4	4	3	4	3	4	4	4
410	6	6	3	5	5	5	4	3	4
411	6	6	6	5	5	3	3	3	2
413	5	4	4	4	5	4	4	4	5
414	3	2	1	1	2	2	2	4	2
415	4	5	5	5	4	3	3	3	3
417	5	1	5	5	6	3	4	4	4
418	6	6	6	6	6	5	3	5	5
419	5	4	4	4	4	3	3	4	3
420	5	5	5	5	5	4	4	5	4
421	5	5	5	5	5	3	3	5	3
423	5	5	4	4	5	3	3	3	3
425	6	5	5	5	5	4	3	4	4
428	4	4	4	4	1	5	5	3	5
429	6	4	3	3	3	5	4	4	5
430	4	5	4	4	4	4	5	4	4
433	5	5	4	4	4	2	2	2	2
501	6	5	5	5	5	4	4	3	4
508	6	6	4	6	6	5	5	5	5
510	5	4	4	4	4	4	4	4	4
511	6	5	4	4	4	4	4	3	4
518	5	6	5	5	4	4	5	5	3
519	6	6	5	5	5	4	5	4	4
521	6	4	5	3	5	5	5	4	5
530	6	6	6	6	6	5	3	5	5
531	6	4	3	6	5	5	5	5	5
533	5	2	5	5	5	4	5	4	5
534	6	6	5	5	6	5	5	5	4
545	5	4	4	5	4	4	4	5	4
551	6	6	5	5	6	5	4	4	4
558	4	5	4	3	4	3	5	3	3
562	6	5	5	4	5	3	2	3	2
563	6	4	3	5	6	4	5	5	4
568	6	5	4	4	5	3	5	5	3
571	5	6	5	5	5	3	2	4	2
572	5	3	2	4	4	4	4	4	4
576	5	3	4	4	5	3	2	3	2
578	6	5	4	5	6	4	4	3	3
579	5	5	4	3	5	4	4	4	4
583	5	5	5	5	5	1	3	3	1
599	3	1	1	1	1	4	5	5	4

M_InvApFa	M_InvApFa	M_InvApFa	M_InvApFa	P_SWFoL1	P_SWFoL2	P_SWFoL3	P_SWFoL4	P_SWFoL5	P_InvApFam
4	4	4	4	3	4	4	3	3	3
4	5	4	4	2	4	4	4	4	5
5	5	5	5	6	6	6	6	6	5
3	3	3	4	6	5	5	5	5	4
3	3	3	5	6	4	4	5	5	5
4	4	4	4	5	4	4	4	4	5
5	5	5	5	6	4	4	4	6	4
4	4	4	4	5	4	4	5	4	4
5	5	5	5	6	6	6	6	6	5
1	2	1	3	6	4	3	3	5	4
3	4	4	4	4	5	4	4	4	4
5	5	5	5	6	6	6	6	6	3
4	4	4	4	5	3	4	4	3	3
5	5	5	5	5	3	5	5	5	4
4	4	4	4	6	5	5	5	5	3
3	3	3	3	4	2	3	3	2	2
4	5	5	5	5	4	4	4	5	4
4	4	5	5	3	5	4	3	4	2
5	4	5	5	6	6	5	5	5	5
1	3	3	3	1	2	3	3	2	2
3	3	3	3	5	3	4	4	3	3
3	3	3	3	5	4	4	4	4	4
4	4	4	4	4	4	4	4	4	3
2	2	2	3	4	4	4	4	4	3
3	3	3	3	4	4	5	5	5	4
4	4	4	4	4	4	5	5	5	4
3	4	5	5	5	3	1	1	3	5
4	4	4	4	6	5	5	4	6	4
3	3	4	4	5	4	5	5	5	4
4	4	4	5	6	6	6	6	6	5
4	4	4	4	6	6	6	6	5	4
3	3	3	3	5	5	5	5	5	3
3	3	3	3	6	5	5	5	6	1
3	3	3	4	5	4	4	4	4	4
5	5	5	5	5	6	6	6	6	5
2	3	3	4	3	6	6	5	5	4
2	2	3	3	4	2	2	1	6	4
3	2	3	4	3	3	4	4	5	4
2	3	4	4	6	6	4	5	5	5
3	4	4	4	2	4	4	2	2	4
1	3	3	1	4	3	3	3	3	4
3	3	3	3	4	4	3	2	3	4
4	4	4	4	6	6	6	6	6	5
4	4	4	4	1	2	2	2	4	5
5	5	4	4	4	4	4	4	4	5
4	3	5	4	5	5	4	4	5	3
5	5	5	4	5	5	5	6	4	5
2	4	5	5	5	5	5	5	5	5
3	3	3	4	5	3	3	3	4	3

3	3	4	4	6	5	5	5	5	5
3	3	3	3	5	4	4	4	5	3
3	4	4	5	5	5	4	4	5	4
4	4	4	4	5	5	5	5	5	5
4	4	4	3	5	5	5	6	5	3
4	4	4	5	4	3	3	3	3	5
4	4	4	5	6	3	3	4	5	4
4	4	4	3	4	4	3	4	3	3
5	5	5	5	6	5	4	4	5	5
4	3	4	4	4	4	4	5	5	4
3	4	4	3	6	4	4	4	4	5
2	3	4	4	6	2	1	1	4	3
4	4	4	4	2	4	5	5	3	3
4	4	4	4	6	5	5	5	5	3
4	4	4	4	5	5	5	5	5	4
4	4	4	5	6	6	5	6	6	4
3	4	4	4	3	2	4	2	2	3
4	4	4	4	4	5	5	5	5	4
4	4	4	4	5	5	5	5	5	4
3	4	3	3	4	4	4	4	4	3
4	5	4	5	4	3	5	3	4	4
5	4	4	4	6	6	4	3	5	5
3	3	4	3	5	5	5	5	4	3
3	3	4	4	6	6	5	4	5	4
4	3	3	5	5	4	4	4	4	3
4	4	4	4	4	5	5	5	4	4
2	3	2	4	1	3	5	5	5	3
3	4	4	4	5	2	4	4	5	5
5	4	4	4	6	4	4	4	5	4
4	4	4	4	3	4	3	3	4	3
5	5	5	5	4	4	4	3	3	4
5	5	5	5	6	6	6	6	6	5
5	5	5	5	6	6	6	6	6	5
4	5	5	5	6	5	5	5	5	5
4	3	4	3	5	4	4	3	5	4
4	4	4	4	5	5	5	5	5	5
5	5	5	5	6	5	4	4	6	5
4	4	4	5	6	6	5	5	5	3
3	3	3	3	5	5	5	5	6	3
3	2	2	3	4	4	4	4	3	4
2	2	2	3	2	2	4	2	5	2
2	3	3	4	3	2	5	2	3	4
1	2	3	4	6	6	5	5	5	4
3	3	3	3	6	6	6	6	6	4
4	4	4	3	4	5	4	5	5	3
3	3	3	5	4	4	4	4	3	4
2	3	3	4	6	6	5	6	6	5
4	4	4	5	5	3	2	2	3	4
5	4	5	5	6	5	5	5	5	4
4	4	4	4	6	5	5	4	6	3

5	5	5	5	5	5	5	5	5	5
3	4	4	3	5	5	5	5	5	5
4	4	4	4	5	5	5	5	5	5
4	2	2	3	4	4	4	4	5	3
5	5	5	5	6	6	6	6	6	5
3	4	4	5	6	2	2	3	2	4
2	3	3	3	4	3	4	3	3	4
3	3	3	3	3	3	3	3	3	3
4	2	4	4	4	3	3	3	3	5
3	4	4	4	5	5	5	5	5	4
3	3	3	3	6	6	5	5	5	3
4	3	3	3	6	1	1	2	4	4
3	3	3	4	5	3	3	3	4	4
3	3	3	3	6	6	6	5	5	5
3	4	4	5	6	4	4	4	5	5
4	4	4	4	6	6	6	6	6	5
4	4	4	4	5	4	5	5	5	4
1	3	3	3	5	5	2	3	3	3
5	5	5	5	4	3	3	3	3	4
5	4	4	4	2	3	3	2	3	4
3	3	3	3	4	3	4	5	3	3
3	3	3	3	4	4	4	3	3	3
4	4	4	4	6	6	6	6	6	4
4	5	4	5	6	2	5	5	5	5
4	4	4	4	3	3	3	5	4	3
5	5	5	5	6	3	5	3	6	5
5	4	4	5	6	4	5	4	5	5
3	5	5	5	4	3	4	3	4	3
5	5	5	5	6	5	6	5	5	4
5	5	5	5	5	5	5	5	5	5
4	3	2	4	6	4	3	3	4	5
2	3	4	3	4	3	4	3	3	3
3	3	3	3	3	4	3	4	3	3
4	4	5	5	6	4	4	4	5	4
4	4	4	5	5	5	5	5	5	4
3	3	3	3	4	4	4	4	4	4
3	3	3	3	6	6	6	6	6	1
2	3	3	5	6	3	3	4	6	5
5	3	3	4	5	3	4	4	5	3
5	5	5	5	6	6	4	5	5	5
2	3	3	1	6	5	5	5	5	4
1	1	1	1	6	6	6	6	6	1
3	4	4	4	4	5	4	3	4	2
4	4	4	4	6	5	5	5	5	2
4	4	4	4	4	3	5	5	5	3
4	4	4	5	6	6	6	6	6	5
3	3	4	4	5	3	3	2	3	4
2	5	4	2	6	4	4	3	4	3
3	4	4	4	5	5	4	4	4	4
3	2	4	4	5	5	4	4	4	3

3	3	3	4	4	4	4	4	4	4
5	5	5	5	6	6	6	6	6	5
3	4	4	4	5	4	1	1	5	5
1	1	1	2	5	5	4	4	5	3
3	5	5	3	6	6	5	6	6	5
5	5	5	5	6	4	4	4	4	3
4	5	5	5	5	4	4	5	5	5
1	3	4	4	4	4	4	5	5	4
5	5	5	5	5	3	3	3	3	3
5	5	5	5	5	5	5	5	5	5
4	3	3	4	6	6	5	5	5	3
3	2	3	4	5	4	2	2	5	4
5	4	5	4	2	3	1	3	1	5
2	4	4	5	6	5	4	4	6	5
4	4	5	4	6	6	6	5	6	4
4	4	4	4	6	6	5	5	5	4
5	5	5	5	6	6	6	6	6	5
4	4	4	4	6	5	5	4	5	4
4	4	4	5	5	4	5	3	5	5
2	4	4	5	4	6	5	5	5	4
4	4	4	2	5	5	5	5	5	4
4	5	4	4	6	6	6	6	6	5
1	3	4	4	6	5	5	5	5	4
1	3	3	3	5	1	5	5	4	3
2	3	3	2	6	4	4	4	4	3
3	3	5	4	4	4	4	4	4	3
3	4	4	4	5	4	4	4	4	3
3	3	3	2	5	5	5	5	5	4
3	4	4	5	6	5	5	5	6	5
2	3	3	3	5	5	5	4	5	5
2	3	3	4	5	5	5	5	5	3
3	4	3	4	5	5	4	4	4	3
3	2	2	3	4	4	3	3	3	3
3	4	3	3	4	4	4	4	4	4
3	3	3	3	4	4	4	4	4	4
4	3	3	3	3	4	4	4	4	4
3	4	4	3	5	4	4	4	4	3
4	1	4	1	6	6	6	6	4	4
2	5	4	3	5	5	4	5	5	3
2	3	3	4	5	5	5	5	5	4
1	3	3	1	6	6	5	4	4	2
2	2	2	2	5	4	3	3	3	3
4	4	4	5	6	6	6	6	6	3
2	3	3	3	4	4	4	4	5	3
3	3	3	3	3	3	3	3	3	3
5	5	5	5	5	3	3	3	5	5
2	3	3	4	5	3	5	4	4	4
3	3	3	3	5	5	5	5	5	3
1	3	3	2	5	5	5	5	5	4
5	4	4	5	4	3	3	3	2	4

4	4	5	5	6	4	3	4	5	4
5	3	2	5	5	6	5	5	6	5
5	4	4	4	5	5	5	5	5	4
4	4	4	4	5	5	5	5	4	4
3	3	3	3	6	6	6	6	6	5
1	2	3	3	6	5	4	5	5	5
4	3	4	3	4	4	4	4	4	5
4	4	4	4	6	6	6	6	6	4
5	2	2	3	6	4	1	3	6	3
5	5	5	5	6	6	6	6	6	4
3	3	3	3	1	5	3	5	4	3
4	4	5	3	4	5	4	4	4	5
5	5	5	5	6	6	6	5	6	5
4	4	4	4	3	4	4	3	3	4
4	4	4	4	6	6	5	5	6	4
1	1	2	1	3	1	3	3	4	1
2	3	4	5	6	3	2	4	5	3
4	5	4	4	6	2	5	5	5	5
4	4	4	4	5	4	5	5	5	3
4	5	5	5	6	6	6	6	6	4
1	3	3	2	5	5	5	5	5	3
4	5	5	5	5	5	5	5	5	5
4	4	4	4	6	5	4	4	5	4
4	5	4	4	6	5	3	5	6	5
3	3	3	3	3	4	4	4	3	2
5	5	5	3	5	4	2	3	5	3
5	5	5	5	3	5	6	6	6	5
5	5	5	5	6	6	6	6	6	5
3	3	3	5	4	3	2	2	5	5
3	4	4	4	5	4	2	4	5	5
2	4	4	4	2	5	5	5	3	1
4	4	4	4	6	3	3	3	6	4
2	4	4	5	4	4	4	3	4	5
3	3	3	3	5	5	4	5	6	5
4	3	4	4	5	4	4	4	4	5
3	3	4	4	5	3	3	4	5	3
4	5	5	5	6	6	6	6	6	5
4	3	3	3	5	4	4	4	4	3
4	4	4	5	5	5	5	5	5	4
5	5	5	5	6	3	6	6	6	5
3	4	4	3	6	6	6	6	6	4
3	5	3	5	6	5	4	4	4	5
4	4	4	4	6	6	5	4	5	4
3	3	3	4	1	2	3	3	2	5
5	5	5	5	6	6	6	6	6	5
1	1	1	1	6	4	4	4	3	3
3	3	3	3	5	4	4	4	4	4
4	4	4	2	5	4	4	5	5	4
4	4	4	4	6	6	6	4	6	5
3	3	3	3	5	5	4	3	3	4

4	4	4	3	5	4	3	5	4	4
2	2	3	4	5	4	4	4	5	5
4	4	4	4	4	4	4	4	4	3
4	4	2	4	2	5	3	5	4	2
4	3	4	4	5	4	4	4	4	3
3	3	3	4	5	5	5	5	5	3
1	2	3	3	5	3	5	5	5	5
4	4	4	4	6	6	6	6	6	4
5	5	5	5	6	6	6	6	6	5
3	4	4	3	5	5	5	5	5	4
5	5	5	5	6	6	5	5	5	5
2	3	2	2	4	2	2	1	1	4
4	3	4	4	5	5	4	4	6	5
4	4	4	4	6	6	6	5	5	4
5	5	5	5	6	6	6	6	6	5
5	4	4	4	5	1	1	1	2	4
4	4	3	4	2	3	5	4	2	3
5	5	5	5	6	1	6	5	6	5
3	4	4	4	6	6	6	5	6	5
3	3	4	4	5	4	4	5	5	2
1	4	4	4	5	5	5	5	5	3
3	3	3	5	4	4	2	3	4	4
1	2	2	3	1	4	5	5	5	1
4	4	4	3	5	5	5	5	5	5
2	3	3	2	5	3	4	4	5	4
3	2	3	3	5	5	4	4	5	4
2	3	3	3	4	4	4	4	4	3
3	3	3	3	6	1	6	5	4	3
3	3	4	4	5	6	4	3	6	3
3	3	3	3	5	5	5	5	5	4
3	3	3	4	6	6	6	6	6	5
3	3	3	4	5	5	5	5	5	4
3	3	3	2	5	5	5	5	5	4
5	4	4	5	4	5	5	5	4	5
4	4	4	5	6	6	6	6	6	5
4	5	4	5	4	4	3	4	4	3
4	5	5	5	6	6	6	6	6	4
5	5	5	5	6	6	6	6	6	5
4	4	4	4	6	4	4	3	5	1
1	1	1	1	6	6	6	6	6	3
4	4	4	3	6	4	4	5	6	4
2	4	4	5	6	6	5	5	5	3
3	3	3	4	5	5	5	5	5	4
4	4	4	5	6	5	5	5	5	5
3	3	3	3	4	5	4	4	3	3
4	4	4	4	6	4	4	4	5	5
3	3	3	3	5	5	5	5	5	3
3	4	4	3	6	4	4	4	4	4
4	4	4	4	5	5	5	6	6	4
2	4	3	3	5	4	4	4	4	3

3	3	3	3	3	3	3	4	3	4
2	3	3	4	5	5	5	5	5	5
4	4	4	1	6	6	6	5	5	4
2	4	3	4	5	5	4	4	5	3
5	4	4	4	6	6	5	5	5	4
3	2	3	2	1	4	4	4	4	5
1	4	4	3	5	4	3	3	5	5
4	4	4	3	4	4	4	4	4	4
5	4	4	5	5	4	5	6	6	3
4	4	3	4	5	5	5	5	5	4
4	4	4	4	4	4	4	3	3	4
2	2	2	2	4	3	4	4	4	3
2	2	2	2	4	3	3	4	4	3
2	3	3	3	5	4	4	4	4	3
2	2	2	3	6	4	4	4	4	1
3	2	2	2	4	3	3	4	4	3
3	2	2	2	5	5	4	4	4	3
2	2	2	2	6	5	5	5	3	4
3	3	2	3	3	3	3	2	3	3
2	2	3	2	4	3	3	2	3	4
3	3	3	1	5	1	6	6	6	3
4	4	4	4	5	5	5	5	5	4
3	3	3	3	6	6	6	6	6	3
3	3	3	3	6	1	6	6	6	4
3	3	3	3	6	6	6	6	6	3
4	3	3	4	5	2	5	4	4	5
4	5	5	5	6	6	6	6	6	5
4	4	4	4	6	2	2	5	4	3
4	4	4	4	4	4	5	4	4	3
3	2	2	5	5	2	3	2	2	5
2	4	2	5	6	6	6	5	6	2
5	4	4	5	6	5	5	4	5	3
3	3	3	4	4	4	4	4	3	4
2	4	4	5	5	1	5	5	5	5
4	4	4	4	5	5	5	5	5	5
3	3	1	1	6	5	5	4	5	3
2	3	2	3	5	5	5	5	4	3
2	3	3	4	5	4	3	3	5	3
4	4	4	4	6	4	4	4	5	1
2	2	2	2	3	3	4	3	4	4
5	4	3	4	5	5	5	5	5	4
4	4	4	4	5	4	4	4	4	4
5	4	3	5	1	4	5	4	6	2
3	3	3	3	4	4	3	4	5	5
3	3	3	3	6	6	6	6	6	3
2	3	3	3	2	2	3	3	2	4
3	3	3	4	6	5	5	6	6	3
4	3	3	4	2	3	3	6	4	3
4	4	4	4	5	3	4	4	4	4
2	2	3	1	3	6	4	4	3	3

4	4	4	4	5	5	4	4	4	4
3	2	2	5	5	4	4	4	3	3
5	5	5	5	5	5	4	4	5	4
4	4	4	4	5	3	3	3	3	3
1	2	2	1	6	4	5	5	5	3
3	3	3	3	6	5	5	5	5	3
5	5	5	4	5	5	5	5	4	4
5	5	5	5	5	5	5	5	5	5
3	2	2	2	4	3	4	4	5	4
2	2	2	3	3	4	4	3	4	4
4	3	3	4	4	3	4	4	4	3
2	3	3	3	4	5	5	5	5	4
2	2	3	3	5	4	4	5	5	4
4	3	5	4	4	4	5	4	4	4
3	3	3	2	4	4	5	5	4	4
3	2	3	3	5	4	4	4	5	4
3	4	4	4	6	5	5	6	5	4
3	3	3	3	5	5	5	5	5	3
3	3	3	3	4	4	4	4	4	4
4	3	3	4	6	6	6	6	6	4
3	3	3	3	4	4	2	2	4	3
3	3	3	2	5	5	5	5	5	4
1	1	3	3	6	6	6	6	6	4
5	5	5	5	5	4	5	5	5	4
5	5	5	5	3	4	3	4	4	5
3	2	3	3	4	4	3	4	4	2
4	3	4	4	5	4	5	5	5	4
3	4	4	4	5	5	5	4	4	5
3	4	4	4	5	5	5	5	5	3
3	4	3	5	6	5	5	5	6	4
4	4	4	4	5	5	5	5	5	5
4	3	3	4	6	6	5	5	5	4
5	5	5	5	6	6	5	5	6	4
4	4	5	5	6	6	6	6	6	5
3	3	3	2	5	4	4	3	5	4
4	4	4	4	6	5	5	5	6	5
5	4	4	4	6	6	6	6	6	5
3	3	3	3	2	5	6	3	2	1
3	3	3	3	2	2	2	2	2	3
4	4	4	4	4	4	4	4	4	3
4	4	4	4	5	5	5	5	5	4
3	4	4	4	6	5	4	4	5	4
4	5	5	5	6	6	6	6	6	5
4	4	4	4	6	6	6	6	6	4
3	3	3	3	4	4	5	4	3	3
1	1	2	2	6	3	5	6	6	1
4	4	4	4	2	5	4	5	4	4
3	3	3	3	4	5	5	5	5	5
3	4	4	5	2	4	3	3	3	3
2	3	3	4	5	1	2	4	5	2

4	4	5	5	5	5	5	5	5	5
4	4	4	4	5	5	5	4	5	5
5	5	5	5	6	1	5	5	6	5
4	4	4	5	5	4	4	4	4	4
5	5	3	5	6	6	5	6	6	5
4	5	5	5	5	6	6	6	6	5
1	1	1	1	5	5	5	5	5	4
5	5	5	5	6	4	4	4	4	5
4	4	4	4	6	5	4	4	5	4
3	3	3	3	5	3	3	3	6	3
4	3	3	3	6	5	4	4	6	4
5	5	5	5	6	6	5	5	6	4
4	5	4	4	6	4	4	3	5	4
4	4	4	5	5	5	4	4	5	4
5	5	5	5	5	5	5	5	5	4
4	4	4	4	5	3	5	5	5	4
3	4	4	5	6	2	1	1	6	5
5	5	5	5	6	6	6	6	6	5
5	5	5	5	5	5	5	5	5	4
3	4	3	3	2	3	3	3	2	3
5	5	5	5	5	5	5	5	5	5
4	4	4	5	6	5	5	4	6	4
1	1	1	1	5	5	5	5	5	1
5	5	5	5	6	6	6	5	6	5
3	4	4	4	6	5	4	5	6	5
2	2	3	3	5	5	5	5	5	3
3	5	5	5	6	6	6	5	5	5
5	5	5	5	6	5	5	5	6	5
3	3	4	4	5	5	5	5	5	3
4	4	4	4	5	5	5	5	5	5
1	1	1	3	6	6	2	1	4	1
3	4	4	4	6	5	3	3	4	3
4	3	4	4	6	5	5	5	5	5
2	2	4	4	5	4	4	4	5	4
3	4	4	4	6	6	5	4	5	5
3	3	3	3	6	6	5	5	5	3
4	4	4	4	5	5	5	5	5	4
5	5	5	4	6	6	6	4	6	5
4	4	4	4	5	5	5	3	4	4
3	3	4	4	5	6	5	4	5	3
3	3	3	3	5	4	4	4	4	2
3	3	3	3	6	4	3	2	4	4
3	5	4	4	6	2	2	2	3	5
2	3	3	4	6	6	4	4	4	3
4	4	4	4	5	5	5	5	5	4
3	3	3	4	6	5	4	3	2	4
3	4	3	4	5	3	4	4	5	3
4	3	4	3	4	2	3	3	4	4
3	4	4	4	6	4	5	5	6	3
4	4	3	3	4	5	4	5	4	3

3	2	3	3	6	6	6	6	6	2
5	5	5	5	6	6	6	6	6	4
3	3	3	3	6	4	3	1	3	4
3	3	3	4	5	4	4	5	5	4
1	3	3	3	4	5	3	4	4	1
4	3	3	4	5	5	5	5	5	4
4	4	3	3	6	6	5	6	6	5
2	3	3	4	5	5	5	4	4	3
2	4	4	5	6	6	6	6	6	3
2	3	3	3	5	4	5	3	5	3
3	3	3	3	4	1	3	5	5	4
3	4	4	3	5	4	4	4	5	5
4	4	5	5	4	1	5	5	5	4
4	4	4	4	5	4	4	4	5	4
3	4	4	5	4	1	5	5	6	4
3	3	3	4	4	4	4	5	6	5
4	4	4	4	6	6	6	6	6	3
2	2	1	1	6	3	4	3	5	5
5	5	5	5	5	5	5	5	5	4
5	5	5	5	6	6	6	6	6	5
1	1	2	4	5	4	4	3	4	4
5	3	5	5	6	6	2	1	4	5
5	3	3	4	5	2	4	3	6	2
5	5	5	5	6	3	3	3	5	5
3	3	3	3	4	5	4	4	4	3
5	5	5	5	5	5	5	5	5	5
2	4	5	5	6	6	5	5	6	5
4	3	2	3	2	2	2	2	2	5
3	1	4	3	5	5	5	5	4	5
2	5	5	5	4	5	4	5	5	4
3	3	4	4	6	5	5	5	5	4
3	3	4	3	5	2	4	2	5	3
5	4	5	5	6	6	6	6	6	5
3	3	3	4	6	5	5	4	6	5
4	5	4	3	1	2	4	5	4	1
3	3	3	3	3	4	3	4	3	3
2	4	4	4	6	4	4	5	5	4
5	5	3	3	6	5	4	4	4	3
3	3	3	5	4	4	4	5	3	4
2	5	5	3	6	6	6	5	6	2
5	5	5	5	5	4	4	3	5	3
5	5	5	5	5	5	3	2	5	5
3	2	4	2	4	4	3	3	3	4
4	4	4	4	5	4	4	4	4	4
4	5	5	5	6	6	6	6	6	5
4	5	5	5	6	4	4	6	4	5
4	4	4	4	4	4	3	4	4	5
5	4	5	3	6	6	5	5	5	5
2	3	4	5	4	5	5	4	4	4
5	3	3	3	5	5	4	5	5	4

4	4	4	4	6	3	6	5	5	4
4	4	4	4	5	5	3	3	5	4
5	5	5	5	3	3	4	4	5	3
4	5	5	4	6	6	6	6	6	4
4	4	4	5	5	4	3	3	5	3
5	4	4	4	6	5	5	4	5	5
3	3	4	5	5	4	4	3	5	4
2	4	4	4	6	3	3	4	4	3
4	5	4	5	6	6	6	5	6	5
2	3	3	3	4	4	4	5	4	5
5	5	5	5	5	5	5	5	5	4
5	5	5	5	6	5	3	3	6	5
4	4	4	4	6	5	5	5	5	5
3	3	3	4	5	2	5	5	5	5
3	3	2	3	4	4	4	4	4	4
2	3	4	4	5	4	4	4	4	3
2	4	5	5	6	6	6	6	6	5
2	3	3	3	6	5	5	4	6	2
3	5	5	5	5	5	3	3	3	5
3	3	4	4	6	5	3	4	4	3
5	4	4	4	6	6	6	6	6	5
2	4	4	4	5	5	4	4	5	4
3	3	3	3	6	6	6	6	6	5
4	3	3	2	5	2	2	2	5	1
4	4	4	4	5	2	2	5	5	4
3	3	4	4	2	2	2	5	5	4
3	3	3	3	5	4	4	4	4	4
5	4	4	5	6	6	6	6	6	5
5	5	5	5	6	6	5	6	6	5
4	4	4	4	5	2	6	2	5	2
4	5	5	5	6	4	4	4	5	4
2	4	4	4	5	5	5	6	6	3
3	3	4	2	6	1	4	6	5	3
2	3	3	3	6	6	4	4	4	5
3	4	4	4	5	4	4	4	4	5
5	5	5	5	4	5	4	3	3	4
4	4	4	4	5	5	5	5	4	4
4	4	5	5	6	1	1	6	6	5
5	5	5	4	6	6	6	6	6	2
4	4	5	5	6	6	6	6	6	5
2	2	2	2	3	6	3	4	4	3
1	4	5	5	6	6	6	6	6	5
2	3	4	4	6	2	4	5	5	5
2	4	2	2	5	3	2	3	5	1
4	3	3	4	6	5	4	4	4	3
4	4	3	4	5	5	5	5	5	4
2	3	3	4	4	4	4	4	3	3
4	4	4	3	4	4	5	6	5	3
4	4	4	5	6	3	3	3	6	4
4	4	4	5	5	5	5	5	5	3

5	5	5	5	6	4	6	6	6	1
3	4	4	5	5	3	3	4	4	3
5	4	5	4	6	4	4	5	4	4
2	4	4	4	5	2	4	4	4	3
4	4	4	5	6	3	3	4	5	3
3	4	4	5	5	5	5	5	5	4
4	4	4	4	5	5	5	5	5	4
5	5	5	5	6	3	4	4	4	4
4	4	4	3	1	2	2	4	3	5
5	5	5	5	3	3	3	4	4	4
4	5	4	5	5	5	4	4	5	4
3	4	3	5	6	6	2	3	6	3
3	3	3	3	6	6	6	4	5	5
4	4	4	4	6	5	5	4	5	4
2	1	1	3	6	3	3	3	3	3
4	1	3	5	6	4	4	4	6	4
3	4	4	3	5	5	4	4	5	1
3	4	4	2	5	5	5	5	5	3
4	3	2	1	6	6	5	5	5	4
5	4	4	5	6	5	5	5	4	5
4	4	4	5	5	2	3	2	5	4
2	2	2	2	4	5	5	5	4	3
5	5	5	5	6	6	6	6	6	5
5	5	5	5	5	5	5	6	6	4
4	4	3	5	3	4	4	3	4	2
4	4	4	3	5	5	5	5	5	4
1	2	4	1	6	5	4	5	4	2
2	2	2	2	2	4	4	4	3	3
2	3	3	4	5	4	5	4	4	4
3	3	3	4	5	5	5	5	5	3
3	4	4	4	6	5	2	5	6	3
3	3	3	4	5	5	5	5	5	5
4	4	4	4	6	6	6	6	6	5
4	4	4	4	5	4	3	4	4	3
3	3	3	3	3	3	3	3	3	3
3	3	3	3	6	6	6	5	6	3
4	5	5	5	6	6	6	6	6	5
4	4	4	2	5	5	5	5	5	4
2	2	2	4	6	5	4	5	5	4
3	3	3	4	5	4	4	4	5	3
2	3	4	4	6	5	4	4	5	3
4	3	4	4	5	3	5	4	5	3
4	5	4	4	6	2	5	5	4	2
3	3	3	2	5	4	4	4	4	4
4	4	4	4	5	5	5	5	5	4
2	5	5	5	5	5	5	5	5	5
4	4	4	5	6	6	6	6	6	3
2	4	4	5	5	4	4	4	5	4
3	3	3	3	4	4	5	4	4	4
4	4	4	4	6	5	5	3	4	4

2	4	4	5	5	5	5	5	5	4
4	5	5	5	6	1	5	5	5	5
5	5	5	5	6	5	5	4	6	5
2	3	3	2	5	2	2	2	4	5
5	5	5	5	2	3	2	5	5	4
1	3	3	5	6	3	4	5	5	3
4	4	4	4	6	6	5	6	6	4
5	4	4	5	4	4	4	4	4	4
1	2	4	4	6	6	5	5	4	3
4	3	3	2	5	5	5	5	5	5
4	3	3	4	5	5	1	3	5	3
4	4	4	4	5	5	5	5	5	4
4	2	2	3	2	4	3	4	4	3
2	1	2	1	4	5	4	4	4	4
3	4	4	4	6	4	5	4	5	5
3	3	4	4	5	5	4	3	4	4
2	2	4	4	5	5	5	5	5	4
2	3	3	3	5	5	5	5	5	3
2	5	4	4	6	5	5	5	5	5
4	4	5	5	6	6	6	6	6	5
4	4	4	4	5	5	4	5	5	4
3	4	5	4	6	5	5	5	5	4
5	4	5	4	6	5	4	4	5	4
4	4	4	4	5	5	4	4	3	3
3	3	3	3	6	6	6	6	6	2
4	4	4	4	5	5	5	4	4	5
2	4	5	5	5	3	3	3	5	5
2	1	4	3	3	6	4	4	6	3
3	4	4	4	5	3	4	3	3	3
4	3	3	4	5	5	5	5	5	4
2	2	3	3	6	6	5	4	4	4
3	3	3	4	1	2	2	2	2	4
3	3	3	2	5	5	4	4	5	3
1	1	1	1	4	4	4	4	4	1
3	4	3	3	6	5	4	3	4	4
3	1	3	3	4	3	3	2	3	4
5	5	5	5	5	6	5	6	6	4
5	5	4	5	6	5	5	5	5	5
4	4	3	4	1	6	6	6	6	5
3	3	3	3	6	4	4	4	3	3
4	3	3	4	5	4	5	5	5	4
4	4	4	3	3	2	3	4	3	3
3	3	3	4	4	4	2	2	3	4
4	4	4	4	4	3	3	5	5	4
4	4	4	5	5	4	4	3	5	3
2	3	5	4	5	4	3	2	5	5
4	4	4	5	5	5	5	5	5	5
5	5	5	5	6	4	4	5	6	5
3	4	4	4	4	4	4	3	4	3
4	4	4	4	5	5	5	5	5	3

3	4	4	4	5	3	3	3	5	5
4	4	4	4	5	5	5	5	5	5
4	2	3	3	5	2	3	4	5	3
3	5	5	5	5	3	5	6	6	3
4	3	4	4	1	3	2	2	2	4
4	4	4	3	5	5	5	5	5	4
2	3	3	4	5	5	2	2	4	5
3	2	3	4	5	5	5	5	5	5
4	5	5	5	5	5	4	4	5	4
4	4	4	5	5	2	5	5	5	5
4	3	4	1	6	5	5	5	5	5
4	3	3	4	5	5	4	4	4	4
4	3	4	4	4	5	4	4	5	3
4	4	4	4	2	3	2	2	3	3
1	3	3	5	6	5	5	4	5	5
4	3	4	4	5	4	4	4	5	5
2	4	4	2	1	1	3	3	2	3
3	3	3	3	4	3	3	3	4	3
4	5	5	5	5	4	4	4	4	3
4	4	4	4	6	5	4	4	6	5
3	4	4	4	6	5	5	5	6	5
2	2	2	1	6	3	3	2	4	2
3	4	4	4	4	4	4	4	4	4
4	4	3	3	4	4	4	4	4	3
3	4	4	3	5	4	4	4	4	4
1	1	1	3	3	2	2	1	1	5
2	3	3	4	5	5	5	5	5	5
5	4	4	4	5	4	2	2	5	4
2	2	2	3	5	6	5	5	5	1
2	3	3	3	5	5	4	4	4	2
4	5	5	5	5	5	5	5	5	5
3	3	4	4	5	4	5	5	5	3
5	5	3	5	5	5	5	5	5	5
4	4	4	4	5	4	3	4	5	4
4	2	2	2	6	6	4	4	5	3
2	3	4	4	5	5	5	5	4	3
1	1	2	2	6	6	6	6	5	5
4	3	5	4	6	6	5	5	5	3
3	4	4	4	5	5	4	3	5	4
3	4	4	4	5	4	4	4	4	3
4	3	4	5	6	5	5	5	5	3
3	4	3	4	6	6	6	6	6	3
4	5	3	5	6	5	5	5	5	2
4	4	4	3	4	5	5	5	3	3
1	5	5	1	6	2	1	1	1	5
4	5	3	3	5	4	4	5	5	4
5	4	5	4	6	5	5	5	5	4
1	1	1	1	5	4	3	4	4	4
5	4	4	4	5	4	4	4	4	4
1	3	3	3	6	6	5	6	6	3

4	5	5	5	6	6	4	4	4	4
3	3	4	4	1	6	5	4	4	4
4	4	3	4	6	6	6	6	6	5
5	5	5	5	5	5	4	5	6	5
5	4	4	4	5	5	5	5	5	4
3	4	2	4	5	3	5	4	5	3
4	3	4	4	6	1	6	6	6	4
3	3	4	4	3	4	4	4	4	4
4	4	4	4	5	3	4	5	5	4
3	4	3	4	2	3	2	4	4	3
5	5	5	5	6	4	3	4	3	3
5	5	5	5	6	5	3	4	4	4
3	5	5	5	5	6	5	6	6	3
3	4	4	4	5	5	4	5	4	4
2	5	5	5	6	5	5	5	6	5
5	4	4	4	5	5	4	5	5	4
5	5	5	4	6	3	3	3	4	4
2	4	4	4	5	5	1	3	6	5
5	3	2	4	3	2	2	4	4	4
3	4	4	4	5	5	4	4	3	5
4	3	4	4	6	5	5	6	6	5
4	5	5	5	5	5	5	5	5	3
3	3	2	4	5	5	5	6	5	3
4	3	5	5	6	6	6	6	6	4
2	2	2	5	4	4	4	5	6	4
4	4	4	4	6	6	5	5	5	5
5	5	5	5	6	6	6	6	6	5
5	5	5	5	6	1	1	1	6	3
4	5	5	4	5	3	3	3	4	4
2	1	5	3	6	1	5	5	5	5
4	4	4	5	6	5	5	5	5	5
3	4	4	4	4	3	1	3	2	4
4	3	4	4	4	5	4	4	4	4
4	3	3	2	6	5	3	3	6	5
4	4	3	4	6	6	6	6	6	4
4	3	3	4	5	6	5	4	5	3
2	4	4	4	6	4	4	4	5	3
4	4	4	4	6	2	2	3	6	5
5	5	5	5	6	1	6	6	6	5
4	4	5	4	5	5	5	5	4	4
3	4	4	4	5	5	5	5	2	1
5	4	4	4	6	6	6	6	6	3
2	4	3	4	5	6	4	5	4	3
2	4	4	4	6	4	4	5	4	4
5	4	5	5	6	6	6	6	6	5
3	3	4	5	6	3	4	4	4	5
3	3	4	4	3	4	2	4	4	3
3	3	3	3	6	5	5	5	6	5
2	4	4	4	6	4	4	5	5	2
2	1	1	4	5	4	4	4	5	3

3	3	3	4	6	5	5	4	4	3
5	5	5	5	5	5	5	5	5	5
5	5	5	5	6	6	6	6	6	5
3	3	3	3	5	5	5	5	5	4
4	5	5	5	4	2	2	2	5	5
3	5	5	4	5	5	5	5	6	3
3	4	4	4	6	6	6	6	6	5
2	3	3	3	6	4	2	2	6	5
3	5	5	5	6	6	6	6	6	5
4	3	3	3	6	3	5	5	4	5
5	5	5	5	6	5	4	5	5	4
5	5	5	4	6	1	6	6	6	5
3	4	4	4	5	5	4	4	4	4
2	4	4	5	1	1	1	1	1	5
4	5	5	5	4	4	4	4	4	5
2	2	2	2	4	4	3	4	4	5
1	4	4	5	5	6	1	1	6	3
3	4	4	4	4	6	5	4	4	5
2	3	3	4	5	4	3	4	4	3
1	4	3	5	6	6	5	4	6	3
3	4	4	4	5	5	5	5	5	4
3	3	3	4	1	1	1	1	1	3
4	4	4	5	6	6	6	6	6	3
5	5	5	2	6	5	5	5	5	2
5	5	5	5	6	6	5	5	6	5
5	5	5	3	3	4	2	4	4	5
4	4	4	4	3	2	2	5	3	4
3	1	4	4	6	5	5	4	6	1
3	4	3	3	5	5	4	5	5	4
3	3	3	4	5	6	5	5	5	1
5	5	5	4	5	5	3	4	4	4
4	4	4	4	6	6	6	6	6	5
3	3	4	4	6	5	4	5	5	3
3	4	4	4	6	6	6	6	6	5
4	3	3	3	5	5	5	5	5	3
3	3	3	4	6	4	4	4	5	4
3	3	3	4	3	4	4	4	6	3
5	5	5	5	6	5	5	2	6	4
3	4	4	4	6	3	3	4	5	4
3	3	4	4	4	3	4	2	5	3
5	4	5	4	6	5	5	4	4	4
1	1	1	1	6	5	5	5	5	5
3	3	3	3	3	3	3	3	3	5
4	3	3	1	6	6	5	5	5	4
4	5	5	4	6	6	4	5	5	4
5	4	5	5	6	5	6	4	5	4
2	3	3	3	5	4	4	4	5	5
2	4	4	4	6	5	5	5	6	4
2	4	4	4	4	5	4	4	4	3
4	3	3	5	5	5	5	4	4	4

4	4	5	5	6	6	6	5	5	1
3	4	5	5	5	1	5	4	5	4
3	4	4	4	5	3	3	3	4	3
1	1	4	3	2	6	3	1	4	3
4	4	5	5	5	4	4	5	4	5
3	3	2	2	5	3	3	3	5	3
4	4	4	4	6	1	6	5	5	4
3	2	2	4	6	4	4	4	5	4
4	4	4	5	6	6	3	5	5	5
2	3	3	3	5	5	5	5	5	4
4	4	4	3	6	5	5	4	5	4
2	4	4	4	6	6	5	5	5	4
3	3	3	4	6	3	3	4	5	3
4	4	4	4	5	6	6	6	6	4
3	3	4	4	6	6	6	6	6	5
4	4	4	4	3	1	5	4	4	4
4	4	5	4	5	5	5	5	5	5
3	4	4	5	6	3	3	3	4	3
3	3	4	4	6	5	5	5	5	4
4	4	3	4	5	2	4	4	5	3
3	3	3	1	3	6	5	6	6	5
4	4	4	5	6	6	6	5	6	4
4	4	4	4	6	4	3	3	6	3
2	3	4	4	5	5	5	6	6	4
3	4	4	3	5	5	5	5	5	4
5	5	5	5	1	1	6	4	6	5
4	4	4	3	6	1	6	6	5	3
4	4	4	4	4	4	5	3	4	3
4	5	5	5	5	5	4	4	5	3
5	5	4	5	6	5	4	5	5	4
4	5	5	5	5	5	6	6	5	5
5	5	5	5	5	4	4	4	5	5
5	5	5	5	3	3	5	1	6	3
4	4	5	5	5	2	6	6	5	5
4	5	5	5	6	4	4	4	5	5
4	4	4	4	6	4	3	3	3	5
4	5	5	5	6	6	5	5	6	4
4	3	4	5	5	3	4	4	4	3
2	3	3	3	2	3	2	5	3	2
5	3	3	1	6	5	5	6	5	5
4	4	5	5	5	5	5	5	5	4
1	3	4	4	6	5	5	5	6	5
4	4	4	3	5	2	3	3	3	4
3	3	2	4	5	4	3	3	3	3
2	2	4	5	6	5	4	4	4	1
4	4	4	4	6	5	3	3	4	3
3	3	3	3	5	5	5	5	5	3
3	5	5	5	6	3	3	3	5	4

P_InvApFa	P_InvApFa	P_InvApFa	P_InvApFa	P_InvApFa	P_InvApFa	P_InvApFa	Madre_HE	Padre_HEI	Hijo_HEI
3	3	3	3	3	3	3	74.5	37.5	81.5
5	4	4	4	4	4	4	82.0	82.0	85.5
5	5	5	5	5	5	5	66.5	69.0	73.5
5	4	4	5	5	4	5	68.5	68.0	83.0
5	4	4	3	4	4	5	85.5	84.0	50.5
5	5	5	5	5	4	5	72.0	63.0	70.5
5	5	5	3	5	5	5	59.0	49.5	47.0
5	4	5	4	4	4	5	62.0	56.0	73.0
5	4	5	4	4	4	5	48.5	53.5	64.5
5	2	3	2	3	4	3	77.5	55.5	54.5
4	4	5	5	5	5	5	47.5	63.5	59.5
3	5	5	3	3	4	4	77.5	61.0	72.0
4	5	4	2	4	4	4	69.0	57.0	57.0
3	4	4	4	4	4	4	85.0	85.0	85.0
3	5	4	4	4	4	5	56.0	53.5	51.0
2	4	4	4	4	4	4	73.5	53.5	53.5
4	4	4	4	4	4	4	67.0	48.0	75.5
4	3	4	5	4	3	2	30.0	43.5	73.0
5	5	5	5	5	5	5	65.5	63.0	79.0
4	4	4	5	5	4	4	58.5	57.0	55.0
3	3	3	3	3	3	3	52.5	58.5	59.5
5	4	4	4	4	4	4	50.0	62.0	45.0
3	4	4	4	4	4	4	57.0	62.0	46.0
3	3	3	3	4	3	3	74.5	69.5	62.0
4	4	4	3	4	4	4	66.0	77.0	66.0
4	4	4	5	5	5	5	63.5	56.0	56.0
5	5	5	5	5	5	5	53.5	51.0	73.0
4	4	3	3	3	4	4	67.5	51.0	65.0
4	3	3	4	4	4	4	60.0	74.5	69.5
5	5	5	5	5	5	5	79.5	82.0	72.5
4	4	5	4	4	4	4	79.0	87.5	82.5
3	3	3	3	3	3	3	68.5	74.0	65.5
5	3	5	5	5	4	4	64.5	80.5	75.0
4	4	5	4	5	4	4	70.0	68.0	57.5
5	5	5	5	4	4	5	78.5	76.5	57.0
4	5	4	4	4	4	4	42.5	43.5	48.5
5	5	5	5	5	5	5	48.5	38.5	65.0
3	3	4	4	3	4	4	57.5	60.5	55.5
5	5	5	5	4	4	5	74.5	76.5	82.0
2	3	3	4	3	3	2	52.0	56.0	47.5
5	4	4	4	4	4	4	53.5	40.0	70.0
3	4	4	4	4	4	4	54.5	37.0	66.5
5	5	5	5	5	5	5	59.5	62.0	65.0
5	5	4	5	5	5	5	69.0	59.0	69.5
3	5	4	4	5	5	4	48.0	69.0	43.5
3	3	3	5	4	3	4	60.0	43.5	77.5
5	5	5	5	5	5	5	79.0	74.5	76.5
5	5	5	5	5	5	5	54.0	52.0	51.5
3	4	3	3	2	3	3	47.5	39.5	67.0

5	5	5	5	5	5	5	72.5	54.5	67.5
3	4	3	3	4	4	4	65.0	55.0	55.5
4	4	4	4	4	4	3	65.5	59.5	76.0
4	3	4	3	4	4	5	79.0	57.0	48.0
3	3	4	3	4	4	3	94.0	72.5	100.0
5	5	5	5	5	5	5	63.5	58.5	59.0
3	5	3	3	5	4	4	52.5	45.0	67.0
3	4	4	4	5	4	4	69.5	50.0	72.0
5	5	5	5	5	5	5	63.5	73.0	90.5
4	1	2	3	4	3	2	61.0	63.5	70.0
5	4	4	4	4	4	4	76.5	57.0	67.0
3	4	1	1	3	4	4	49.5	27.5	41.0
3	3	3	3	4	3	3	70.0	56.0	64.0
4	5	3	3	5	4	4	64.0	82.5	39.5
5	4	3	4	4	4	4	45.0	52.0	61.5
5	5	5	5	5	5	5	70.5	64.5	66.0
5	5	3	5	5	5	5	55.5	43.5	53.0
4	4	4	4	4	4	4	79.5	48.5	65.0
4	4	4	4	4	4	4	57.0	48.5	76.0
3	3	3	3	3	3	3	42.5	52.0	48.5
5	5	3	3	3	5	5	44.5	50.5	55.5
5	5	4	5	5	4	5	70.0	50.0	69.0
3	3	3	3	3	3	5	42.0	43.5	51.0
5	5	5	5	4	4	3	43.5	58.0	57.0
4	4	4	5	3	3	4	64.5	62.5	75.5
4	4	4	4	4	4	4	62.5	65.5	62.0
3	3	3	3	4	4	4	49.5	51.5	41.5
3	3	4	3	3	3	3	66.5	59.5	70.5
4	5	5	5	5	5	5	59.5	63.0	57.0
3	3	3	3	3	3	3	43.5	37.0	68.5
4	4	4	4	4	5	5	87.5	90.0	95.0
5	5	5	5	5	5	5	93.0	68.0	81.5
5	5	5	5	5	5	5	69.5	64.5	62.0
5	5	5	5	5	5	5	74.0	68.0	90.5
4	3	4	4	3	4	3	66.5	48.5	64.0
5	4	5	5	3	3	3	52.5	54.5	53.5
5	5	5	5	5	5	5	49.5	57.0	53.5
3	3	3	3	3	3	3	59.5	51.0	71.0
4	4	4	4	4	4	4	81.0	79.0	68.5
4	4	3	4	3	3	4	48.0	56.0	43.0
4	3	4	3	3	4	3	36.0	40.5	58.0
2	4	4	3	3	3	4	40.0	38.5	49.5
5	4	5	3	3	3	4	56.0	46.0	73.5
4	4	4	4	4	4	4	52.5	66.0	52.5
5	5	3	5	3	3	3	64.5	48.0	58.5
4	4	4	4	4	4	4	75.0	45.0	49.5
2	3	3	3	3	3	3	47.0	39.5	45.0
4	4	4	4	4	4	5	57.0	49.5	69.0
3	4	4	4	3	3	5	64.0	47.0	78.0
3	4	4	4	4	4	4	56.5	34.5	52.0

5	5	5	5	5	5	5	55.5	55.5	72.0
5	5	5	5	5	5	5	44.5	54.5	54.5
5	5	5	5	5	5	5	65.5	68.0	62.5
4	4	4	4	3	3	4	75.0	41.0	69.5
5	5	5	5	5	5	5	60.5	70.5	65.5
4	3	4	4	4	3	4	66.0	49.5	60.5
4	4	4	4	3	3	3	77.5	55.0	86.5
3	3	3	3	3	3	3	57.0	42.5	37.0
5	3	3	4	2	3	4	54.0	50.5	49.5
4	4	4	4	4	4	3	74.5	68.0	57.5
3	3	3	3	3	3	3	47.5	52.5	60.5
2	1	2	1	1	1	3	65.5	57.0	77.0
5	4	4	4	4	4	4	70.0	48.5	81.0
5	5	5	5	5	5	5	95.0	87.5	91.0
4	4	4	5	3	4	4	37.5	47.5	51.0
5	5	5	5	5	5	5	65.0	74.0	64.0
4	4	4	4	4	4	4	71.5	41.0	36.0
4	2	3	3	2	3	4	43.5	43.5	50.5
4	5	4	4	5	5	5	65.0	45.0	68.5
4	4	3	4	3	5	4	56.0	51.0	64.0
4	3	3	4	3	3	4	65.5	66.5	70.0
3	3	3	3	3	3	3	57.0	57.0	52.5
5	4	4	4	4	4	3	59.5	57.0	62.5
5	5	5	5	5	5	5	52.0	57.0	53.5
3	3	4	4	4	3	4	59.5	64.5	69.5
5	5	5	4	5	5	5	56.0	62.0	65.5
4	5	5	5	5	5	5	73.0	80.0	87.0
4	4	4	4	4	4	4	70.5	80.0	65.0
5	4	4	5	5	5	5	62.5	57.0	61.0
4	4	4	4	4	4	4	60.0	45.0	50.0
5	5	4	4	3	4	5	58.5	52.5	66.0
3	3	3	3	3	3	3	69.5	81.5	84.0
3	3	3	3	3	3	3	57.5	52.5	73.5
4	4	4	5	5	4	4	57.0	58.0	71.0
5	5	5	5	5	5	5	83.5	84.0	79.5
5	4	5	5	5	5	5	42.0	44.5	54.0
5	5	5	5	5	5	5	68.0	64.5	58.5
5	5	4	5	3	3	5	68.5	58.5	75.5
4	4	4	3	2	4	4	51.0	53.5	56.0
5	5	3	5	5	5	5	83.5	57.0	87.0
5	5	5	5	5	4	4	63.5	74.5	55.5
5	5	5	5	5	5	5	76.5	43.5	76.5
4	4	4	5	4	3	4	57.0	42.0	61.5
5	5	5	4	5	5	5	43.5	72.5	35.5
4	4	4	4	4	4	5	75.5	75.0	76.0
5	5	5	5	5	5	3	57.0	65.0	62.0
4	3	3	4	3	3	4	58.5	63.5	56.0
5	5	4	5	5	5	5	64.0	66.0	66.0
4	4	4	4	4	4	4	66.0	91.0	86.0
5	4	4	3	4	4	4	50.0	47.5	45.0

5	4	3	4	3	4	4	47.0	47.0	50.5
1	5	5	5	5	5	5	75.0	74.0	72.5
5	5	4	5	3	5	4	42.5	50.0	78.5
3	4	3	2	4	4	4	35.0	42.5	57.5
3	3	3	3	3	3	3	62.5	41.0	68.0
3	5	4	4	4	4	4	52.0	49.5	76.0
5	5	5	5	4	5	4	40.0	64.0	47.5
3	1	4	3	4	4	4	68.0	31.0	69.5
3	3	3	3	3	3	3	56.0	40.0	56.0
5	5	5	5	5	5	5	80.0	80.0	88.0
3	5	3	4	4	3	5	84.5	72.5	84.5
4	4	3	3	2	3	4	72.5	65.0	69.0
5	4	4	4	4	4	4	55.5	62.5	53.0
5	5	5	4	4	5	5	79.0	68.5	55.5
4	4	4	4	4	5	5	83.0	83.0	82.0
4	4	4	4	4	4	4	59.5	49.5	59.5
5	5	5	5	5	5	5	85.0	52.5	80.5
4	5	5	4	4	4	4	57.0	64.5	53.5
5	5	5	5	5	5	5	82.0	44.5	74.5
5	5	4	5	5	5	5	62.0	69.0	85.0
4	4	4	4	4	4	3	62.5	51.0	46.0
5	5	5	5	5	5	5	43.5	61.5	67.5
3	3	4	2	3	3	4	71.0	67.5	57.0
4	4	4	4	4	4	4	66.0	53.0	39.5
3	2	2	2	2	2	2	52.5	67.0	62.5
3	3	3	5	5	3	3	72.0	48.5	59.5
3	5	3	4	4	4	4	72.5	46.0	53.0
5	4	5	5	5	4	4	76.5	56.0	83.0
5	4	5	5	4	5	5	68.0	75.5	58.0
5	4	5	5	3	4	3	73.5	38.5	83.0
3	3	3	3	3	3	4	51.0	81.5	55.0
3	4	4	4	4	4	4	62.0	67.0	61.0
3	3	4	3	3	3	3	56.0	52.0	48.5
4	4	4	3	4	4	3	52.5	57.0	47.5
4	5	5	5	5	5	5	62.0	59.5	46.0
4	4	5	5	4	4	4	67.0	67.0	59.5
3	4	4	3	4	4	4	76.0	74.0	66.5
4	4	4	2	2	2	2	58.5	56.0	60.0
5	5	5	5	5	5	5	64.0	53.5	67.5
4	4	3	4	4	4	3	65.0	43.0	41.0
4	4	3	4	4	3	5	61.0	69.5	69.5
5	5	5	5	3	3	3	71.5	64.0	77.5
5	5	5	5	4	4	5	71.5	64.0	70.5
3	4	4	5	4	5	5	78.5	58.0	50.0
3	3	3	3	3	3	3	56.0	37.5	52.5
5	5	5	5	4	4	3	66.5	54.5	62.0
5	4	4	4	4	4	4	67.0	62.5	69.5
3	3	3	3	3	3	3	74.5	78.0	75.5
3	3	3	3	3	3	3	52.5	52.5	61.5
5	5	4	2	5	4	5	58.0	64.5	79.0

3	3	3	3	2	3	3	56.0	63.0	42.5
5	5	5	5	4	3	5	63.5	57.5	48.5
4	4	5	4	3	5	4	80.5	52.5	82.0
5	5	5	5	5	5	5	56.0	56.0	84.0
5	4	4	4	4	4	4	58.0	53.0	53.5
5	3	5	5	3	3	4	32.5	43.5	46.0
5	5	5	4	3	4	3	65.5	62.5	55.0
4	3	5	4	4	3	3	62.5	52.5	42.0
3	3	3	3	3	3	3	49.5	53.0	52.0
4	4	4	4	4	4	5	82.0	77.0	77.0
3	3	3	3	3	3	3	42.5	32.5	43.5
5	5	4	4	4	5	4	63.5	70.5	64.5
5	5	5	5	5	5	5	72.5	65.5	79.5
4	5	4	4	5	5	5	65.5	67.5	68.0
5	4	4	3	4	3	3	79.0	58.0	87.0
1	5	5	5	5	5	5	33.5	30.0	40.0
5	5	5	5	5	5	5	36.0	36.0	50.5
5	5	5	2	4	3	3	92.5	58.0	72.5
4	3	4	3	4	3	3	67.0	62.0	54.5
5	4	5	4	5	5	5	49.0	77.0	70.0
5	3	4	4	3	3	2	60.5	55.5	57.0
5	5	5	5	5	5	5	82.0	47.5	64.5
5	4	4	4	4	4	4	77.0	61.0	73.0
4	4	5	5	4	5	4	75.0	42.5	81.0
3	3	3	3	3	3	3	50.0	52.5	55.0
4	5	2	4	5	5	4	58.0	25.0	60.5
3	5	5	4	5	5	5	68.5	62.5	64.0
5	5	5	5	5	5	5	66.5	68.0	65.5
5	3	5	5	3	3	4	60.5	45.5	77.0
5	5	5	3	5	5	5	58.0	60.5	64.0
4	5	4	4	4	3	5	61.5	58.0	52.0
4	4	4	4	4	4	4	48.0	45.5	74.5
5	5	5	5	5	5	5	68.5	69.5	64.5
5	5	5	5	5	5	5	50.0	66.0	52.0
5	5	4	4	3	3	3	60.5	43.5	58.0
3	3	4	2	4	4	3	66.5	58.5	56.5
3	5	5	3	5	5	5	78.0	80.5	83.0
5	4	4	3	3	4	3	57.0	53.0	55.0
5	5	5	4	5	5	5	71.0	70.0	81.5
5	5	5	5	5	5	5	66.5	71.0	69.0
5	5	5	5	5	5	5	57.5	58.0	67.0
5	5	5	5	5	5	5	61.0	48.5	53.5
5	4	4	5	3	5	2	71.5	70.5	74.0
5	5	5	5	5	5	5	41.0	82.5	50.0
5	5	5	5	5	5	5	69.0	61.5	80.0
3	3	3	3	3	3	3	31.0	48.5	53.5
5	4	4	4	4	5	5	72.5	63.5	68.5
3	4	4	3	4	3	3	59.5	41.0	71.0
5	5	5	5	5	3	5	82.0	71.0	81.0
3	3	3	1	3	4	4	72.0	68.0	54.5

5	5	4	3	4	4	3	63.0	51.0	53.5
5	4	3	4	4	4	5	52.0	55.0	47.5
3	4	4	4	4	4	4	71.5	71.5	78.5
4	3	4	4	5	2	5	48.5	32.5	50.5
3	3	3	3	3	3	3	54.5	52.0	55.5
4	4	4	4	4	4	4	81.0	83.5	76.5
5	5	5	5	5	5	5	69.0	60.5	65.5
4	4	4	4	4	4	4	67.5	66.0	70.0
5	5	5	5	5	5	5	65.5	70.5	70.0
3	3	4	4	4	4	3	85.0	60.5	63.0
5	5	5	5	5	5	5	86.5	79.0	74.0
4	4	4	3	4	4	4	69.0	61.0	68.0
4	4	5	5	3	4	5	87.0	69.0	82.5
3	4	4	4	4	4	4	72.5	76.0	72.5
5	5	5	5	5	5	5	61.0	84.0	75.5
3	2	3	5	3	3	1	56.0	26.0	68.0
3	4	4	4	5	3	3	65.0	57.0	53.5
5	4	4	4	4	4	4	62.0	63.5	66.0
5	5	5	5	5	5	5	64.0	60.0	82.0
4	4	3	3	4	4	4	54.5	40.0	55.5
3	3	3	3	3	3	3	41.0	33.5	62.0
5	4	3	4	2	4	3	39.5	53.5	43.0
4	3	3	3	2	2	3	52.5	69.0	73.0
5	5	5	5	5	5	5	78.0	80.5	80.5
2	2	4	2	3	3	2	74.0	48.5	76.5
3	3	4	2	3	4	3	65.0	40.0	65.5
3	3	3	3	3	3	3	36.0	47.0	59.5
3	3	3	3	3	3	3	73.5	74.0	78.5
3	5	4	4	4	5	5	35.0	56.5	37.0
4	4	4	3	3	3	4	69.5	70.0	72.0
5	5	5	5	5	5	5	72.0	64.0	63.5
4	4	4	4	4	4	4	57.0	51.0	58.5
5	5	5	5	5	5	5	64.5	62.5	67.0
5	5	5	4	4	4	4	52.5	50.0	50.0
5	5	5	5	5	5	5	60.5	58.5	63.5
3	3	3	3	3	3	3	69.0	56.0	81.0
5	5	5	5	5	5	5	70.0	53.0	65.0
5	5	4	5	5	5	5	78.5	60.0	54.0
5	5	5	5	5	5	5	60.5	45.0	59.5
5	3	5	3	3	3	5	70.0	52.5	72.0
3	3	3	3	4	4	5	33.5	27.5	86.5
5	5	2	4	5	5	5	80.5	67.5	76.5
4	4	4	4	4	4	4	47.0	52.5	55.0
5	5	5	5	5	5	5	84.5	82.0	89.0
3	3	3	3	3	3	3	78.5	43.5	66.5
5	4	4	4	4	4	4	45.0	48.0	59.0
3	3	3	3	3	3	3	37.5	40.0	49.5
5	4	5	3	4	4	4	54.5	42.5	65.5
4	4	4	4	4	4	3	75.0	67.0	74.5
3	4	3	3	4	4	4	78.0	69.0	62.0

4	4	3	4	3	3	4	66.5	47.5	59.5
5	4	3	5	3	4	4	57.5	48.0	63.0
5	4	4	4	4	4	2	74.0	71.5	79.0
3	5	3	3	4	4	5	72.0	57.5	67.5
4	4	4	4	5	4	4	57.0	70.0	65.0
5	4	4	4	4	4	4	55.0	62.0	60.5
5	5	5	5	5	5	4	42.0	42.0	52.0
4	4	4	4	4	4	4	63.5	43.5	63.5
3	5	3	4	5	5	5	78.0	59.0	47.0
4	4	4	4	4	4	4	57.0	52.5	56.5
4	5	5	4	4	4	4	54.5	64.5	51.0
3	4	4	4	4	4	4	57.0	59.5	56.0
3	4	4	4	4	4	4	59.5	57.0	57.0
4	4	4	5	5	5	5	54.5	62.0	57.0
1	1	1	1	1	1	1	63.5	54.5	57.0
4	4	4	4	4	4	4	51.0	64.5	51.0
4	3	3	4	4	4	4	53.5	52.5	42.5
5	4	5	2	3	3	4	56.0	72.0	62.0
4	4	4	4	3	4	5	56.0	46.0	43.5
4	5	5	5	5	5	5	46.0	48.5	40.0
3	3	3	3	3	3	3	74.0	88.0	90.5
4	4	4	4	4	4	4	57.0	57.0	57.0
3	3	3	3	3	3	3	52.5	52.5	52.5
4	4	4	4	4	4	4	52.5	53.0	53.0
3	3	3	3	3	3	3	52.5	52.5	52.5
5	5	5	5	4	4	4	70.0	58.5	60.5
5	5	5	5	5	5	5	72.0	84.0	75.5
3	4	4	4	4	4	4	70.5	73.0	72.0
3	3	3	3	4	4	3	53.5	59.5	81.0
5	4	5	5	3	2	3	37.5	40.0	39.5
2	2	4	3	3	3	4	71.0	59.0	71.5
5	5	4	4	5	4	5	45.5	60.0	66.0
5	4	4	4	3	3	4	79.0	42.5	70.5
5	5	5	5	5	5	5	59.0	71.5	59.5
5	5	5	5	5	4	4	77.0	79.5	74.5
5	4	4	4	3	4	3	44.0	54.0	46.0
3	5	4	3	3	3	4	64.5	61.5	21.0
5	4	5	5	3	4	5	71.5	50.5	76.5
3	2	3	2	3	4	3	74.0	62.5	64.0
4	4	4	4	4	4	4	47.0	38.5	64.0
4	4	4	4	4	4	4	50.5	71.0	60.5
4	3	4	3	4	3	4	63.0	61.0	54.5
5	4	4	4	5	5	4	68.0	59.0	82.0
5	4	3	5	3	4	3	71.5	57.0	62.0
3	3	3	3	3	3	3	88.0	87.0	87.0
4	4	4	4	4	4	4	43.5	33.5	64.0
3	3	3	3	3	3	3	52.5	58.5	52.5
3	5	4	4	4	4	3	36.0	27.5	53.5
4	4	4	4	4	5	4	64.0	60.5	76.0
3	3	3	3	2	2	3	37.5	47.0	48.5

4	4	4	4	4	4	4	61.5	52.0	58.5
3	3	3	2	3	3	4	59.0	58.5	71.0
4	4	4	4	4	4	4	58.5	58.0	62.5
3	5	3	3	3	3	3	42.5	59.5	45.0
4	3	4	3	3	3	4	61.0	74.0	59.5
4	4	4	4	4	4	4	82.0	51.0	53.5
4	4	5	4	4	4	4	68.0	63.5	82.0
5	5	5	5	5	5	5	72.5	64.0	71.0
5	5	4	5	5	5	5	61.0	57.0	47.5
4	4	5	5	5	5	5	51.0	53.5	50.0
4	3	4	4	3	2	3	48.5	53.5	59.5
4	5	5	5	4	4	4	56.0	62.0	56.0
5	4	4	4	5	5	5	58.5	67.0	61.0
3	4	4	5	4	3	3	61.0	58.5	58.5
4	5	4	4	5	4	4	50.0	61.0	47.5
4	3	5	5	4	4	4	53.5	56.0	64.5
5	5	5	5	5	5	5	37.5	62.0	42.5
3	3	3	3	3	3	3	68.5	67.5	71.0
3	3	4	4	3	3	4	69.0	59.5	71.5
3	3	3	3	4	4	4	75.0	73.5	66.5
3	3	3	3	3	3	3	49.5	59.5	62.0
4	4	4	4	4	4	4	70.0	64.5	61.0
4	4	4	4	4	4	4	63.0	80.5	78.0
4	4	4	4	4	4	5	51.0	71.5	65.0
5	5	5	5	5	5	3	54.5	41.0	60.0
3	3	4	4	3	4	2	64.5	59.5	58.5
4	4	4	1	3	3	4	78.5	35.0	66.5
5	5	4	5	4	4	4	77.0	81.5	84.0
3	3	3	3	3	3	3	76.0	78.5	88.0
4	4	4	4	4	4	4	57.0	52.5	52.5
5	5	5	5	5	5	5	65.5	66.0	79.0
5	4	5	5	4	4	5	56.0	58.5	58.5
5	5	5	5	5	5	5	61.5	61.5	61.5
5	5	5	5	5	5	5	80.5	74.5	87.0
4	4	4	3	4	4	4	51.0	55.5	46.0
4	5	5	5	4	4	5	89.0	91.5	78.0
4	4	5	4	4	4	5	57.0	57.0	57.0
4	3	2	4	3	2	4	42.5	53.5	62.0
3	3	3	3	3	3	3	64.5	44.0	74.0
3	3	3	3	3	3	3	57.0	57.0	57.0
5	5	5	5	5	5	5	79.5	81.5	81.5
4	3	3	4	3	4	4	60.0	42.5	64.0
5	5	5	5	5	5	5	64.5	66.0	81.5
5	5	4	5	5	5	5	87.5	77.5	96.5
2	3	3	3	3	3	3	60.0	47.0	65.5
1	3	3	3	3	3	3	56.5	59.5	59.5
5	5	5	4	4	4	5	59.5	63.0	51.0
4	5	4	5	3	4	4	80.5	77.0	72.5
3	3	4	3	3	3	2	60.5	55.0	61.0
5	4	5	5	3	4	4	60.5	56.5	60.0

1	5	5	1	5	5	5	70.5	69.5	69.5
5	5	5	5	5	5	5	82.5	87.0	87.0
5	5	5	5	5	5	3	72.0	67.0	62.5
4	4	4	4	4	4	4	74.0	57.0	59.5
5	5	4	5	3	5	5	58.5	68.5	66.0
5	5	5	5	5	5	5	86.5	87.0	82.0
4	3	4	4	4	3	3	74.0	65.0	64.0
4	4	3	3	4	4	4	68.0	49.5	50.5
4	4	4	4	5	5	4	45.0	47.5	62.0
4	3	4	4	3	3	4	70.0	55.0	72.0
5	5	5	4	5	5	5	79.5	60.5	57.0
4	4	4	4	4	4	4	73.0	70.5	66.0
4	4	4	4	3	4	4	65.5	79.0	58.5
4	4	4	3	3	4	4	55.5	57.0	51.0
4	4	4	4	4	2	5	60.5	65.5	65.5
3	4	4	4	4	3	4	66.5	62.0	76.0
4	3	5	1	5	5	5	26.0	27.5	20.0
5	5	5	5	5	5	5	47.0	52.5	74.5
4	4	4	4	4	4	4	64.5	64.5	77.5
3	3	3	3	2	3	2	56.0	71.0	70.0
4	5	5	5	4	5	5	72.5	50.0	69.0
5	5	4	5	5	4	5	35.5	50.5	41.5
1	5	5	5	5	4	4	74.0	74.0	67.0
5	5	5	5	5	5	5	74.0	70.0	74.5
5	5	5	5	4	4	5	53.0	59.0	57.5
3	4	4	3	3	4	4	27.5	53.5	44.0
5	5	5	5	5	5	5	67.0	67.5	73.5
5	5	5	5	5	5	5	48.0	67.5	53.5
4	4	4	4	4	4	4	73.0	72.5	73.5
5	5	5	4	4	4	4	48.5	53.5	51.0
3	5	1	1	1	1	3	30.0	22.5	15.0
4	5	3	3	4	5	5	61.5	88.0	66.5
5	5	4	5	4	5	4	62.5	60.0	57.0
4	4	4	4	4	4	5	38.5	38.5	65.0
2	5	5	5	5	5	5	72.0	77.0	67.5
4	3	3	4	3	3	3	61.0	45.0	51.5
4	4	4	4	4	4	4	81.5	60.0	60.0
5	5	3	5	4	5	5	65.0	67.5	61.5
4	5	3	3	3	4	4	73.5	53.5	61.0
5	4	3	5	5	4	4	55.5	50.5	63.5
3	3	3	3	3	3	3	64.5	50.0	42.5
4	5	4	2	4	4	4	74.5	62.0	65.0
5	5	4	3	2	2	5	59.5	50.5	70.5
3	5	4	3	3	3	4	48.5	48.5	45.0
4	4	4	4	4	4	4	45.0	30.0	49.0
3	3	2	3	3	2	2	58.0	86.0	50.0
5	4	4	3	4	4	4	84.5	51.0	74.0
3	3	3	4	4	2	3	38.5	37.5	39.5
4	4	5	2	2	3	3	61.0	55.5	81.0
2	4	4	4	4	4	4	63.5	56.0	66.0

5	5	2	1	5	3	5	58.0	31.0	53.0
4	4	4	4	4	4	4	36.0	35.0	30.0
3	4	5	3	4	4	4	60.5	30.0	45.0
4	4	4	4	3	3	4	47.0	50.5	56.0
5	4	4	5	3	4	3	74.0	79.0	80.0
5	4	4	4	4	5	3	40.0	37.0	51.5
5	5	5	5	4	4	5	49.5	44.5	74.0
5	5	5	5	5	5	5	54.5	42.0	53.5
4	5	3	5	4	4	5	86.0	88.0	85.5
3	3	3	3	3	3	3	53.0	55.5	62.5
5	5	5	3	2	3	3	32.5	42.0	20.0
4	4	4	5	5	5	3	78.0	51.0	59.0
4	4	5	5	4	4	4	57.0	69.0	42.0
3	4	4	4	4	4	4	64.5	50.0	61.0
4	5	4	4	5	4	5	76.5	54.5	59.5
4	5	5	3	5	5	5	77.5	46.5	73.5
5	5	5	5	5	4	4	64.0	64.0	66.0
5	5	4	4	2	3	4	66.0	56.5	59.0
4	4	4	4	4	4	4	52.5	59.0	60.0
5	5	5	5	5	5	5	46.5	53.0	46.5
4	4	4	4	3	3	4	95.0	48.5	60.5
5	5	5	5	5	5	5	74.0	64.0	85.0
4	3	2	4	3	3	4	48.0	53.0	62.5
5	5	5	5	5	5	5	41.0	58.5	53.0
4	3	3	3	3	3	3	67.5	53.5	58.5
4	5	5	5	5	5	5	64.5	72.0	81.0
5	5	5	5	5	5	5	65.5	61.5	62.5
5	5	5	5	5	5	5	50.0	60.0	73.5
3	4	4	3	3	2	1	64.5	51.0	48.0
4	3	4	3	4	4	3	48.5	57.0	59.0
4	4	4	5	4	3	2	58.5	48.5	66.0
5	5	4	4	5	5	5	42.5	37.0	40.0
5	4	5	5	5	5	5	47.0	61.5	47.0
5	3	5	5	5	4	4	68.0	78.0	81.5
3	5	5	3	3	3	4	79.0	68.0	67.5
3	3	2	3	3	2	2	41.0	28.5	75.5
1	4	4	4	5	4	4	51.5	39.5	51.5
4	3	3	3	3	3	3	71.0	49.5	67.5
5	5	5	5	5	5	3	81.5	81.5	79.0
5	4	5	5	4	5	5	69.5	62.5	62.5
3	3	3	2	2	2	2	68.0	59.5	55.5
5	5	5	5	5	5	5	43.0	45.5	52.5
5	4	5	5	4	4	4	53.0	27.5	53.0
4	3	4	4	3	3	3	75.0	76.0	58.0
5	5	5	5	5	5	5	67.5	75.0	72.5
5	5	5	5	5	5	5	57.0	48.0	78.0
5	4	5	5	5	4	3	57.0	41.0	49.0
4	5	5	3	4	4	3	66.0	57.0	42.0
5	5	4	5	4	4	5	48.0	59.5	51.0
5	4	4	5	3	5	4	65.5	60.5	60.5

5	5	4	4	5	5	5	91.5	84.5	79.5
5	5	5	4	4	4	5	61.0	68.0	57.0
1	2	4	4	3	3	3	50.5	51.5	37.5
4	5	4	4	5	5	5	78.5	81.0	81.0
4	4	3	2	2	3	3	74.5	51.5	59.5
5	4	5	5	3	3	3	42.5	56.0	56.5
4	5	4	4	3	5	5	33.5	64.5	27.5
5	3	3	2	3	3	1	67.5	46.0	65.0
4	5	4	5	5	5	5	70.0	50.5	73.0
5	3	5	5	3	3	2	59.5	51.0	56.0
4	4	4	4	4	4	3	49.5	35.0	37.0
5	5	5	5	5	5	5	69.0	62.5	62.5
4	4	4	4	4	4	4	66.5	56.5	59.0
5	4	5	5	5	5	5	68.0	35.5	58.0
3	3	3	4	4	4	4	38.5	48.5	51.0
4	4	4	4	3	4	4	53.0	41.0	27.5
5	5	5	5	5	5	5	60.5	53.5	62.5
3	4	4	5	3	3	4	48.5	72.0	47.5
5	5	5	5	4	5	4	55.5	59.5	44.5
5	5	4	5	3	4	4	51.0	27.0	30.0
5	5	5	5	5	5	5	77.5	82.5	75.5
5	4	5	5	4	4	4	67.5	71.5	57.0
1	5	5	1	5	1	1	77.5	50.0	67.0
1	2	2	2	2	2	2	87.0	22.5	66.0
5	4	4	4	4	3	5	47.0	45.0	58.0
5	2	4	2	3	3	4	65.0	37.5	73.5
4	4	4	4	4	4	4	76.5	64.0	47.0
5	5	5	5	5	5	5	66.5	69.0	81.5
5	5	5	5	5	5	4	77.0	79.5	73.5
4	5	5	5	5	5	5	64.0	48.5	79.5
4	4	4	4	4	4	4	35.0	52.0	15.0
5	4	4	4	3	4	4	51.0	48.5	55.5
3	4	4	1	4	4	4	58.0	58.0	48.0
5	5	5	4	4	4	5	57.0	68.0	73.0
4	4	3	4	4	4	4	65.5	48.5	53.0
4	5	4	5	4	4	4	86.0	55.5	49.5
4	5	4	3	5	5	5	60.5	61.5	62.5
5	4	4	4	5	5	5	69.5	51.0	67.0
2	3	2	3	3	5	4	64.0	65.0	69.0
5	5	5	5	5	5	5	58.5	53.5	68.0
5	4	4	5	4	4	4	63.0	50.5	71.0
5	5	5	5	5	5	5	52.0	70.5	64.5
4	4	5	4	4	4	4	74.0	60.5	45.5
1	3	3	3	3	3	4	35.0	43.0	60.0
3	4	3	3	3	3	3	54.5	54.5	54.5
4	4	4	4	3	3	4	56.5	54.5	49.0
5	4	4	5	5	4	5	70.0	36.0	34.5
4	4	3	3	4	5	5	63.0	51.0	48.5
3	5	4	3	5	5	5	79.0	54.5	61.5
3	4	4	3	3	4	4	57.0	59.5	58.0

1	5	5	5	5	5	5	78.0	76.5	65.0
5	5	3	3	3	3	2	58.0	61.0	43.0
4	2	3	2	2	3	1	84.0	41.0	74.0
4	4	4	3	4	4	4	56.0	58.5	37.0
2	2	5	2	2	2	2	61.5	59.5	45.5
4	4	4	4	4	4	4	45.0	58.0	47.5
4	4	4	4	4	4	4	67.0	44.5	78.0
5	5	5	5	5	5	5	54.0	72.0	69.5
4	4	4	4	4	4	4	43.5	43.5	42.5
5	4	5	4	4	4	4	60.5	46.0	84.0
4	4	3	3	3	3	4	65.5	42.0	53.5
3	2	1	1	1	4	4	34.5	70.0	45.0
5	5	5	5	5	5	5	71.5	38.5	65.0
4	4	4	4	4	4	4	73.5	50.0	47.0
4	3	3	4	4	3	3	59.0	49.5	59.0
4	4	4	4	4	4	4	65.0	53.0	50.5
4	5	3	4	5	4	4	79.5	45.5	44.0
3	4	3	4	4	4	4	57.5	50.5	53.0
4	3	4	2	4	2	2	62.5	62.5	69.5
5	5	5	5	5	5	5	55.5	64.5	70.0
5	4	4	4	4	4	4	66.0	49.5	59.0
3	3	4	3	3	3	4	77.0	78.5	65.5
5	5	5	5	5	5	5	63.5	52.5	25.0
4	4	4	4	5	4	4	62.5	69.0	59.0
3	3	3	2	4	3	3	60.5	48.5	46.0
4	3	4	3	4	3	4	59.0	64.0	67.0
1	5	1	1	3	5	2	55.5	72.0	54.0
5	3	3	3	3	3	3	60.0	54.5	57.5
4	2	3	4	2	3	3	44.0	63.0	60.0
3	3	3	3	3	3	3	59.5	65.5	54.5
5	5	4	3	3	5	5	87.0	62.5	57.0
3	4	5	5	5	5	4	68.0	56.0	70.5
5	5	5	5	5	5	5	60.5	62.5	48.5
3	4	3	2	3	3	2	86.5	51.0	52.5
3	3	3	3	4	3	3	60.5	46.0	51.0
5	5	3	5	5	5	4	57.0	44.5	48.5
5	5	5	5	5	5	5	52.0	79.5	15.0
4	3	4	4	3	3	3	69.0	77.0	58.5
4	5	5	5	5	5	5	52.0	71.0	37.5
3	4	4	3	4	4	3	69.0	45.0	58.0
3	3	3	4	4	4	4	59.5	42.5	57.0
4	4	3	4	3	4	4	68.0	53.5	66.5
5	5	4	5	5	5	5	91.5	37.5	75.5
3	4	4	3	3	3	2	58.5	70.5	56.0
4	5	4	4	4	5	4	58.5	53.5	69.5
5	5	1	4	5	4	4	63.5	69.5	74.5
3	4	4	4	3	4	4	67.0	67.0	60.0
5	5	5	4	2	3	2	95.0	63.5	78.0
3	3	3	3	3	3	3	58.0	63.0	70.0
4	5	4	5	5	5	5	73.0	44.5	97.5

4	4	3	3	3	4	5	67.5	72.5	81.0
5	5	5	5	5	5	4	81.0	92.5	70.0
5	5	5	5	5	4	5	58.0	62.5	49.0
5	5	5	5	4	5	5	46.0	44.5	65.0
3	1	3	2	2	2	2	80.0	51.0	55.5
3	3	3	4	5	5	5	65.0	23.0	54.0
5	5	4	4	3	4	5	80.0	80.0	87.0
5	4	5	4	4	5	2	58.0	53.5	68.0
4	4	4	3	4	4	4	67.0	45.0	90.5
5	5	5	5	5	5	5	29.5	34.5	46.5
4	3	2	3	4	2	4	49.5	51.5	32.0
4	4	4	4	4	4	4	59.5	62.0	71.5
4	4	4	2	2	4	4	69.0	60.5	65.0
4	5	5	4	5	5	5	60.5	48.5	62.5
5	5	5	4	4	5	5	51.0	55.5	59.0
4	4	4	4	4	4	4	65.5	58.0	61.5
2	2	2	2	2	2	2	81.0	71.0	81.5
3	5	3	3	3	3	3	51.0	57.0	42.0
5	5	5	5	5	5	5	79.0	58.5	84.0
5	5	5	4	4	4	4	81.0	64.0	78.0
4	4	4	4	4	4	3	58.0	55.5	60.0
5	4	5	5	4	4	4	42.5	56.5	37.5
4	5	4	4	5	5	5	60.5	51.0	58.0
4	4	4	5	4	4	4	42.5	52.0	60.5
4	4	3	4	4	4	4	64.5	66.5	62.0
5	4	4	4	3	3	3	83.0	71.0	58.5
5	3	5	3	3	4	1	72.0	65.0	50.5
5	4	5	4	5	5	3	62.5	60.0	74.0
4	4	4	4	3	3	4	43.5	43.5	58.0
4	3	4	4	3	3	4	70.0	82.0	48.0
4	4	4	4	4	4	4	62.5	86.5	59.5
5	5	5	5	4	3	4	56.5	61.5	69.5
4	3	4	3	3	3	2	52.0	35.0	57.0
1	1	1	1	2	2	1	41.5	61.5	46.0
4	4	4	3	4	4	4	51.5	45.5	57.0
3	3	3	3	4	3	4	37.5	50.0	20.0
5	4	4	4	5	4	4	55.5	48.5	58.0
2	4	4	4	4	4	4	83.0	57.5	75.5
5	5	5	5	5	5	5	52.0	36.0	42.0
3	1	4	1	3	3	3	46.0	48.5	53.5
4	4	4	4	4	4	4	56.5	52.0	58.5
5	3	4	3	3	3	3	69.0	69.0	66.5
4	3	3	3	2	3	4	30.0	32.5	52.0
3	4	4	4	4	4	4	81.5	86.5	49.5
4	4	3	3	2	2	4	54.0	47.5	47.0
4	4	4	3	3	4	3	72.5	59.0	57.5
4	4	4	4	4	4	4	79.5	78.5	61.5
4	5	4	5	2	3	4	74.5	68.0	49.5
3	2	3	2	2	2	2	49.5	43.5	64.5
3	4	3	3	3	3	4	53.5	46.0	58.0

5	5	5	4	5	5	4	37.5	36.0	37.5
5	5	5	4	4	4	4	54.5	66.0	53.0
3	3	3	3	3	3	3	64.5	53.5	75.0
4	4	3	4	5	4	5	61.5	64.5	55.5
4	4	5	2	3	3	2	60.5	42.5	37.0
5	4	5	5	4	4	4	64.0	74.5	78.0
4	4	4	4	4	3	4	31.0	52.0	62.5
5	5	5	5	4	4	5	75.5	65.5	92.0
4	3	4	4	3	4	5	86.0	82.5	84.5
5	5	5	5	5	5	5	50.5	50.0	68.0
4	5	5	5	3	5	5	80.5	50.5	73.0
5	4	3	5	3	4	1	76.0	72.5	71.0
4	4	4	4	4	4	4	51.5	61.5	54.0
3	3	3	3	3	3	3	41.0	54.0	55.5
5	5	4	4	5	5	5	49.5	37.5	42.0
5	3	5	3	3	4	3	70.0	67.5	70.0
5	5	5	5	5	3	3	56.0	25.0	53.5
4	4	4	4	4	4	3	46.0	42.0	62.0
4	4	2	3	3	4	4	66.5	66.0	60.0
5	4	4	3	4	2	4	48.0	48.0	50.5
5	4	4	5	5	4	5	65.0	48.0	49.5
2	1	3	2	1	3	4	44.0	38.0	57.5
4	5	4	4	5	5	4	61.0	69.5	49.5
4	4	4	4	4	4	3	56.0	59.5	78.0
4	4	4	4	4	4	4	78.5	59.0	77.5
5	5	5	5	5	5	5	70.0	69.0	76.0
5	4	5	5	5	4	4	60.5	48.0	70.0
4	4	4	5	3	4	4	64.5	58.0	61.0
5	4	4	5	4	4	4	62.0	44.0	56.5
2	4	4	4	4	4	4	55.5	53.0	58.0
3	5	4	4	4	5	5	50.5	49.5	42.5
3	3	3	4	5	5	5	59.5	45.0	49.5
5	5	5	5	5	5	5	68.5	54.5	70.0
5	4	5	5	4	4	4	25.0	46.0	69.5
3	4	3	3	4	4	4	79.5	63.5	72.0
5	4	4	4	4	4	4	60.5	72.5	58.0
5	3	5	5	3	3	4	57.0	57.0	61.0
5	5	5	5	4	4	4	72.5	68.0	52.0
4	4	4	4	4	4	4	62.5	67.5	74.0
4	4	3	3	4	4	3	43.5	43.5	35.0
3	4	4	4	3	3	5	69.0	74.0	80.5
3	3	3	3	3	3	3	27.5	26.0	44.5
5	5	5	3	5	5	5	38.5	64.0	39.5
5	4	3	3	3	4	5	73.5	48.5	59.0
5	5	5	5	5	5	5	56.0	56.0	33.5
4	4	4	4	4	3	4	69.0	62.0	73.5
3	4	4	4	4	4	4	62.0	57.0	57.0
3	3	4	4	3	2	3	48.5	48.5	40.0
4	4	4	4	4	4	4	69.5	58.0	60.0
3	3	4	2	4	4	4	41.0	33.5	52.5

4	4	4	4	4	4	4	62.5	54.0	72.0
5	5	5	3	5	5	5	70.0	70.0	64.5
5	5	5	3	5	5	5	89.0	49.5	69.0
5	5	3	5	5	5	5	73.0	47.5	56.0
5	4	5	5	4	4	5	56.0	65.5	62.5
4	4	4	4	2	2	4	77.0	79.5	71.0
4	5	4	4	4	4	5	56.0	58.5	58.5
4	3	2	4	3	3	3	71.5	68.0	64.0
4	4	4	4	4	4	4	63.0	50.5	56.5
2	4	4	3	4	5	5	60.0	25.0	60.0
3	3	4	4	3	4	4	42.5	45.0	27.5
5	5	5	5	5	5	5	63.0	63.0	73.0
4	5	4	5	4	4	4	62.5	59.5	55.5
2	4	4	2	4	4	4	60.5	51.0	55.0
5	5	5	5	5	5	5	64.0	72.0	57.5
5	5	4	5	5	5	4	45.5	53.0	69.5
5	4	5	5	3	3	5	75.5	47.0	52.0
5	5	4	4	4	4	5	47.0	46.0	63.5
3	4	4	4	4	4	4	50.0	44.5	57.5
3	5	5	3	5	5	5	62.5	48.5	76.0
5	5	5	5	5	5	5	67.5	53.0	74.5
5	4	3	4	4	4	5	70.5	40.0	74.0
4	3	3	4	2	3	4	77.0	61.0	59.0
3	3	3	3	3	3	3	66.0	78.5	78.5
5	1	4	4	2	2	1	71.0	41.0	67.5
5	5	5	5	5	5	5	56.5	44.0	44.5
5	5	5	5	5	5	5	71.0	75.0	69.0
4	4	1	3	3	3	3	58.0	60.0	53.5
4	5	4	3	4	4	5	55.5	72.0	49.5
4	4	5	4	4	5	5	59.5	76.5	53.5
5	5	5	5	5	5	5	81.5	77.0	75.5
2	3	2	2	2	2	3	56.0	30.0	47.0
5	5	4	4	4	4	4	58.5	81.5	79.0
5	5	5	5	4	3	3	67.0	46.0	37.5
4	4	4	4	4	4	4	51.0	59.5	86.0
3	4	4	3	3	5	5	64.0	60.5	67.0
3	5	4	4	5	5	5	46.0	57.0	47.0
5	5	5	5	5	5	5	78.5	48.5	63.5
5	5	5	5	4	5	5	35.0	60.0	37.0
4	4	4	4	4	4	4	52.5	68.5	79.5
5	2	2	2	2	2	2	71.0	76.0	63.5
3	4	4	4	4	4	4	50.0	45.0	40.0
5	4	5	3	3	3	1	75.0	64.5	73.5
3	5	4	4	4	5	5	72.5	62.0	66.5
5	4	5	3	3	4	5	65.5	48.0	85.5
5	5	5	5	5	5	3	64.0	48.5	55.5
5	4	3	3	4	3	4	83.5	85.5	73.5
5	3	5	5	2	2	4	67.0	66.0	63.5
5	5	2	5	4	5	4	60.5	65.5	70.0
3	2	3	3	3	2	3	46.5	35.0	72.0

5	4	4	4	4	4	4	46.0	51.0	60.5
4	4	4	4	4	3	3	56.5	66.5	67.0
5	5	5	5	5	5	5	54.0	67.5	54.0
5	4	4	4	4	4	4	53.0	48.0	54.5
5	4	4	4	4	4	5	56.0	48.5	60.5
4	4	4	4	5	5	5	72.0	72.5	52.5
5	5	4	5	5	5	5	83.0	77.5	74.0
5	3	5	5	3	3	5	58.5	45.0	53.5
5	5	5	5	5	5	5	50.0	48.0	43.5
3	3	5	3	3	3	3	52.0	66.0	74.5
5	4	4	5	4	5	3	76.5	47.5	66.0
5	5	4	3	5	5	5	51.5	56.5	56.5
4	4	4	5	4	4	4	62.5	48.5	76.5
5	5	5	5	4	4	5	56.5	56.5	49.0
3	3	3	3	3	3	3	64.5	58.5	76.5
5	3	5	5	3	3	2	58.0	63.5	83.0
1	1	2	1	3	3	1	66.5	43.0	50.0
5	5	5	5	5	5	5	81.5	70.5	68.0
4	3	4	3	2	2	4	54.5	54.5	45.0
3	3	4	3	3	4	4	84.5	82.5	71.5
4	4	4	4	4	4	4	74.5	77.0	63.0
3	4	4	3	4	3	3	82.0	48.0	54.0
3	4	3	4	3	4	3	82.5	64.5	80.0
5	4	2	1	1	1	4	75.0	66.0	59.0
5	5	5	5	5	5	5	84.5	84.5	83.0
5	4	4	4	4	4	4	58.0	31.0	59.0
3	3	4	3	3	3	3	55.0	43.5	62.5
1	2	4	4	4	4	5	69.5	33.5	59.0
4	4	4	4	4	3	3	70.0	65.5	55.5
5	4	2	5	4	2	4	57.5	32.5	56.0
4	4	4	4	4	4	4	65.5	68.0	62.5
5	5	5	5	5	5	4	82.0	70.5	77.5
4	5	3	3	5	5	5	48.0	51.5	79.5
5	5	5	5	4	5	4	68.0	55.0	65.0
3	4	3	4	4	3	4	46.5	46.5	61.0
4	4	4	5	5	5	5	64.0	57.0	63.0
3	5	5	4	5	5	5	77.5	71.0	75.0
2	3	2	2	3	3	4	50.5	50.5	46.5
5	5	4	2	3	4	3	59.0	67.5	74.0
4	3	3	3	3	3	3	50.5	56.5	60.5
5	4	4	3	3	4	4	52.5	54.5	46.0
5	5	5	5	5	5	5	73.5	80.0	84.5
5	5	5	5	5	5	5	70.0	75.5	65.0
3	4	4	4	4	2	2	85.5	78.0	83.5
5	5	3	3	3	4	4	65.0	41.0	53.0
5	3	3	4	3	4	3	74.0	55.5	76.5
5	3	5	5	2	3	2	32.5	56.0	63.0
5	5	4	4	5	4	5	71.0	62.5	52.5
3	4	3	4	4	4	4	78.0	78.0	68.0
4	4	4	3	3	3	3	59.5	59.5	63.0

2	2	1	2	4	5	5	35.0	32.5	46.5
4	4	3	4	4	3	3	77.0	76.0	57.0
5	5	5	4	4	4	4	63.0	62.5	65.0
4	5	5	5	4	5	5	38.5	17.5	47.5
5	5	5	5	5	5	5	51.0	51.0	48.5
4	5	4	4	4	4	4	33.5	53.0	46.0
5	4	4	4	4	4	4	75.0	77.5	79.5
4	4	4	4	4	4	4	71.0	71.0	74.5
5	4	5	5	5	5	5	54.0	50.0	47.5
4	4	4	4	4	4	4	73.5	63.0	67.5
4	4	4	4	3	3	4	70.5	60.5	84.0
5	5	5	5	5	5	5	58.0	78.5	30.0
5	5	3	3	4	4	4	83.5	67.5	77.0
5	4	4	4	5	5	5	62.0	45.5	67.0
3	3	4	2	3	3	3	60.0	70.0	85.5
4	4	4	3	3	3	3	61.0	46.0	72.5
5	5	5	4	4	4	4	53.0	60.0	80.5
4	2	4	2	2	3	4	70.0	45.0	62.5
4	5	4	3	4	4	4	48.0	65.0	67.5
4	4	5	5	5	4	3	67.0	44.0	64.0
1	3	5	3	3	5	5	77.0	86.0	79.5
4	3	4	2	3	4	4	63.0	69.5	67.0
4	4	3	3	3	4	3	71.5	53.0	62.0
5	5	4	4	4	4	4	73.5	61.0	71.0
4	4	4	4	4	4	5	75.0	77.5	59.0
5	5	5	5	5	5	5	53.0	60.0	55.0
4	4	3	3	4	3	4	72.5	43.5	65.5
3	3	4	2	4	4	3	48.5	61.0	64.5
4	4	3	5	5	5	5	60.0	53.5	67.5
4	4	4	3	3	4	3	75.0	69.5	72.0
5	5	4	4	4	4	4	57.5	57.0	74.0
5	5	5	5	5	5	5	74.5	62.5	72.0
3	4	4	5	1	1	1	63.0	53.0	74.0
4	4	4	4	4	5	4	71.5	67.0	69.0
5	4	4	5	4	4	5	84.5	69.5	70.0
5	5	5	5	5	5	5	72.0	61.5	64.5
5	5	3	4	5	5	3	71.5	50.0	69.5
4	3	3	3	3	3	3	71.0	57.0	56.0
5	4	3	3	2	2	3	56.5	58.0	68.5
5	5	5	5	5	5	3	64.0	54.0	47.0
4	5	5	5	5	5	5	67.5	65.0	56.0
5	5	5	5	5	5	5	47.0	51.5	51.5
4	4	5	4	5	5	3	60.5	64.0	54.0
4	4	3	4	4	4	4	71.5	65.5	74.5
5	5	5	5	5	5	5	47.0	32.5	45.0
3	5	4	5	4	5	5	56.5	53.0	53.0
5	5	3	4	5	4	3	49.5	75.5	43.5
4	3	4	4	3	4	3	47.5	44.5	60.5

NSE_3	Ciudad
2	1
2	1
2	1
3	1
2	1
2	1
3	1
2	1
2	1
3	1
2	1
2	1
2	1
2	1
2	1
1	1
2	1
2	1
2	1
2	1
2	1
2	1
2	1
2	1
2	1
3	1
2	1
2	1
2	1
2	1
2	1
2	1
2	1
3	1
2	1
3	1
2	1
2	1
3	1
2	1
2	1
2	1
2	1
3	1
2	1
2	1
2	1
2	1
1	1

2	1
2	1
2	1
2	1
2	1
2	1
3	1
2	1
2	1
2	1
3	1
2	1
2	1
2	1
2	1
2	1
2	1
2	1
2	1
2	1
2	1
2	1
2	1
2	1
2	1
2	1
2	1
2	1
2	1
2	1
2	1
2	1
2	1
2	1
2	1
2	1
2	1
2	1
3	1
2	1
2	1
2	1
2	1
2	1
2	1
2	1
2	1
2	1
2	1
2	1

2	1
2	1
2	1
2	1
3	1
2	1
2	1
2	1
2	1
2	1
3	1
3	1
2	1
3	1
3	1
3	1
2	1
2	1
2	1
1	1
2	1
2	1
2	1
1	1
2	1
2	1
2	1
3	1
3	1
2	1
2	1
2	1
2	1
3	1
2	1
2	1
2	1
2	1
3	1
2	1
2	1
2	1
2	1
2	1
2	1
2	1
2	1
2	1
2	1
2	1

2	1
2	1
2	1
2	1
2	1
2	1
2	1
2	1
3	1
2	1
2	1
2	1
3	1
3	1
2	1
2	1
1	1
2	1
2	1
2	1
2	1
2	1
3	1
2	1
2	1
2	1
2	1
2	1
3	1
2	1
2	1
2	1
2	1
2	1
2	1
2	1
2	1
2	1
2	1
3	1
2	1
2	1
2	1
2	1
2	1
2	1
2	1
2	1
2	1
3	1

3	1
2	1
3	1
2	1
2	1
2	1
2	1
2	1
2	1
3	1
2	1
2	1
2	1
3	1
2	1
2	1
2	1
2	1
3	1
2	1
3	1
3	1
2	1
2	1
3	1
2	1
2	1
2	1
2	1
2	1
2	1
2	1
3	1
2	1
2	1
2	1
2	1
2	1
2	1
2	1
3	1
2	1
2	1
2	1
2	1
2	1
2	1
2	1
3	1
3	1

2	1
3	1
2	1
1	1
3	1
2	1
2	1
2	1
2	1
2	1
2	1
2	1
2	1
2	1
2	1
2	1
2	1
2	1
3	1
2	1
2	1
2	1
2	1
2	1
1	1
3	1
2	1
2	1
2	1
2	1
2	1
2	1
2	1
2	1
2	1
3	1
3	1
2	1
2	1
2	1
2	1
2	1
2	1
2	1
2	1
3	1
2	1
2	1
2	1
2	1

2	1
2	1
2	1
2	1
2	1
3	1
2	1
2	1
2	1
2	1
2	1
2	1
2	1
2	1
2	1
2	1
2	1
3	1
3	1
3	1
3	1
3	1
3	1
2	1
3	1
2	1
2	1
3	1
2	1
2	1
2	1
2	1
2	1
2	1
2	1
2	1
2	1
3	1
2	1
2	1
2	1
2	1
2	1
2	1
3	1
2	1
3	1
2	1
2	1
2	1

2	1
2	1
2	1
2	1
2	1
2	1
2	1
2	1
3	1
2	1
2	1
2	1
3	1
3	1
3	1
2	1
2	1
3	1
2	1
2	1
2	1
2	1
2	1
2	1
3	1
2	1
2	1
2	1
2	1
2	1
3	1
2	1
2	1
2	1
2	1
2	1
3	1
2	1
2	1
3	1
3	1
2	1
3	1
2	1
2	1
2	1
2	1
2	1
2	1
2	1

2	1
3	1
2	1
2	1
2	1
3	1
2	1
2	1
2	1
2	1
2	1
2	1
3	1
2	1
2	1
2	1
2	1
2	1
2	1
2	1
2	1
2	1
2	1
3	1
2	1
2	1
2	1
3	1
2	1
2	1
2	1
3	2
3	2
2	2
2	2
2	2
3	2
2	2
3	2
2	2
2	2
2	2
3	2
2	2
2	2
2	2
3	2
2	2
2	2
2	2

2	2
2	2
3	2
3	2
2	2
3	2
2	2
2	2
3	2
1	2
2	2
3	2
3	2
2	2
2	2
2	2
2	2
2	2
2	2
3	2
3	2
2	2
2	2
2	2
2	2
2	2
2	2
1	2
1	2
2	2
3	2
2	2
2	2
2	2
3	2
2	2
2	2
2	2
2	2
2	2
3	2
2	2
2	2
3	2
3	2
2	2
2	2
2	2
2	2
3	2

3	2
2	2
2	2
2	2
3	2
2	2
2	2
2	2
2	2
2	2
2	2
2	2
2	2
3	2
2	2
2	2
2	2
2	2
2	2
2	2
2	2
2	2
3	2
3	2
3	2
3	2
2	2
2	2
3	2
3	2
2	2
2	2
2	2
3	2
2	2
3	2
3	2
2	2
2	2
2	2
2	2
2	2
3	2
2	2
2	2
3	2
3	2
2	2
2	2
2	2

2	2
3	2
2	2
2	2
3	2
2	2
3	2
2	2
2	2
2	2
3	2
2	2
3	2
3	2
2	2
2	2
2	2
2	2
3	2
2	2
2	2
2	2
2	2
3	2
2	2
2	2
2	2
2	2
2	2
3	2
2	2
3	2
2	2
3	2
2	2
2	2
3	2
3	2
2	2
2	2
3	2
2	2
3	2
3	2
2	2
2	2
2	2
3	2
2	2
3	2

3	2
3	2
2	2
2	2
3	2
2	2
3	2
2	2
2	2
2	2
2	2
2	2
2	2
2	2
2	2
2	2
3	2
2	2
2	2
2	2
2	2
2	2
2	2
1	2
2	2
2	2
3	2
2	2
2	2
2	2
3	2
2	2
2	2
2	2
2	2
1	2
2	2
2	2
2	2
2	2
2	2
2	2
2	2
3	2
3	2
2	2
2	2
3	2
2	2
2	2

2	2
2	2
2	2
2	2
3	2
3	2
2	2
2	2
3	2
2	2
3	2
2	2
2	2
2	2
2	2
3	2
2	2
2	2
2	2
2	2
2	2
2	2
2	2
2	2
2	2
2	2
2	2
1	2
2	2
2	2
2	2
2	2
2	2
2	2
2	2
2	2
2	2
2	2
2	2
2	2
2	2
1	2
2	2
2	2
2	2
2	2
2	2
2	2
3	2
2	2

3	2
2	2
2	2
3	2
2	2
2	2
2	2
2	2
3	2
2	2
3	2
2	2
2	2
2	2
3	2
2	2
2	2
3	2
2	2
2	2
2	2
3	2
2	2
2	2
3	2
2	2
3	2
2	2
3	2
2	2
2	2
3	2
3	2
2	2
2	2
2	2
2	2
2	2
2	2
3	2
2	2
2	2
2	2
3	2
2	2
2	2
2	2
3	2
2	2
2	2

2	2
2	2
2	2
2	2
3	2
2	2
2	2
2	2
1	2
2	2
2	2
3	2
3	2
2	2
2	2
3	2
3	2
3	2
2	2
3	2
3	2
2	2
3	2
3	2
3	2
2	2
2	2
2	2
3	2
3	2
3	2
2	2
3	2
2	2
2	2
2	2
2	2
2	2
3	2
2	2
2	2
2	2
2	2
3	2
2	2
3	2
2	2
2	2
2	2
2	2

2	2
2	2
3	2
2	2
2	2
2	2
2	2
2	2
2	2
2	2
3	2
3	2
2	2
2	2
2	2
2	2
2	2
2	2
2	2
2	2
3	2
3	2
3	2
3	2
2	2
3	2
3	2
2	2
2	2
3	2
2	2
2	2
2	2
2	2
2	2
3	2
2	2
2	2
2	2
3	2
2	2
2	2
2	2
2	2
2	2
2	2
2	2
3	2
